# Supplementary material for: Macrophages are activated toward phagocytic lymphoma cell clearance by pentose phosphate pathway inhibition
Source: Cell Rep Med. 2024 Nov 26;5(12):101830. doi: 10.1016/j.xcrm.2024.101830 (PMC11722127; doi:10.1016/j.xcrm.2024.101830)
Supplement: Document S1. Figures S1–S7 and Table S6 — Table S6. Qualifier and quantifier transition of metabolites measured by targeted LC-QqQ/MS analysis, related to Figure 5. [file mmc1.pdf]

**Cell Reports Medicine, Volume *5***

## Supplemental information

# Macrophages are activated toward phagocytic lymphoma cell clearance by pentose phosphate pathway inhibition

**Anna C. Beielstein, Elena Izquierdo, Stuart Blakemore, Nadine Nickel, Michael Michalik, Samruddhi Chawan, Reinhild Brinker, Hans-Henrik Bartel, Daniela Vorholt, Lukas Albert, Janica L. Nolte, Rebecca Linke, Carolina Raíssa Costa Picossi, Jorge Sáiz, Felix Picard, Alexandra Florin, Jörn Meinel, Reinhard Büttner, Paul Diefenhardt, Sebastian Brähler, Alma Villaseñor, Holger Winkels, Michael Hallek, Marcus Krüger, Coral Barbas, and Christian P. Pallasch**

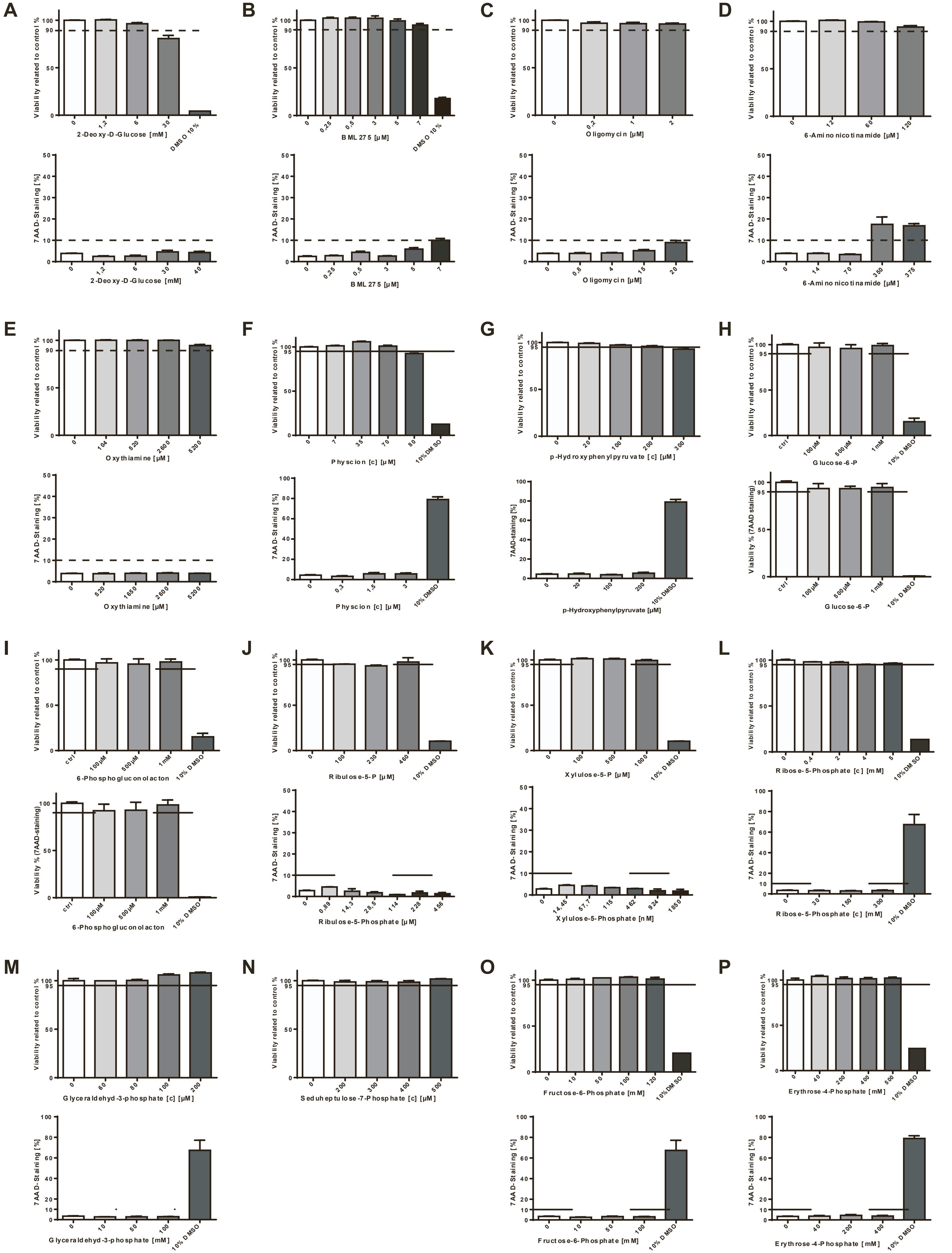
A
Viability related to control %
2-Deoxy-D-Glucose [mM]
DMSO 10%
7AAD-Staining [%]
B
BML 275 [µM]
C
Oligomycin [µM]
D
6-Aminonicotinamide [µM]
E
Oxythiamine [µM]
F
Physcion [c] [µM]
10% DMSO
G
p-Hydroxyphenylpyruvate [c] [µM]
p-Hydroxyphenylpyruvate [µM]
7AAD-staining [%]
H
Glucose-6-P
Viability % (7AAD staining)
I
6-Phosphogluconolacton
Viability % (7AAD-staining)
J
Ribulose-5-P [µM]
Ribulose-5-Phosphate [µM]
K
Xylulose-5-P [µM]
Xylulose-5-Phosphate [nM]
L
Ribose-5-Phosphate [c] [mM]
M
Glyceraldehyd-3-phosphate [c] [µM]
Glyceraldehyd-3-phosphate [mM]
N
Seduheptulose-7-Phosphate [c] [µM]
O
Fructose-6-Phosphate [mM]
P
Erythrose-4-Phosphate [mM]

**Figure S1. Evaluation of cytotoxicity of used compounds in J774A.1 macrophages and hMB cells. Related to Figures 1-2.**

**(A-P)** Measurement of viable cells under treatment with different inhibitors. Treatment with 10% DMSO used as positive control. Viability of J774A.1 cells (upper plots) was determined by Zombie staining, viability of hMB cells was determined by 7AAD staining. Viability under inhibition was compared to viability of untreated control cells. Used Inhibitors **A** 2-deoxy-D-glucose, **B** BML275, **C** oligomycin, **D** 6-aminonicotinamide, **E** oxythiamine, **F** physcion, **G** p-hydroxyphenylpyruvate, **H** glucose-6-phosphate, **I** 6-phosphogluconolactone, **J** ribulose-6-phosphate, **K** xylulose-5-phosphate, **L** ribose-5-phosphate, **M** glyceraldehyde-3-phosphate, **N** sedoheptulose-7-phosphate, **O** fructose-6-phosphate, **P** erythrose-4-phosphate.

Technical replicates n=4-12; biological replicates n=2-6. Data are shown as mean ± SEM. *P* values were calculated using one-way ANOVA. *$p < 0.05$; **$p < 0.01$; ***$p < 0.001$; ****$p < 0.0001$.

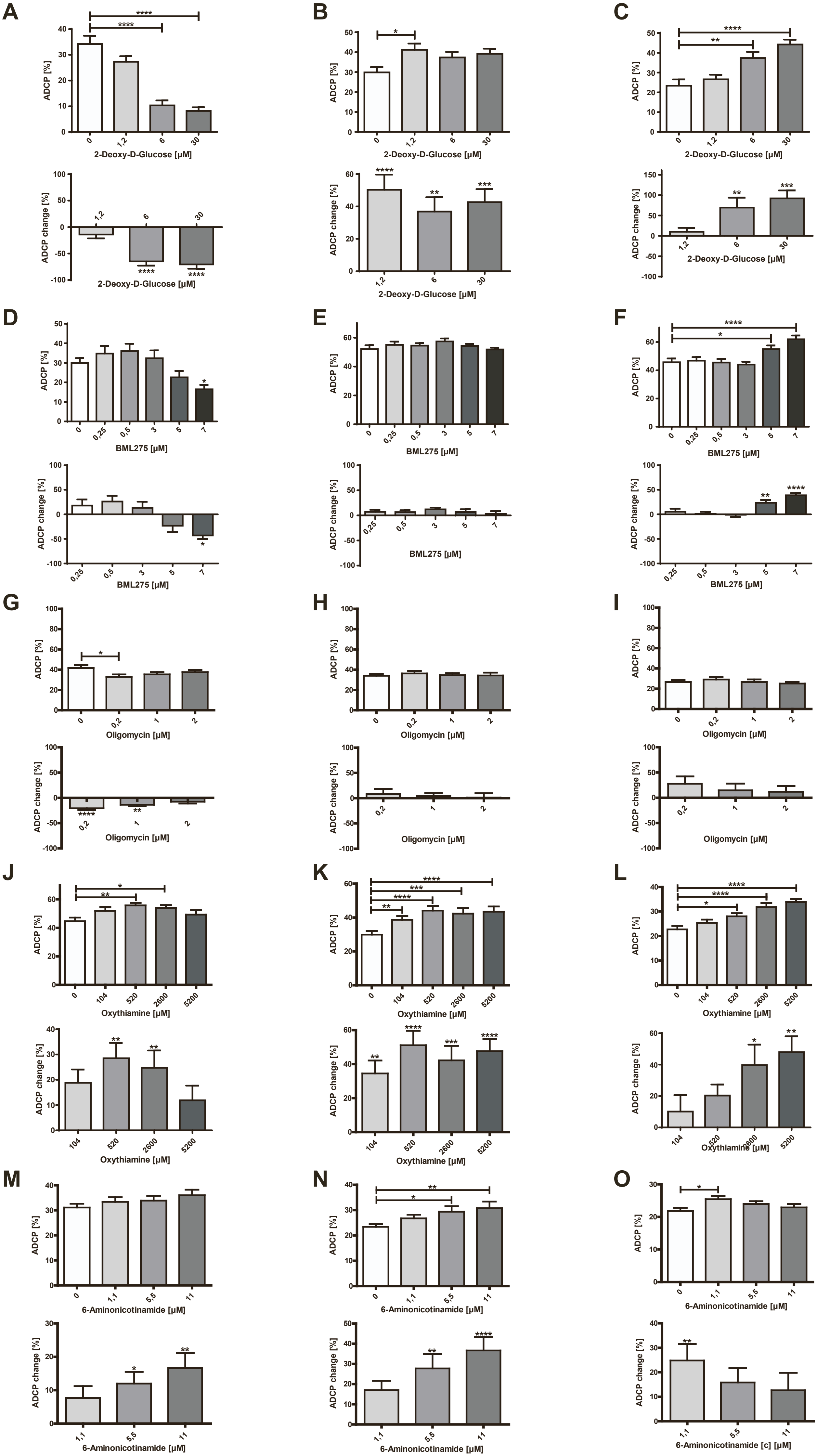
A
ADCP [%]
2-Deoxy-D-Glucose [µM]
ADCP change [%]
2-Deoxy-D-Glucose [µM]
B
ADCP [%]
2-Deoxy-D-Glucose [µM]
ADCP change [%]
2-Deoxy-D-Glucose [µM]
C
ADCP [%]
2-Deoxy-D-Glucose [µM]
ADCP change [%]
2-Deoxy-D-Glucose [µM]
D
ADCP [%]
BML275 [µM]
ADCP change [%]
BML275 [µM]
E
ADCP [%]
BML275 [µM]
ADCP change [%]
BML275 [µM]
F
ADCP [%]
BML275 [µM]
ADCP change [%]
BML275 [µM]
G
ADCP [%]
Oligomycin [µM]
ADCP change [%]
Oligomycin [µM]
H
ADCP [%]
Oligomycin [µM]
ADCP change [%]
Oligomycin [µM]
I
ADCP [%]
Oligomycin [µM]
ADCP change [%]
Oligomycin [µM]
J
ADCP [%]
Oxythiamine [µM]
ADCP change [%]
Oxythiamine [µM]
K
ADCP [%]
Oxythiamine [µM]
ADCP change [%]
Oxythiamine [µM]
L
ADCP [%]
Oxythiamine [µM]
ADCP change [%]
Oxythiamine [µM]
M
ADCP [%]
6-Aminonicotinamide [µM]
ADCP change [%]
6-Aminonicotinamide [µM]
N
ADCP [%]
6-Aminonicotinamide [µM]
ADCP change [%]
6-Aminonicotinamide [µM]
O
ADCP [%]
6-Aminonicotinamide [µM]
ADCP change [%]
6-Aminonicotinamide [c] [µM]

**Figure S2. Metabolic modulation changes antibody-dependent cellular phagocytosis (ADCP) of hMB cells by macrophages.**

**Related to Figure 1.**

**(A-O)** ADCP rate and ADCP rate compared to basal phagocytosis rate (=ADCP change) under treatment with metabolic inhibitors in a co-culture of J774A.1 macrophages and hMB cells under antibody treatment with alemtuzumab. **A, D, G, J, M** J774A.1 macrophages pre-treated with metabolic inhibitor, **B, E, H, K, N** inhibitor treatment of the co-culture, **C, F, I, L, O** hMB cells pre-treated with metabolic inhibitor. Used inhibitors **A-C** 2-deoxy-D-glucose, **D-F** BML275, **G-I** oligomycin, **J-L** oxythiamine, **M-O** 6-aminonicotinamide.

Technical replicates n=15-58, biological replicates n=3-12. Data are shown as mean ± SEM. *P* values were calculated using one-way ANOVA. *$p < 0.05$; **$p < 0.01$; ***$p < 0.001$; ****$p < 0.0001$.

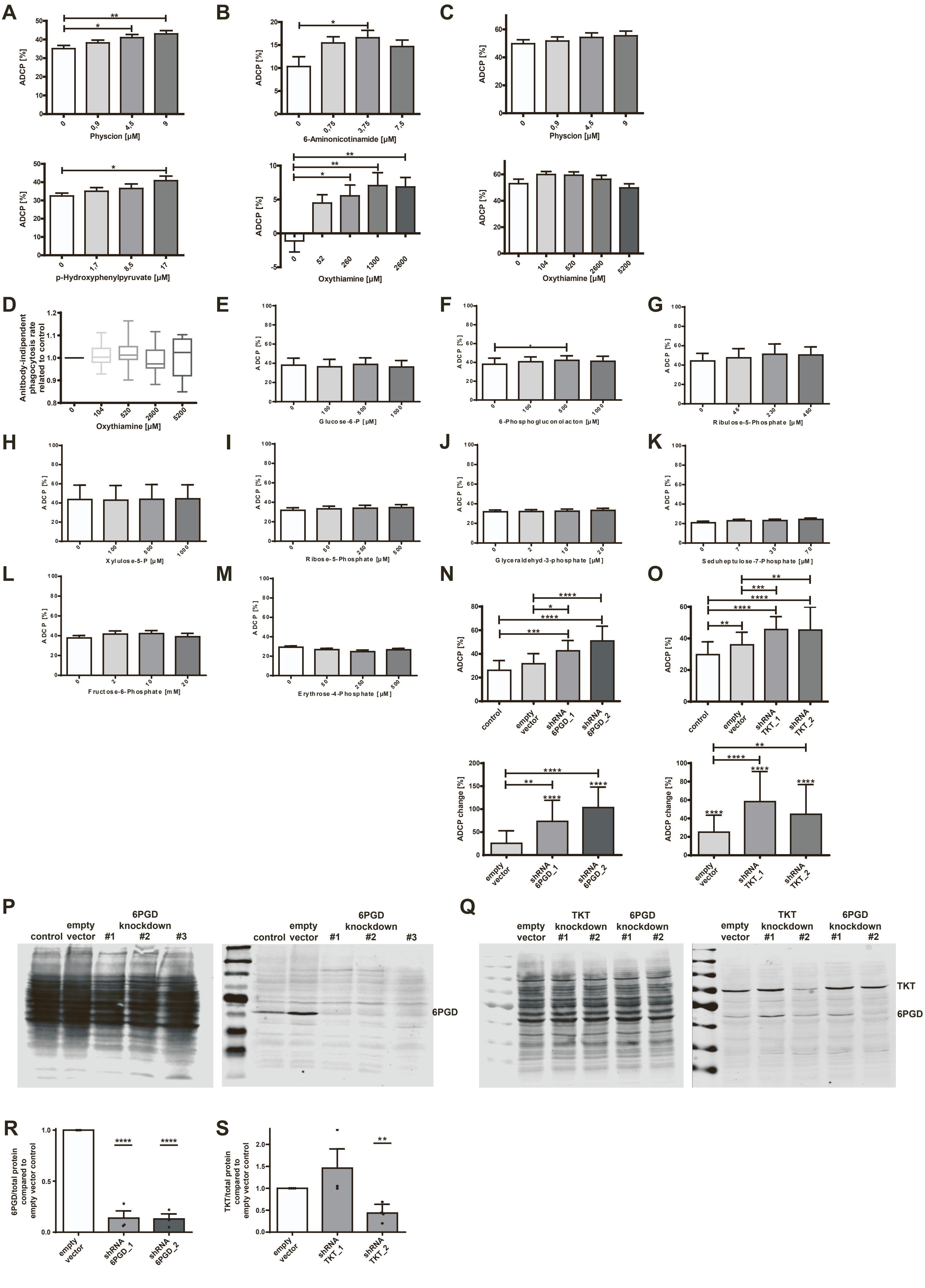
A
ADCP [%]
Physcion [µM]
p-Hydroxyphenylpyruvate [µM]
B
6-Aminonicotinamide [µM]
Oxythiamine [µM]
C
Physcion [µM]
Oxythiamine [µM]
D
Anitbody-indipendent phagocytosis rate related to control
Oxythiamine [µM]
E
Glucose-6-P [µM]
F
6-Phosphogluconolacton [µM]
G
Ribulose-5-Phosphate [µM]
H
Xylulose-5-P [µM]
I
Ribose-5-Phosphate [µM]
J
Glyceraldehyd-3-phosphate [µM]
K
Seduheptulose-7-Phosphate [µM]
L
Fructose-6-Phosphate [mM]
M
Erythrose-4-Phosphate [µM]
N
control
empty vector
shRNA 6PGD_1
shRNA 6PGD_2
ADCP change [%]
O
shRNA TKT_1
shRNA TKT_2
P
6PGD knockdown
6PGD
Q
TKT knockdown
TKT
R
6PGD/total protein compared to empty vector control
S
TKT/total protein compared to empty vector control

**Figure S3. PPP modulation changes ADCP of hMB cells by macrophages.**

**Related to Figure 2.**

**(A, C)** ADCP rate and ADCP rate compared to basal phagocytosis rate (=ADCP change) under treatment with metabolic inhibitors in a co-culture of J774A.1 macrophages and hMB cells under antibody treatment with alemtuzumab. **A** ADCP performed under PPP inhibition with physcion or p-hydroxyphenylpyruvate, **B** ADCP performed with THP1 monocytes and hMB cells under antibody treatment with obinutuzumab and PPP inhibition with 6-aminonicotinamide or oxythiamine. **C** ADCP assay performed in hypoxia under PPP inhibition with physcion or oxythiamine. **D** antibody-independent cellular phagocytosis (AiCP) rate of hMB cells by J744A.1 macrophages under treatment with oxythiamine. **(E-M)** ADCP rate under supplementation of PPP intermediates. **E** glucose-6-phosphate, **F** 6-phosphogluconolactone, **G** ribulose-5-phosphate, **H** xylulose-5-phosphate, **I** ribose-5-phosphate, **J** glyceraldehyde-3-phosphate, **K** sedoheptulose-7-phosphate, **L** fructose-6-phosphate, **M** erythrose-4-phosphate. **(N-O)** ADCP rate and ADCP rate compared to basal phagocytosis rate (=ADCP change) of hMB cells by shRNA mediates PPP knockdown macrophages**. N** shRNA mediated knockdown of 6-phosphogluconate dehydrogenase, **O** shRNA mediated knockdown of transketolase. **(P)** One representative example of western blot analysis of J744A.1 macrophages transfected with empty vector control and shRNA targeting 6-phosphogluconate dehydrogenase. Total protein stain and staining of 6-phosphogluconate dehydrogenase. **(Q)** One representative example of western blot analysis of J744A.1 macrophages transfected with empty vector control and shRNA targeting transketolase. Total protein stain and staining of transketolase. **(R)** Western blot analysis of 6pgd expression in J774A.1 macrophages under shRNA mediated PPP knockdown of 6Pgd compared to empty vector control. **(S)** Western blot analysis of Tkt expression in J774A.1 macrophages under shRNA mediated PPP knockdown of 6Pgd compared to empty vector control.

Technical replicates n=13-30; biological replicates n=3-6. In **A-O, R-S** data are shown as mean ± SEM. *P* values were calculated in **A-O** using one-way ANOVA, in **R-S** using Unpaired t-test. $*p < 0.05$; $**p < 0.01$; $***p < 0.001$; $****p < 0.0001$.

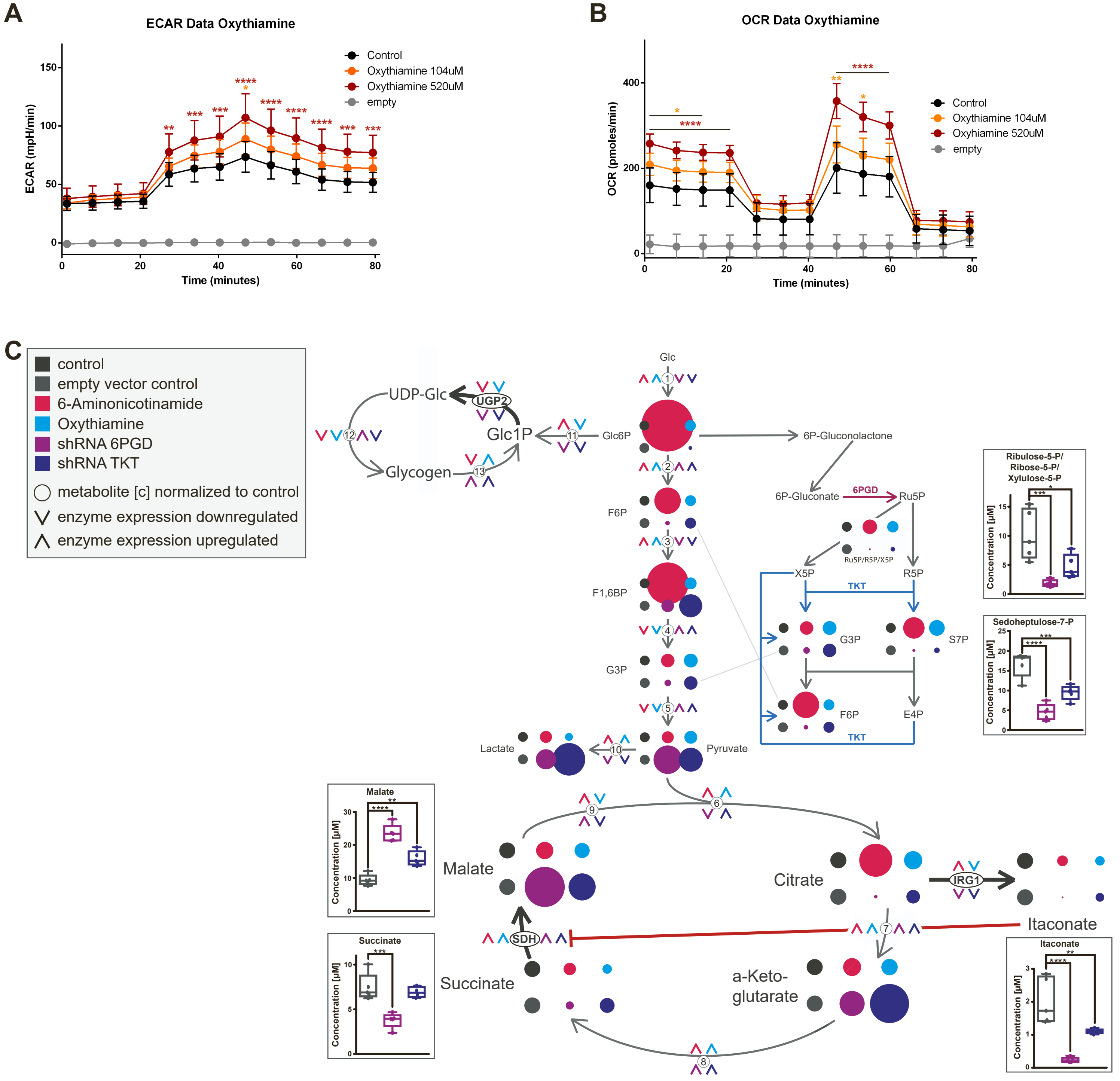
A
ECAR Data Oxythiamine
ECAR (mpH/min)
Time (minutes)
Control
Oxythiamine 104uM
Oxythiamine 520uM
empty
B
OCR Data Oxythiamine
OCR (pmoles/min)
Time (minutes)
Control
Oxythiamine 104uM
Oxyhiamine 520uM
empty
C
control
empty vector control
6-Aminonicotinamide
Oxythiamine
shRNA 6PGD
shRNA TKT
metabolite [c] normalized to control
enzyme expression downregulated
enzyme expression upregulated
UDP-Glc
UGP2
Glc1P
Glycogen
Glc
Glc6P
6P-Gluconolactone
6P-Gluconate
6PGD
Ru5P
Ru5P/R5P/X5P
X5P
R5P
TKT
G3P
S7P
F6P
E4P
F1,6BP
Lactate
Pyruvate
Malate
Citrate
IRG1
Itaconate
SDH
Succinate
a-Keto-
glutarate
Ribulose-5-P/
Ribose-5-P/
Xylulose-5-P
Sedoheptulose-7-P
Concentration [µM]

**Figure S4. PPP modulation changes metabolic activity in macrophages.**

**Related to Figures 3 and 5.**

**(A-B)** Measurement of metabolic activity of J774A.1 macrophages under drug mediated PPP inhibition with oxythiamine by SeaHorse analysis. **A** one representative example of MitoStress test measurement of ECAR, **B** one representative example of MitoStress test measurement of OCR. **C** Metabolomic analysis of central metabolic pathways with overlay of proteomics data under compound mediated PPP inhibition compared to untreated J774A.1 macrophages and shRNA mediated PPP knockdown of 6Pgd and Tkt compared to empty vector control J774A.1 macrophages. Relative metabolite abundance compared to respective control represented in circle size. Absolute amount of metabolites of interest displayed in bar graphs. Change in enzyme expression assessed by proteomics displayed in arrow direction. Arrow upwards: increased enzyme expression compared to respective control; arrow downwards: decreased enzyme expression compared to respective control. Inhibited enzyme reactions by compounds or shRNA mediated knockdown coloured in violet (6Pgd) and blue (Tkt). *Metabolites: E4P* erythrose-4-phosphate, *F1,6BP* fructose-1,6-bisphosphate, *F6P* fructose-6-phosphate, *G3P* glyceraldehyde-3-phosphate, *Glc* glucose, *Glc1P g*lucose-1-phosphate, *Glc6P* glucose-6-phosphate, *R5P* ribose-5-phosphate, *Ru5P* ribulose-5-phosphate, *S7P* sedoheptulose-7-phosphate, *UDP-Glc* UDP-glucose, *X5P* xylulose-5-phosphate. *Enzymes: Irg1* immune-regulatory gene 1, *Sdh* succinate dehydrogenase, *Ugp2* UDP-glucose pyrophosphorylase 2, *1)* hexokinase, *2)* glucose-6-phosphate isomerase, *3)* phosphofructokinase, *4)* aldolase, *5)* sum up of glycerinaldehyde-3-phosphate dehydrogenase, phosphoglycerate kinase, enolase, pyruvate kinase, *6)* citrate synthase, *7)* sum up of aconitase, isocitrate dehydrogenase, *8)* sum up of a-ketoglutarate dehydrogenase, succinyl-CoA synthetase, *9)* malate dehydrogenase, *10)* lactate dehydrogenase, *11)* phosphoglucomutase 1, *12)* UTP-glucose-1-phosphate uridyltransferase, *13)* glycogen phosphorylase.

Technical replicates **A**-**B** n=6-27, **C** n=3; biological replicates **A**-**B** n=1-9, **C** n=3. In **A-B** data are shown as mean of six replicates in one experiment ± SD, n=6, in **C** data are shown as Min. to Max, n=3. *P* values were calculated using one-way ANOVA. *$p < 0.05$; **$p < 0.01$; ***$p < 0.001$; ****$p < 0.0001$.

A

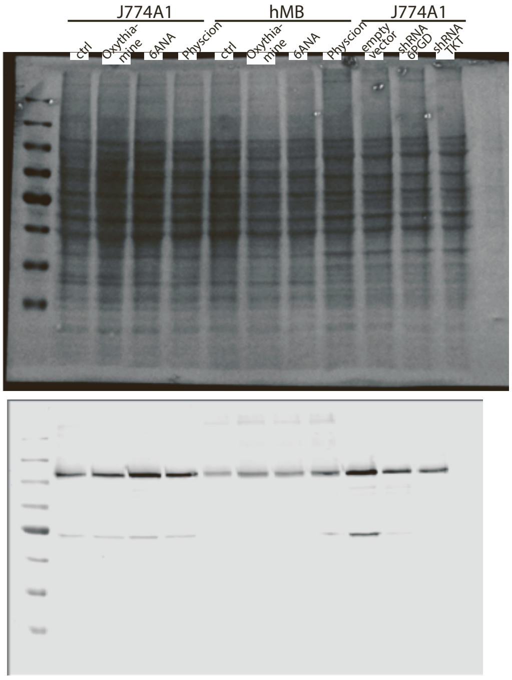
J774A1
hMB
J774A1
ctrl
Oxythia-mine
6ANA
Physcion
ctrl
Oxythia-mine
6ANA
Physcion
empty vector
shRNA 6PGD
shRNA TKT

B

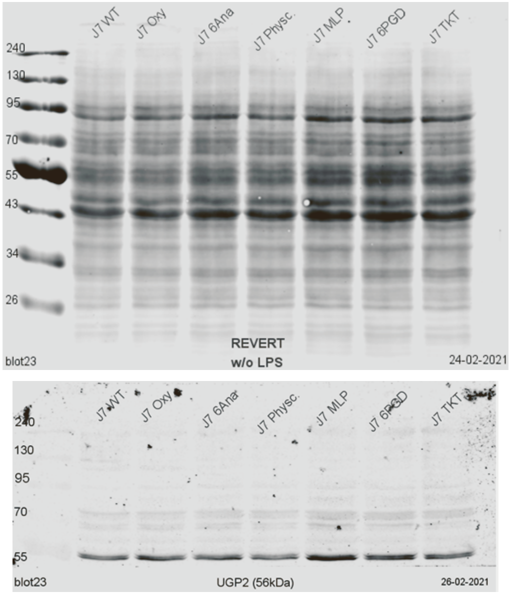
J7 WT
J7 Oxy
J7 6Ana
J7 Physc.
J7 MLP
J7 6PGD
J7 TKT
240
130
95
70
55
43
34
26
blot23
REVERT
w/o LPS
24-02-2021
UGP2 (56kDa)
26-02-2021

C

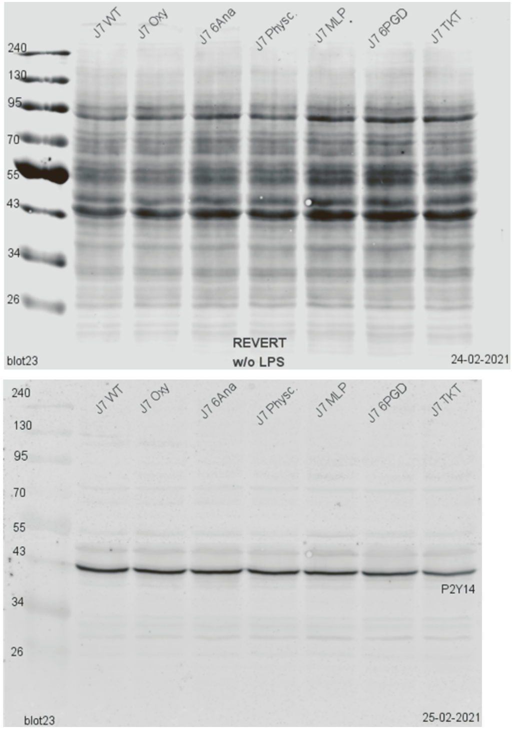
J7 WT
J7 Oxy
J7 6Ana
J7 Physc.
J7 MLP
J7 6PGD
J7 TKT
240
130
95
70
55
43
34
26
blot23
REVERT
w/o LPS
24-02-2021
P2Y14
25-02-2021

D

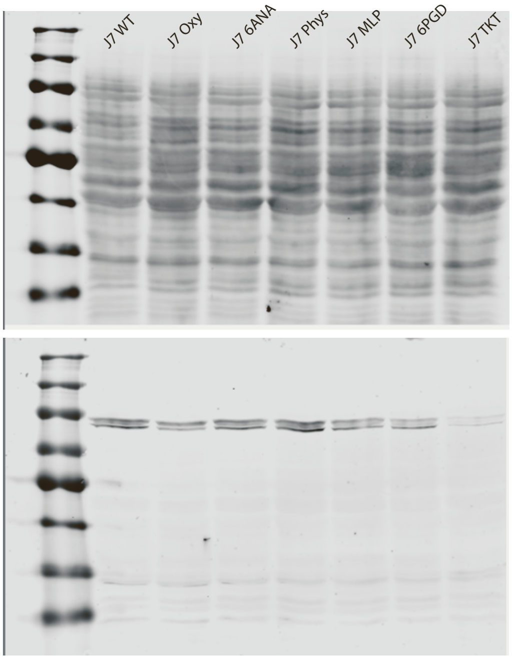
J7 WT
J7 Oxy
J7 6ANA
J7 Phys
J7 MLP
J7 6PGD
J7 TKT

E

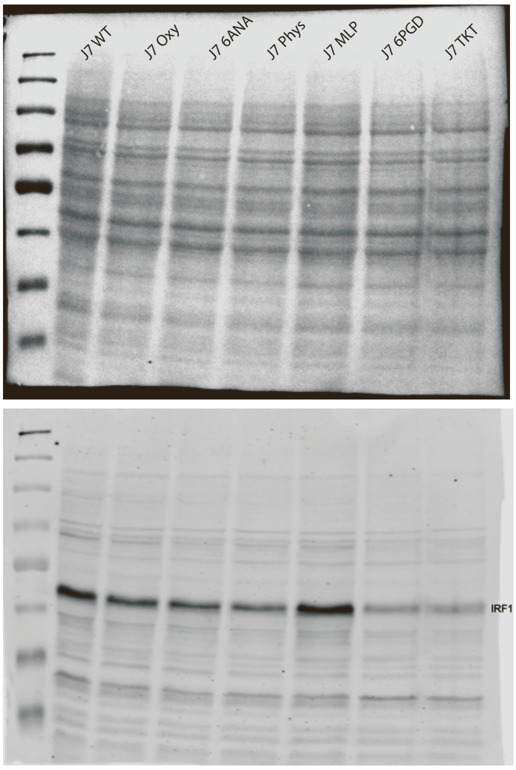
J7 WT
J7 Oxy
J7 6ANA
J7 Phys
J7 MLP
J7 6PGD
J7 TKT
IRF1

F

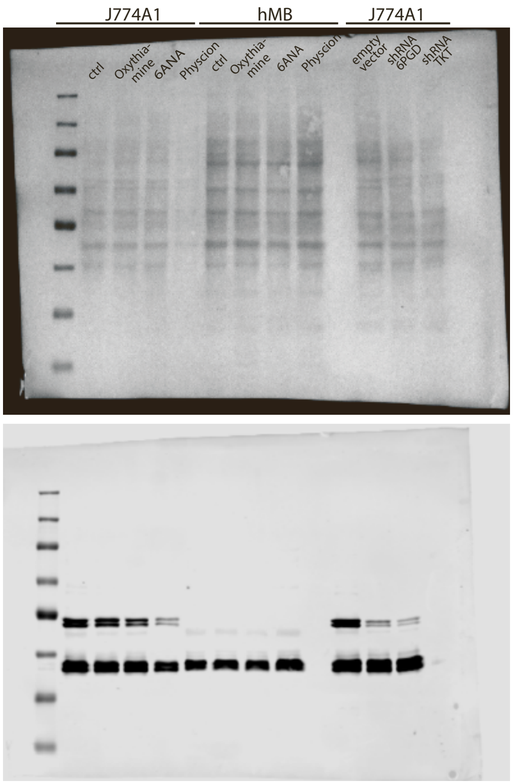
J774A1
hMB
J774A1
ctrl
Oxythia-mine
6ANA
Physcion
ctrl
Oxythia-mine
6ANA
Physcion
empty vector
shRNA 6PGD
shRNA TKT

**Figure S5. PPP inhibition changes the protein expression of hypothesized metabolic-immune response axis in macrophages.**

**Related to Figures 4 and 5.**

**(A-F)** One representative example of western blot analysis of J744A.1 macrophages after drug mediated inhibition of the PPP or shRNA mediated knockdown of the PPP. Total protein stain and staining of protein of interest are shown. In **A** and **F** also hMB cells under PPP inhibition has been tested. **A** PYK2 staining, **B** UGP2 staining, **C** P2Y14 staining, **D** STAT1 staining, **E** IRF1 staining, **F** IRG1 staining.

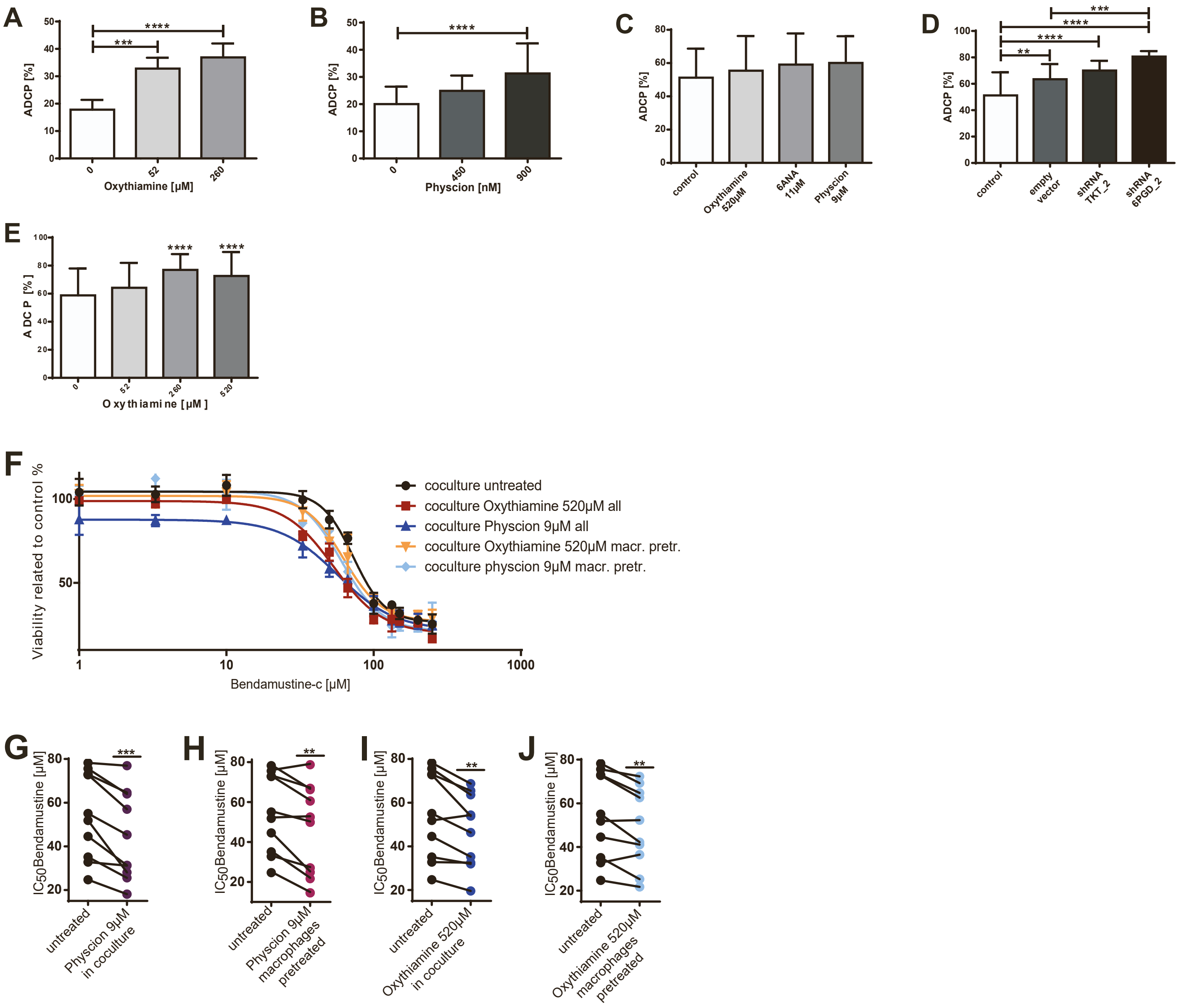
A
ADCP [%]
Oxythiamine [µM]
0
52
260
***
****
B
ADCP [%]
Physcion [nM]
0
450
900
****
C
ADCP [%]
control
Oxythiamine 520µM
6ANA 11µM
Physcion 9µM
D
ADCP [%]
control
empty vector
shRNA TKT_2
shRNA 6PGD_2
**
****
****
***
E
ADCP [%]
Oxythiamine [µM]
0
52
260
520
****
****
F
Viability related to control %
Bendamustine-c [µM]
coculture untreated
coculture Oxythiamine 520µM all
coculture Physcion 9µM all
coculture Oxythiamine 520µM macr. pretr.
coculture physcion 9µM macr. pretr.
G
IC50Bendamustine [µM]
untreated
Physcion 9µM in coculture
***
H
IC50Bendamustine [µM]
untreated
Physcion 9µM macrophages pretreated
**
I
IC50Bendamustine [µM]
untreated
Oxythiamine 520µM in coculture
**
J
IC50Bendamustine [µM]
untreated
Oxythiamine 520µM macrophages pretreated
**

**Figure S6. PPP inhibition in primary human environment increases phagocytic capacity of macrophages and favours primary CLL cells chemotherapy sensitivity.**

**Related to Figure 6.**

**(A-B)** ADCP rate of primary human monocyte derived macrophages differentiated in the presence of PPP inhibitors. **A** ADCP change by primary monocyte derived macrophages differentiated in the presence of physcion and M-CSF **B** ADCP change by primary monocyte derived macrophages differentiated in the presence of oxythiamine and M-CSF. **(C-D)** ADCP rate of primary CLL patient cells by J774A.1 macrophages. **C** ADCP rate under drug mediated PPP inhibition, **D** ADCP rate under shRNA mediated PPP knockdown. **(E)** ADCP rate of primary CLL patient cells by primary human monocyte derived macrophages differentiated in the presence of oxythiamine and M-CSF. **(F)** One representative example of dose response curve of individual orimary CLL patient cell samples towards bendamustine treatment. Cells were incubated with bendamustine after protective macrophage co-culture with untreated J774A.1 macrophages vs. PPP inhibition. **(G-J)** Dose-response curve ($IC_{50}$) for individual primary CLL patient cell samples to bendamustine treatment. Cells were incubated with bendamustine after protective macrophage co-culture with untreated J774A.1 macrophages vs. PPP inhibition. **G-H** Inhibition of 6Pgd in oxidative part of PPP by physcion, **G** co-culture treatment, **H** macrophage pre-treatment. **I-J** Inhibition of Tkt in non-oxidative part of PPP by oxythiamine, **I** co-culture treatment, **J** macrophage pre-treatment.

Technical replicates **A** n=28, **B** n=20, **C-D** n=20, **E** n=65, **F** n=30, **G-J** n=30; biological replicates **A** n=6, **B** n=4, **C-D** n=5, **E** n=12, **F** n=10, **G-J** n=10. Data are shown as mean ± SEM. *P* values were calculated in **A-E** by using one-way ANOVA, in **G-J** using paired t-test. *$p < 0.05$; **$p < 0.01$; ***$p < 0.001$; ****$p < 0.0001$.

**A**

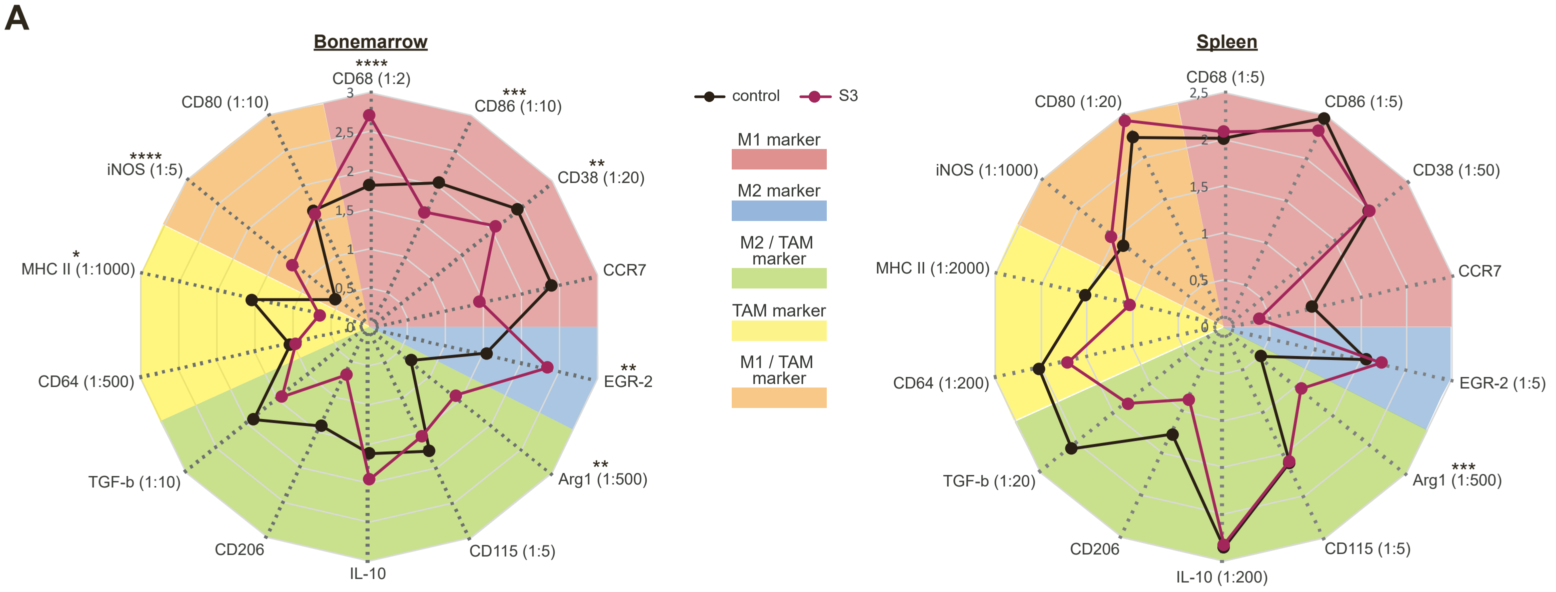


**B**

**Benjamini-Hochberg-analysis**

| | vehicle | alemtuzumab | S3 + alemtuzumab |
|---|---|---|---|
| vehicle | -- | **1.4e-05** | -- |
| S3 | **0.6159** | **5.5e-05** | **1.0e-07** |
| S3 + alemtuzumab | **3.4e-09** | **0.0059** | -- |

**C**

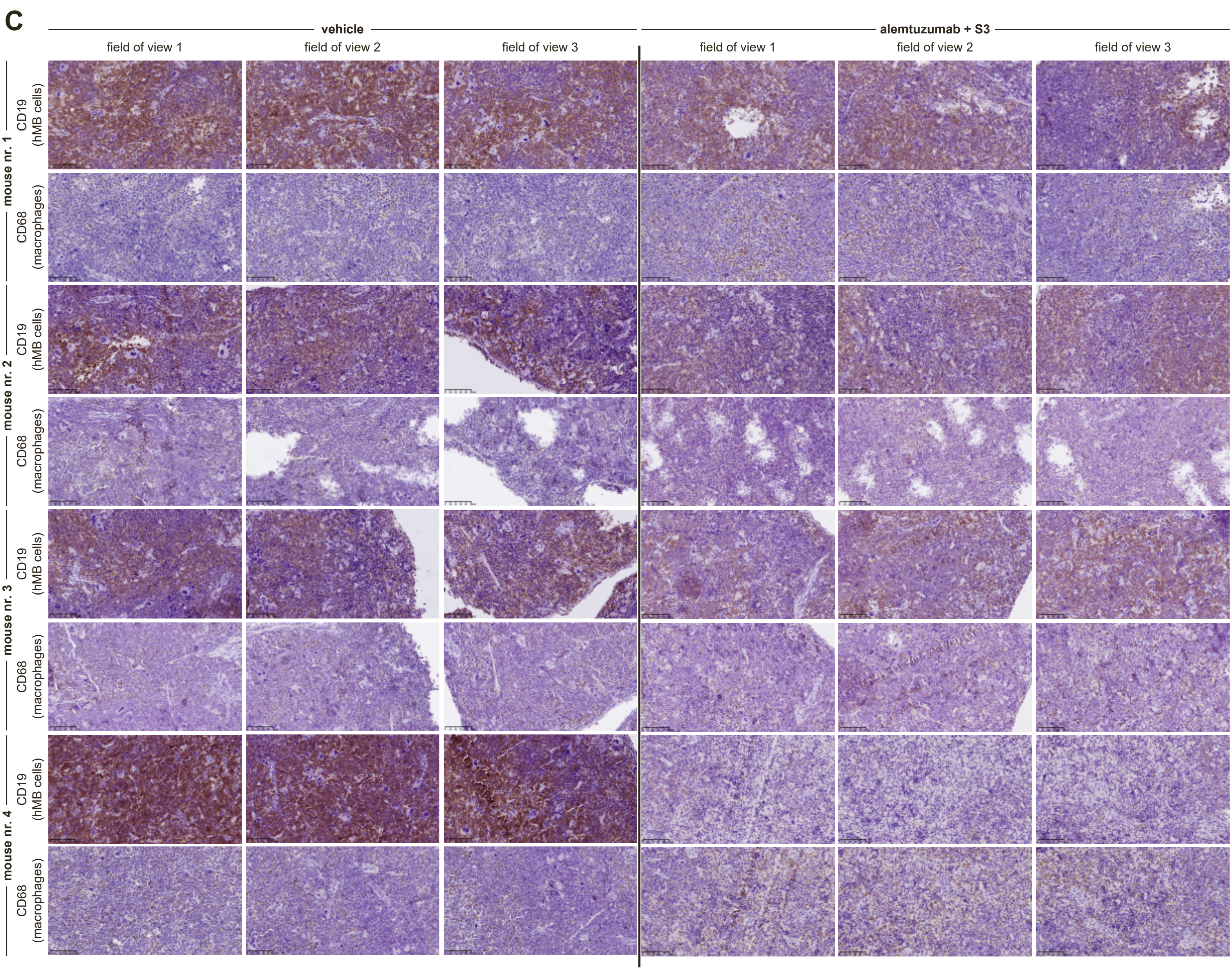

**Figure S7. PPP inhibition increases myelopoiesis and macrophages´ activity *in vivo* and improves treatment response in an aggressive humanized lymphoma mouse model.**

**Related to Figure 7.**

**(A)** Expression of characteristic surface marker for different macrophage subtypes on macrophages in bone marrow and spleen. C57BL/6 mice treated with vehicle (control) or S3 i.p. for 7 days. **(B)** Significance testing by using Benjamini-Hochberg-analysis of survival curves of NSG mice transfected with hMB and treated after three days of engraftment with vehicle, alemtuzumab and/or PPP inhibitor S3 for 12 days. **(C)** Immunohistochemical staining of hMB cells ($CD19^+$) and macrophages ($CD68^+$) in spleen of NSG mice transfected with hMB and treated after three days of engraftment with vehicle or alemtuzumab + S3 for 12 days.

Technical replicates **A** n=9-10, **B** n=21-25, **C** n=4; biological replicates **A** n=9-10, **B** n=21-25, **C** n=4. In **A** data are shown as mean of ten replicates. In **A** *P* values were calculated by using one-way ANOVA. $*p < 0.05$; $**p < 0.01$; $***p < 0.001$; $****p < 0.0001$.

**Table S6. Qualifier and quantifier transition of metabolites measured by targeted LC-QqQ/MS analysis. Related to Figure 5.**

| Compound name | Transition (m/z) | Transition type | Fragmentor (V) | Collision Energy (eV) | Cell accelerator (V) | RT (min) |
|---|---|---|---|---|---|---|
| *Succinic acid D6 (ISTD)* | 121 → 77 | quantifier | 77 | 10 | 4 | 11.9 |
| *Glutathione - glycine-$^{13}C_2$,$^{15}N$ trifluor (ISTD)* | 309 → 146 | quantifier | 119 | 14 | 4 | 12.3 |
| | 309 → 128 | qualifier | 119 | 14 | 4 | 12.3 |
| *Adenosine 5-diphosphate* | 426 → 328 | quantifier | 56 | 16 | 4 | 17.9 |
| | 426 → 159 | qualifier | 56 | 28 | 4 | 17.9 |
| *Adenosine 5-triphosphate* | 506 → 408 | quantifier | 122 | 22 | 4 | 20.5 |
| | 506 → 159 | qualifier | 122 | 38 | 4 | 20.5 |
| *Lactic acid* | 89 → 45 | qualifier | 48 | 9 | 4 | 3.0 |
| | 89 → 43 | quantifier | 48 | 10 | 4 | 3.0 |
| *Pyruvic Acid* | 87 → 43 | quantifier | 48 | 4 | 4 | 2.2 |
| *alpha - ketoglutaric acid* | 145 → 101 | quantifier | 70 | 5 | 4 | 10.6 |
| | 145 → 57 | qualifier | 70 | 9 | 4 | 10.6 |
| *NAD* | 662 → 540 | quantifier | 70 | 12 | 4 | 14.4 |
| | 662 → 328 | qualifier | 70 | 36 | 4 | 14.4 |
| *D-Fructose 6-phosphate* | 259 → 97 | quantifier | 102 | 14 | 4 | 16.3 |
| | 259 → 79 | qualifier | 102 | 48 | 4 | 16.3 |
| *D-Glucose-6-phosphate* | 259 → 79 | quantifier | 102 | 48 | 4 | 18.5 |
| | 259 → 97 | qualifier | 102 | 14 | 4 | 18.5 |
| *Ribose 5-phosphate / Ribulose 5-phosphate* | 229 → 97 | qualifier | 96 | 10 | 4 | 14.3 |
| | 229 →79 | quantifier | 96 | 48 | 4 | 14.3 |
| *D-Xylulose 5-phosphate / Ribose 5-phosphate / Ribulose 5-phosphate* | 229 → 139 | qualifier | 86 | 8 | 4 | 15.3 |
| | 229 → 79 | quantifier | 96 | 48 | 4 | 15.3 |
| *D-Sedoheptulose 7-phosphate* | 289 → 97 | quantifier | 104 | 18 | 4 | 17.0 |
| | 289 → 79 | qualifier | 104 | 48 | 4 | 17.0 |
| *Glutathione Reduced* | 306 → 143 | quantifier | 109 | 14 | 4 | 12.3 |
| | 306 → 128 | qualifier | 109 | 14 | 4 | 12.3 |
| *NADP* | 742 → 620 | quantifier | 79 | 10 | 4 | 24.9 |
| | 742 → 408 | qualifier | 79 | 38 | 4 | 24.9 |
| *Succinic acid* | 117 → 99 | qualifier | 62 | 8 | 4 | 11.9 |
| | 117 → 73 | quantifier | 62 | 10 | | 11.9 |
| *Itaconic acid* | 129 → 85 | quantifier | 68 | 6 | 4 | 8.4 |
| | 129 → 41 | qualifier | 68 | 12 | 4 | 8.4 |
| L-Malic acid | 133 → 115 | quantifier | 76 | 8 | 4 | 12.8 |
| | 133 → 71.1 | qualifier | 76 | 14 | 4 | 12.8 |
| *Citric acid* | 191 → 111 | quantifier | 78 | 10 | 4 | 17.5 |
| | 191 → 87 | qualifier | 78 | 16 | 4 | 17.5 |

| | | | | | | |
|---|---|---|---|---|---|---|
| *NADH* | 664 → 408 | qualifier | 190 | 29 | 4 | 11.7 |
| | 664 → 79 | quantifier | 190 | 49 | 4 | 11.7 |
| *DL-Glyceraldehyde 3-phosphate* | 169 → 97 | quantifier | 130 | 4 | 4 | 14.7 |
| | 169 → 79 | qualifier | 130 | 28 | 4 | 14.7 |
| *D-Fructose 1,6-biphosphate* | 339 → 241 | qualifier | 90 | 12 | 4 | 25.9 |
| | 339 → 97 | quantifier | 90 | 22 | 4 | 25.9 |
| *6-phosphogluconic acid* | 275 → 79 | quantifier | 109 | 49 | 4 | 21.7 |
| *D-erytrose 4-phosphate* | 199 → 97 | quantifier | 151 | 6 | 4 | 13.9 |
| | 199 → 79 | qualifier | 151 | 30 | 4 | 13.9 |
