# Supplementary material for: Macrophages are activated toward phagocytic lymphoma cell clearance by pentose phosphate pathway inhibition
Source: Cell Rep Med. 2024 Nov 26;5(12):101830. doi: 10.1016/j.xcrm.2024.101830 (PMC11722127; doi:10.1016/j.xcrm.2024.101830)
Supplement: Document S2. Article plus supplemental information [file mmc3.pdf]

Cell Reports Medicine

Article

Macrophages are activated toward phagocytic lymphoma cell clearance by pentose phosphate pathway inhibition

Graphical abstract

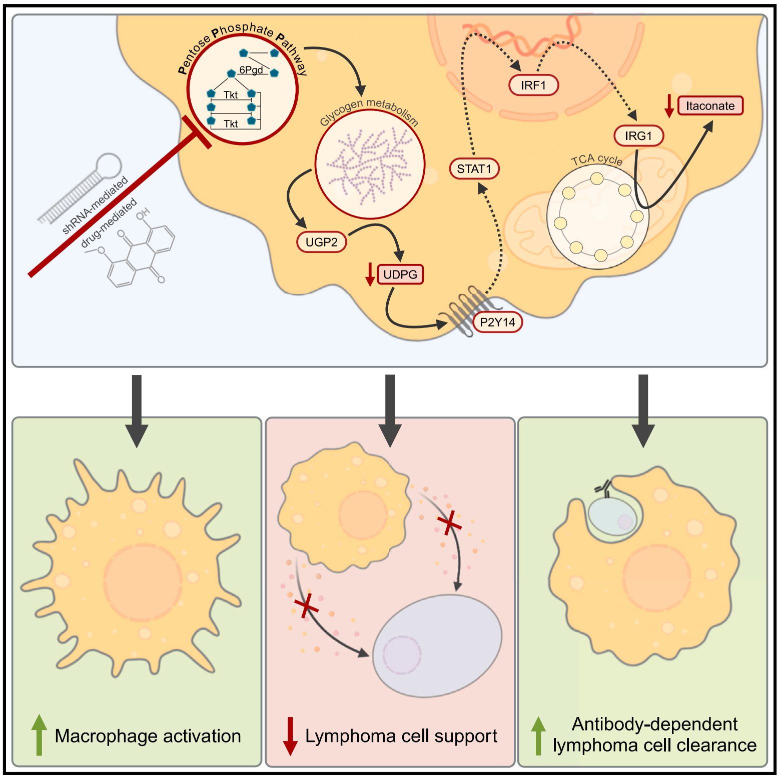

Highlights

- Macrophage-mediated lymphoma cell phagocytosis is increased by PPP inhibition
- PPP inhibition is an immune-regulatory switch for macrophage function
- PPP inhibition is linked to the modulation of the UDPG-Stat1-Irg1-itaconate axis
- PPP inhibition is tolerable *in vivo* and boosts therapeutic B cell lymphoma targeting

Authors

Anna C. Beielstein, Elena Izquierdo, Stuart Blakemore, ..., Marcus Krüger, Coral Barbas, Christian P. Pallasch

Correspondence

christian.pallasch@uk-koeln.de

In brief

Beielstein et al. have shown that the metabolic inhibition of the pentose phosphate pathway in macrophages leads to increased macrophage activation, less lymphoma cell support, and increased antibody-dependent lymphoma cell clearance by macrophages. A significantly improved overall survival in a therapeutic lymphoma mouse model is achieved.

Beielstein et al., 2024, Cell Reports Medicine *5*, 101830
December 17, 2024 © 2024 The Author(s). Published by Elsevier Inc.
https://doi.org/10.1016/j.xcrm.2024.101830

CellPress

Cell Reports Medicine

CellPress
OPEN ACCESS

Article

Macrophages are activated toward phagocytic lymphoma cell clearance by pentose phosphate pathway inhibition

Anna C. Beielstein,[1,2] Elena Izquierdo,[1,2,3] Stuart Blakemore,[1,2] Nadine Nickel,[1,2] Michael Michalik,[2] Samruddhi Chawan,[1,2] Reinhild Brinker,[2] Hans-Henrik Bartel,[1,2] Daniela Vorholt,[1,2] Lukas Albert,[1,2] Janica L. Nolte,[2] Rebecca Linke,[1,2] Carolina Raíssa Costa Picossi,[4] Jorge Sáiz,[4] Felix Picard,[5,6] Alexandra Florin,[7] Jörn Meinel,[7] Reinhard Büttner,[7] Paul Diefenhardt,[6,8] Sebastian Brähler,[6,8] Alma Villaseñor,[4] Holger Winkels,[5,6] Michael Hallek,[1,2,6] Marcus Krüger,[1,2,6] Coral Barbas,[4] and Christian P. Pallasch[1,2,6,9,*]

[1]Department I of Internal Medicine, Centre for Integrated Oncology (CIO) Aachen-Bonn-Cologne-Duesseldorf, University Hospital Cologne, 50937 Cologne, Germany
[2]Cologne Excellence Cluster for Cellular Stress Responses in Ageing-Associated Diseases (CECAD), University of Cologne, 50931 Cologne, Germany
[3]Departamento de Ciencias Médicas Básicas, Facultad de Medicina, Instituto de Medicina Molecular Aplicada – Nemesio Díez (IMMA-ND), Universidad San Pablo-CEU, CEU Universities, Urbanización Montepríncipe, 28668 Boadilla del Monte, Spain
[4]Centro de Metabolómica y Bioanálisis (CEMBIO), Facultad de Farmacia, Universidad San Pablo-CEU, CEU Universities, Urbanización Montepríncipe, 28668 Boadilla del Monte, Spain
[5]Department III of Internal Medicine, Faculty of Medicine and University Hospital Cologne, University of Cologne, 50937 Cologne, Germany
[6]Centre for Molecular Medicine Cologne (CMMC), University of Cologne, 50937 Cologne, Germany
[7]Institute of Pathology, Faculty of Medicine and University Hospital Cologne, University of Cologne, 50937 Cologne, Germany
[8]Department II of Internal Medicine, Faculty of Medicine and University Hospital Cologne, University of Cologne, 50937 Cologne, Germany
[9]Lead contact
*Correspondence: christian.pallasch@uk-koeln.de
https://doi.org/10.1016/j.xcrm.2024.101830

SUMMARY

Macrophages in the B cell lymphoma microenvironment represent a functional node in progression and therapeutic response. We assessed metabolic regulation of macrophages in the context of therapeutic antibody-mediated phagocytosis. Pentose phosphate pathway (PPP) inhibition induces increased phagocytic lymphoma cell clearance by macrophages *in vitro*, in primary human chronic lymphocytic leukemia (CLL) patient co-cultures, and in mouse models. Addition of the PPP inhibitor S3 to antibody therapy achieves significantly prolonged overall survival in an aggressive B cell lymphoma mouse model. PPP inhibition induces metabolic activation and pro-inflammatory polarization of macrophages while it decreases macrophages' support for survival of lymphoma cells empowering anti-lymphoma function. As a mechanism of macrophage repolarization, the link between PPP and immune regulation was identified. PPP inhibition causes decreased glycogen level and subsequent modulation of the immune modulatory uridine diphosphate glucose (UDPG)-Stat1-Irg1-itaconate axis. Thus, we hypothesize the PPP as a key regulator and targetable modulator of macrophage activity in lymphoma to improve efficacy of immunotherapies and prolong survival.

INTRODUCTION

The tumor microenvironment (TME) represents a hallmark of cancer, and interactions between its transformed and non-transformed immune bystander cells determine disease progression and therapeutic response.[1] Tumor cells generate a tumor-supportive environment by cytokine and metabolite secretion. These mediators alter occurrence of bystander cells and shift the activity of the infiltrating immune cells from an anti- to a pro-tumoral response.[2] Tumor-associated macrophages (TAMs) play a critical role in promoting tumor growth, facilitating vascularization and metastasis and suppressing other immune cells.[3,4]

However, we have shown that macrophages are central for tumor cell clearance in aggressive B cell lymphoma during immunotherapy, although their phagocytic capacity becomes impaired by lymphoma cells.[5]

Recent decades have seen the development of numerous new treatment strategies for B cell malignancies, which have extended patient survival but struggled to substantially increase cure rates. The front-line strategy is chemo-immunotherapy, combining therapeutic antibodies like rituximab or obinutuzumab with chemotherapy. We demonstrated that leukemia cells in therapy-refractory niches reduce the engulfment of antibody-targeted tumor cells by macrophages, diminishing therapy efficacy and leading to relapse.[5]

© 2024 The Author(s). Published by Elsevier Inc.
This is an open access article under the CC BY license (http://creativecommons.org/licenses/by/4.0/).

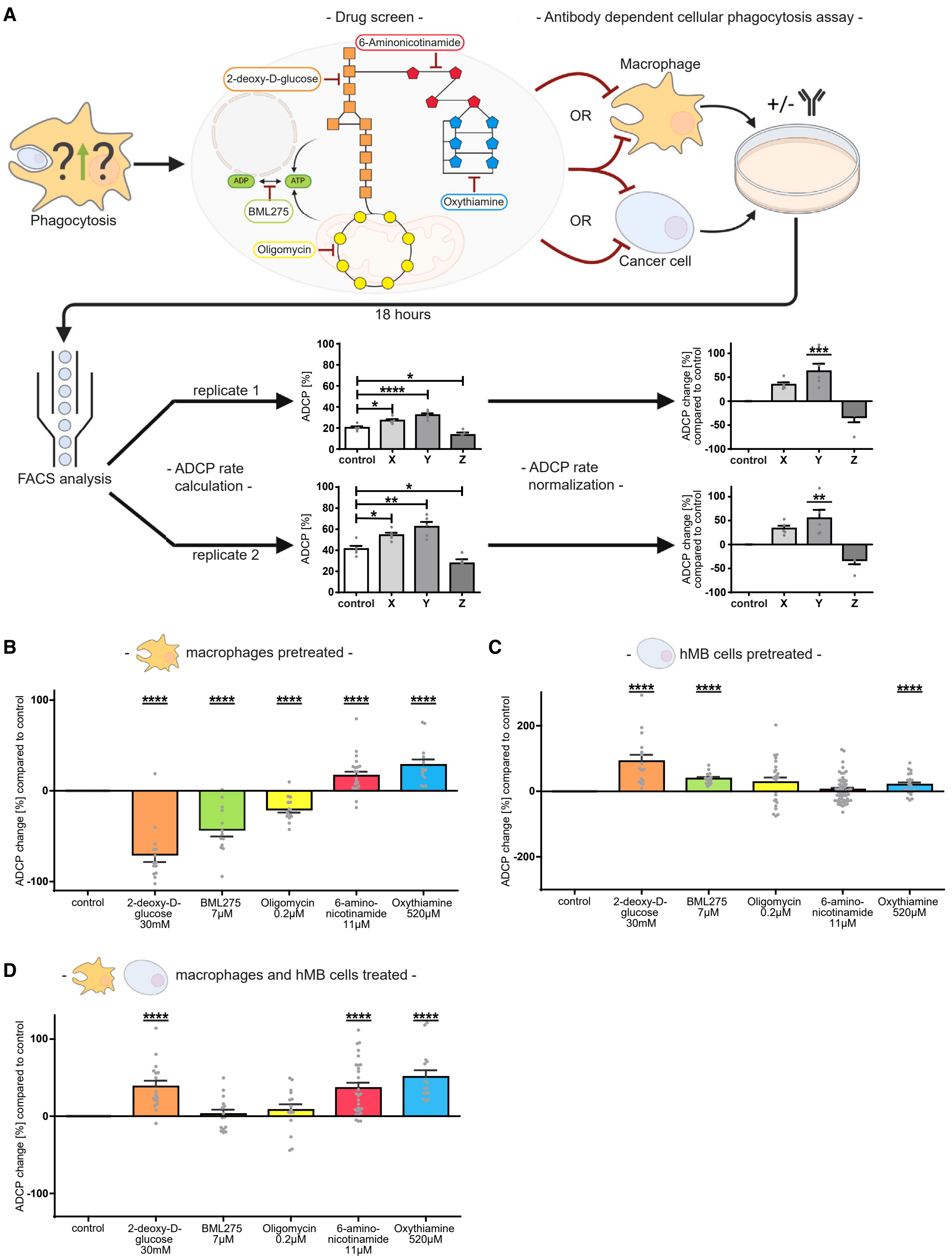

(legend on next page)

CellPress
OPEN ACCESS

Macrophage function depends on their local environment, which impacts their differentiation and polarization. Macrophages are a heterogeneous population with different subtypes exerting pro-inflammatory and phagocytic (M1-like macrophages) and anti-inflammatory and tissue-regenerative (M2-like macrophages) activities. TAMs represent a blend of these characteristics tending toward the anti-inflammatory and phagocytic inactive phenotype.[6,7] The TME includes activation mediators such as cytokines, chemokines, and metabolites, which control the polarization of contained macrophages. Changes in the microenvironment can alter macrophage metabolism, which is crucial for polarization and the closely linked macrophage function.[8,9] Changes in the cellular metabolism have the ability to repolarize the macrophage phenotype by which pro-tumoral TAMs could acquire anti-tumoral activity.[10–12]

The interaction between macrophages and lymphoma cells, as well as macrophages metabolic sensitivity, opens up a promising strategy to optimize anti-cancer therapy. Modulating macrophage metabolism may improve their anti-tumor efficacy and diminish their tumor-supportive function.

In the present study, we demonstrate that pentose phosphate pathway (PPP) inhibition in macrophages increases their activity and phagocytic capacity whereby pro-tumoral bystander function is diminished. As a driving mechanism, we discovered a connection between metabolism and immune regulation by modulation of the UDPG-Stat1-Irg1-itaconate axis. The effects of PPP inhibition were transmitted into human patient samples and also reproduced *in vivo*, where significantly increased survival in an aggressive lymphoma mouse model was achieved. These results open up a promising field of treatment strategy against B cell malignancies in clinical use.

RESULTS

Metabolic inhibition of the PPP leads to increased phagocytic capacity of macrophages

To investigate how metabolic modulation of TAMs in the context of immunotherapy affects phagocytic capacity, we performed a metabolism-focused screening approach for antibody-dependent cellular phagocytosis (ADCP). Key metabolic pathways were blocked using representative inhibitors in a macrophage and humanized aggressive B cell lymphoma (hMB; cell line information see STAR Methods) co-culture-assay system, and phagocytosis was assessed through specific antibody targeting (alemtuzumab; anti-CD52) (Figure 1A). The antibody alemtuzumab was used as a tool compound for the first screening approach as several types of lymphoma downregulate CD20 expression but not CD52 expression, also seen in hMB cells. Several other antibodies, currently in clinical use, were investigated in further analysis.

Inhibition of glycolysis (via 2-deoxy-D-glucose), AMP-activated protein kinase (AMPK)-mediated cell energy regulation (via BML-275), mitochondrial ATP production (via oligomycin), and the PPP (via 6-aminonicotinamide and oxythiamine) was screened using non-toxic inhibitor concentrations (Figure S1). The inhibition was conducted in co-culture and by pre-treatment of each cell type (macrophage or hMB cell), to infer specific macrophage vs. lymphoma cell phagocytic interactions. As the basal phagocytosis rate of macrophages is variable, the change in phagocytosis under treatment was calculated in comparison to the basal phagocytosis rate (=ADCP change, Figures 1A and S2).

Glycolysis inhibition significantly increased ADCP rates in co-culture (+40%, $p < 0.01$) and by pre-treatment of lymphoma cells (+95%, $p < 0.0001$), while macrophage pre-treatment significantly diminished ADCP rate (−71%, $p < 0.001$) (Figures 1B–1D). Similarly, AMPK inhibition increased ADCP rate significantly by lymphoma cell pre-treatment (+39%, $p < 0.0001$) and significantly diminished ADCP rate by pre-treatment of macrophages (−42%, $p < 0.001$) (Figures 1C and 1D). Inhibition of mitochondrial ATP production also diminished ADCP rate significantly by macrophage pre-treatment (−21%, $p < 0.0001$) (Figure 1D).

Sole inhibition of the PPP induced significantly increased ADCP rates by co-culture treatment and macrophage pre-treatment. The increase was induced by both inhibition of the oxidative part of the PPP via 6-phosphogluconate dehydrogenase inhibition (6Pgd; inhibitor 6-aminonicotinamide) (co-culture +40% $p < 0.01$; macrophage pre-treatment +15%, $p < 0.05$) and inhibition of the non-oxidative part via transketolase inhibition (Tkt; inhibitor oxythiamine) (co-culture +51% $p < 0.001$; macrophage pre-treatment +28%, $p < 0.01$) (Figures 1B and 1D). Moreover, lymphoma cell pre-treatment with oxythiamine increased phagocytic rate significantly (+19%, $p < 0.0001$) (Figure 1C).

Of note, Tkt inhibition induced the highest increase in phagocytic capacity in the co-culture and by pre-treatment of macrophages in the screening approach.

Thus, inhibition of glycolysis, AMPK, and mitochondrial ATP production negatively affected macrophages' phagocytic capacity, while blocking PPP favored lymphoma cell clearance by macrophages.

Cross validation of PPP inhibition in macrophages confirms increased ADCP rates

To further investigate the PPP in the context of macrophages' function and as a target for improving immunotherapy, we applied alternative inhibitors (6Pgd: physcion, Tkt: *p*-hydroxyphenylpyruvate)[13,14] and confirmed significant increases in ADCP rates (physcion +26%, $p < 0.0001$; *p*-hydroxyphenylpyruvate +25%, $p < 0.001$) (Figure 2A). Additionally, we recapitulated the phagocytosis assays with the human monocyte cell line THP1 using an alternative antibody (obinutuzumab; anti-CD20

Figure 1. Metabolic inhibition of the pentose phosphate pathway leads to increased phagocytic rate of macrophages
(A) Scheme of ADCP-based metabolic screening approach.
(B–D) Summary of ADCP change compared to basal phagocytosis rate of J774A.1 macrophages under inhibition of respective metabolic pathways. (B) Inhibition of only macrophages. (C) Inhibition of only hMB cells. (D) Inhibition of all co-culture components. Technical replicates (B) $n = 15–22$, (C) $n = 15–58$, (D) $n = 15–28$; biological replicates (B) $n = 3–5$, (C) $n = 3–12$, (D) $n = 3–6$. Data are shown as mean ± SEM. *p* values were calculated using unpaired t test. *$p < 0.05$; **$p < 0.01$; ***$p < 0.001$; ****$p < 0.0001$. See also Figures S1 and S2.

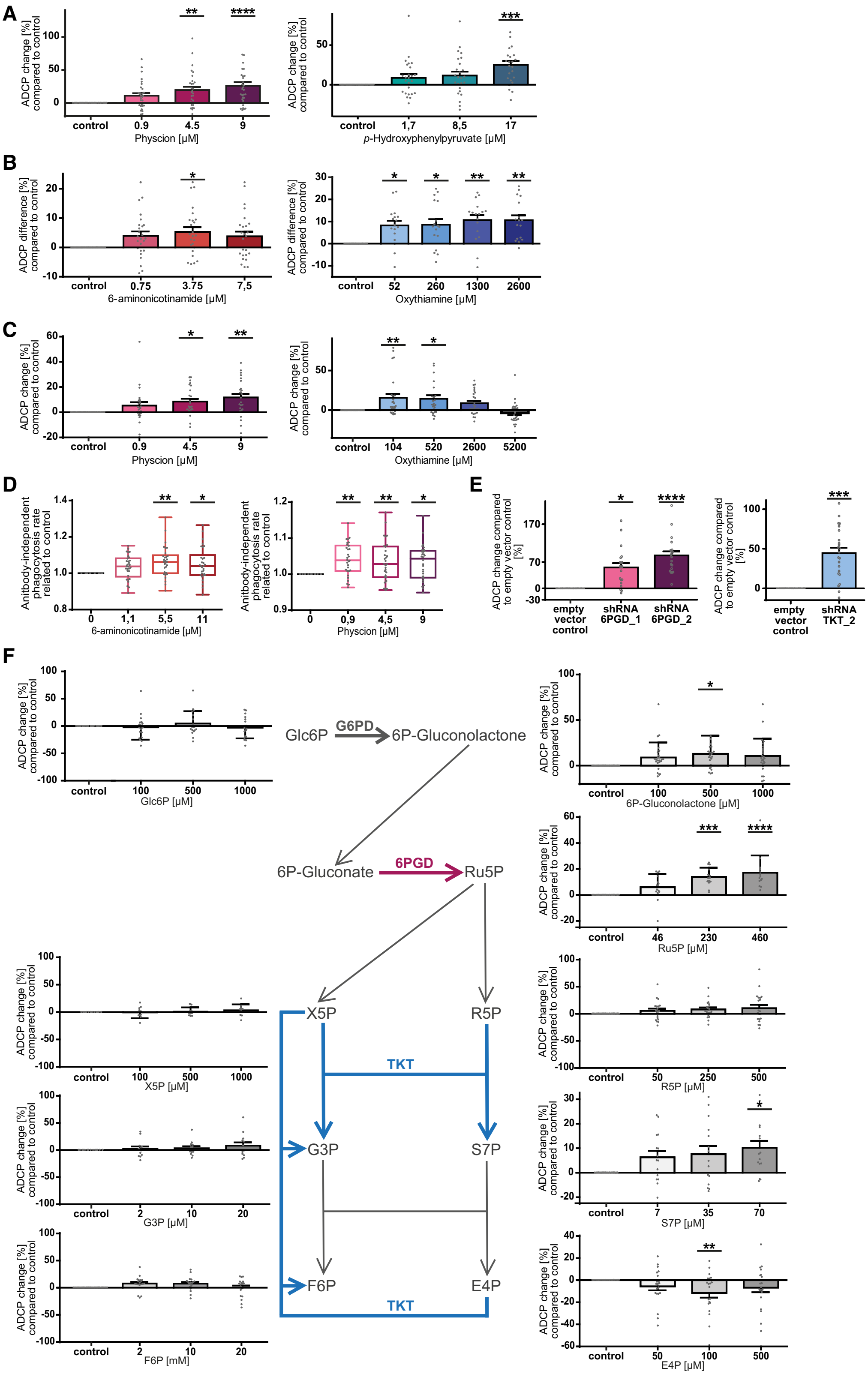

(legend on next page)

type II), also identifying significant induction of ADCP (6-aminonicotinamide $p < 0.05$; oxythiamine $p < 0.01$) (Figure 2B).

Since hypoxia is a functional aspect of the TME *in vivo*, we also conducted ADCP assays under hypoxic conditions (O_2 1.5%) and observed significantly increased ADCP rates (physcion +12%, $p < 0.01$; oxythiamine +20%, $p < 0.01$) (Figure 2C).

To evaluate if PPP inhibition also increases phagocytic capacity of macrophages without the targeting function of antibodies, we assessed antibody-independent cellular phagocytosis (AICP) (Figure 2D) and observed significantly increased AICP rates only by inhibition of the oxidative part of the PPP (6-aminonicotinamide $p < 0.01$, physcion $p < 0.01$) (Figure S3D).

To abrogate off-target effects of the PPP inhibitors, we generated short hairpin RNA (shRNA) knockdown for the respective enzymes in macrophages (Figures S3P–S3S). Silencing of 6Pgd and Tkt significantly increased macrophages' ADCP rates (6Pgd +87%, $p < 0.0001$; Tkt +45%, $p < 0.001$) (Figure 2E).

Altogether, we demonstrate that PPP enzyme inhibition by metabolic inhibitors as well as 6Pgd and Tkt knockdown in macrophages promotes phagocytosis of lymphoma cells and warrants further investigation of the molecular function.

Increased phagocytosis is driven by PPP enzyme inhibition and not by PPP metabolite shifting

To identify which specific components and metabolites of the PPP directly affect phagocytic function, we performed ADCP assays with supplementation of single educts and products of the PPP (Figure 2F). We observed unaltered ADCP rates using the non-exclusive PPP metabolites glucose-6-phosphate (Glc6P), ribose-5-phosphate (R5P), xylulose-5-phosphate (X5P), glyceraldehyde-3-phosphate (G3P), and fructose-6-phosphate (F6P). In contrast, supplementation of the PPP-exclusive glucose-6-phosphate dehydrogenase (G6pd) product 6-phosphogluconolactone significantly increased ADCP rate (+13%, $p < 0.05$), as well as the products of 6pgd (ribulose-5-phosphate [Ru5P]; +17%, $p < 0.0001$) and of Tkt (sedoheptulose-7-phosphate [S7P]; +20%, $p < 0.05$) (Figure 2F; right panel). In contrast, supplementing the Tkt educt erythrose-4-phosphate (E4P) significantly reduced ADCP rate (−12%, $p < 0.01$) (Figure 2F; right panel).

In conclusion, products of Tkt and 6Pgd promote macrophages' phagocytic activity while enzyme educts diminish it, indicating that inhibition of the enzymes itself and not a decrease in their products causes increased phagocytic capacity.

PPP inhibition induces pro-inflammatory polarization and activation in macrophages

To test whether PPP inhibition alters macrophage differentiation and activation, we assessed expression of markers delineating polarization by flow cytometry (Figures 3A and 3B; Table S1). We observed a trend of increased M1-like marker expression and decreased M2-like and TAM marker expression under PPP inhibition and knockdown.

To evaluate macrophage morphology, we performed fluorescent microscopy (Figure 3C). Under PPP inhibition the macrophages underwent a profound change in morphology from a round, centered appearance to a spread and outlaying phenotype with filopodia surrounding the cell body.

As macrophages' metabolic status greatly influences their activity and polarization, we assessed glycolytic and mitochondrial activity with the Seahorse XF Mito Stress test (Figures 3D–3G, Figure S4). We observed a significant increase of the oxygen consumption rate (OCR) ($p < 0.0001$) and extracellular acidification rate (ECAR) ($p < 0.0001$) (Figure 3D) indicating an increased mitochondrial respiration and glycolytic activity and thus an increased metabolic activity of macrophages. Further analysis identified significant increase of mitochondrial basal activity (physcion $p < 0.01$; oxythiamine $p < 0.001$), mitochondrial maximal capacity (physcion $p < 0.05$; oxythiamine $p < 0.01$), glycolytic maximal capacity (physcion $p < 0.05$; oxythiamine $p < 0.001$), and ATP production (physcion $p < 0.05$; oxythiamine $p < 0.0001$) of macrophages (Figures 3E–3G).

Taken together, these data show an activation of macrophages by shifted polarization, cytoskeletal reorganization, and increased metabolic activity under PPP inhibition as possible functional basis of increased phagocytosis.

PPP inhibition changes the proteomic profile of macrophages toward pro-inflammatory activity

To investigate the mediators of increased phagocytic capacity in macrophages, we performed a multi-omics (proteomics, phosphoproteomics, and metabolomics) screening under chemical or shRNA-mediated PPP inhibition.

A uniform regulation pattern of proteins involved in macrophage polarization and activation was observed by the use of independent inhibitors and PPP enzyme knockdowns (Figures 4A and 4B, Table S2). Under compound-mediated PPP inhibition, the anti-inflammatory proteins Ptgs1, Sqstm1, and Ybx3[15–17]

Figure 2. Cross validation of PPP inhibition in macrophages confirms increased ADCP rates

(A–C) ADCP change compared to basal phagocytosis rate of J774A.1 macrophages under inhibition of PPP. (A) Alternative inhibitor physcion of 6-phosphogluconate dehydrogenase (6Pgd) in oxidative part of PPP (red) and *p*-hydroxyphenylpyruvate for inhibition of transketolase (Tkt) in non-oxidative part of PPP (blue). (B) Using human monocyte cell line THP1 and CD20 antibody obinutuzumab under inhibition of 6Pgd by 6-aminonicotinamide (red) and inhibition of Tkt by oxythiamine (blue). (C) ADCP assay performed under hypoxic conditions and inhibition of 6Pgd by physcion (red) or inhibition of Tkt by oxythiamine (blue).

(D) Antibody-independent cellular phagocytosis of hMB cells by J774A.1 macrophages compared to control under inhibition of 6Pgd by 6-aminonicotinamide (left) and physcion (right).

(E) ADCP change compared to basal phagocytosis rate of empty vector control J774A.1 macrophages under shRNA-mediated knockdown of 6Pgd (red) and Tkt (blue).

(F) ADCP change compared to basal phagocytosis rate of J774A.1 macrophages under supplementation of metabolites of the PPP. Enzyme reactions in focus colored in violet (6Pgd) and blue (Tkt). *E4P*, erythrose-4-phosphate; *F6P*, fructose-6-phosphate; *G3P*, glyceraldehyde-3-phosphate; *Glc6P*, glucose-6-phosphate; *R5P*, ribose-5-phosphate; *Ru5P*, ribulose-5-phosphate; *S7P*, sedoheptulose-7-phosphate; *X5P*, xylulose-5-phosphate. Data are shown as mean ± SEM. Technical replicates (A) $n = 30$, (B) $n = 17–25$, (C) $n = 25–28$, (D) $n = 30$, (E) $n = 20–23$, (F) $n = 13–20$; biological replicates (A) $n = 6$, (B) $n = 4–5$, (C) $n = 5–6$, (D) $n = 6$, (E) $n = 4–5$, (F) $n = 3–4$. *p* values were calculated using one-way ANOVA. *$p < 0.05$; **$p < 0.01$; ***$p < 0.001$; ****$p < 0.0001$. See also Figures S1 and S3.

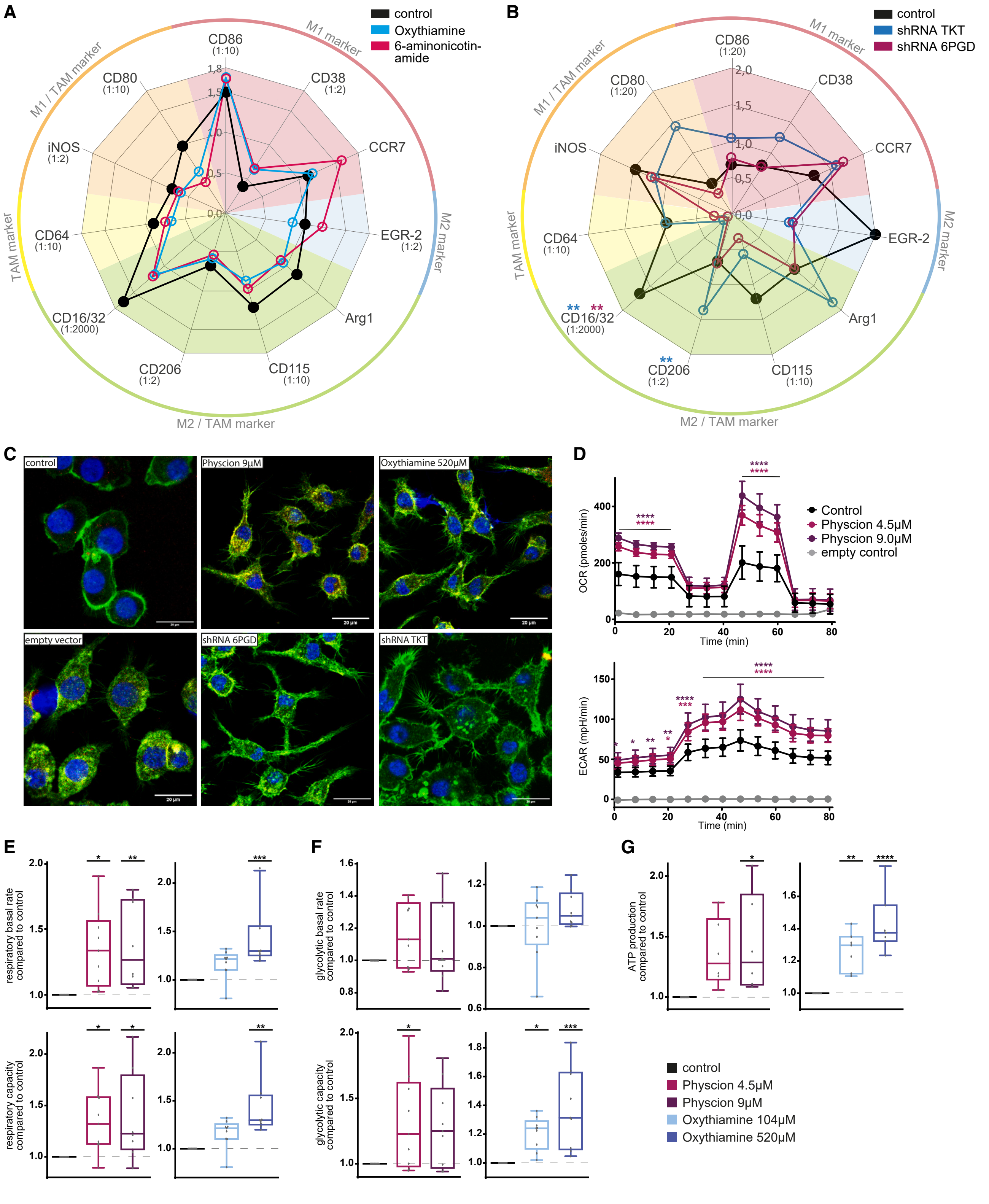

Figure 3. PPP inhibition induces pro-inflammatory polarization and activation in macrophages

(A and B) Radar plot of surface marker expression of J774A.1 macrophages. Expression of characteristic surface marker for different macrophage subtypes measured by immunofluorescent staining. Mean fluorescence intensity (MFI) is depicted. To improve readability, high MFI has been downscaled (factor named in brackets next to marker). (A) Compound mediated PPP inhibition. (B) shRNA-mediated PPP knockdown.

(legend continued on next page)

and the TAM- and M2-typical proteins Hagh and Ezr[18,19] were significantly downregulated, while the pro-inflammatory proteins Pam16 and Gosr1 were significantly upregulated (Figure 4A). By using shRNA, an even more pronounced regulation was seen. Negative regulators of cytokine expression and pro-inflammatory signaling were significantly downregulated while promoters of pro-inflammatory activation were significantly upregulated (Atg16l1, Cast, Csf1r, Cybb, Inppl1, Oas3, Parp14, Fkbp5, Ilf2, Tlr7).[20,21] Moreover, there was a significant increase in protein expression needed for phagocytosis (Actn1, Actr1a, Iqgap3, Itgav, Lrp1, Myl12a, Necap1, Sh3bp1) (Figure 4B; for phosphoproteomic analysis see Table S3).

We performed pathway enrichment analysis for functional annotation (Figures 4C and 4D), showing similar enrichment clusters for both compound-mediated and shRNA-mediated inhibition of the oxidative and the non-oxidative part of the PPP. A strong enrichment was seen for immune activity (Figure 4C) including cytokine signaling, antigen processing, and antigen presentation with up to 124 involved proteins and significantly changed phosphorylation patterns (Figure 4D). Moreover, enrichment in proteins relevant to phagocytosis and cytoskeletal organization was observed. In line with our metabolic flux analysis (Figures 3D–3G), we observed great enrichment for proteins influencing mitochondrial and glycolytic activity (Figure 4C). Particularly analysis of phosphoproteomics uncovered a significant enrichment in signaling pathways important for immune signaling (mitogen-activated protein kinase [Mapk]-Erk, Egf/Egfr, p53, Pi3k-Akt) and metabolic regulation (Pi3k-Akt, Hif1a) (Figure 4D).

To further analyze the impact of altered protein phosphorylation, we performed an adapted upstream kinase analysis on the basis of integrative inferred kinase activity (INKA) analysis (Table S4).[22] The five most inactivated kinases are displayed (Figure 4E), highlighting the decrease of Hck in the normalized upstream kinase score (NUKS). Hck supports M2-like macrophage polarization, TAM activity, tumor growth, and tumor cell evasion[23] and activates the Csf1 receptor (Csf1r).[24] Csf1r signaling likewise induces M2-like macrophage polarization.[25] Csf1r and its downstream kinase Mapk1 were also one of the five most inactivated kinases (Figure 4E). In combined analysis of PPP inhibitors, the Csf1r downstream kinase Ptk2b (Pyk2) was the most negatively regulated kinase (Figure 4F). Accordingly, a significant downregulation of Ptk2b in PPP knockdown macrophages was observed (Figures 4G and S5A). Furthermore, the most downregulated protein in both knockdown macrophages was Sema4d (Figure 4B), which is an activator of the Ptk2b pathway.[26]

Following the Csf1r pathway further downstream (Figure 4H), decreased immune-regulatory gene 1 (Irg1 = Acod1) expression, a major node in immunosuppressive regulation of macrophages, was seen in proteomic analysis (Table S2). Changed Irg1 expression is one possible mechanism leading to altered macrophage activity and phagocytosis.[27] With the exception of Hmox-1, all included signal molecules of the regarded Csf1r pathway were significantly downregulated in proteomic analysis (Figure 4H) (Table S2).

PPP inhibition modulates glycogen metabolism and the immune response signaling axis UDPG-Stat1-Irg1-itaconate of macrophages

Regarding the critical role of Irg1 on macrophage polarization, we aimed to explore the connection between metabolic modulation, Irg1 regulation, and the resulting macrophage phenotype.

PPP and glycogenolysis activity are coupled causing suppression of both pathways if one is inhibited.[28] We quantified glycogen levels identifying significant decreased glycogen amount under all conditions ($p < 0.0001$, Figure 5A). Glycogen metabolism influences signaling regulating Stat1 activity.[28] Thus, we hypothesized that inhibition of PPP would lead to suppression of glycogenolysis with subsequent decreased uridine diphosphate glucose (UDPG) production and thereby to an inhibition of P2y14 expression with following decreased Stat1 activity. Decreased Stat1 activity leads to less Irf1 and thereby to a decreased Irg1 expression, which possibly leads to functional increasing macrophage activity and phagocytosis.[29,30]

To validate this hypothesis, we performed western blot analysis of the hypothesized pathway-associated proteins and identified significant reductions in expression ($p < 0.0001$, Figure 5B) with the highest decline of Irg1 amount (>80%, $p < 0.0001$, Figure S5). The hypothesized pathway linking PPP activity and Irg1 expression is displayed in Figure 5C.

As Irg1 acts as a metabolic enzyme and changes in immune activity are driven by its product itaconate, we investigated the connection between metabolism and enzyme expression by metabolomic assessment (Tables S5 and S6) and compared it to changes in enzyme expression of interest detected in proteomics (Table S2) (Figures 5D and S4C).

The metabolomic screening[31] confirmed a significantly decreased amount of exclusive 6Pgd and Tkt products ribulose-5-phosphate and sedoheptulose-7-phosphate ($p < 0.0001$, Figure 5D) in 6pgd and Tkt knockdown macrophages, while drug-mediated inhibition did not show a significantly decreased amount (Figure S4C).

In line with the hypothesized pathway, a significant downregulation of Irg1 was observed under PPP inhibition ($p < 0.01$) with subsequent significantly decreased amount of itaconate ($p < 0.0001$) (Figure 5E). Itaconate is an inhibitor of succinate dehydrogenase (Sdh). Accordingly, there was a significant

(C) Immunofluorescent microscopy of J774A.1 macrophages under compound-mediated PPP inhibition and shRNA-mediated PPP knockdown. Blue, phalloidin staining of nucleus; green, actin staining of cytoskeleton.

(D–G) Measurement of metabolic activity of J774A.1 macrophages under compound-mediated PPP inhibition by Seahorse analysis. Inhibition of non-oxidative part of PPP by oxythiamine, inhibition of oxidative part of PPP by physcion. (D) One representative example of XF Mito Stress test measurement of ECAR und OCR. (E) Respiratory basal rate and capacity. (F) Glycolytic basal rate and capacity. (G) ATP production. Data are shown in (A and B) as mean of four replicates, in (D) as mean of six replicates in one experiment ±SD, and in (E–G) as mean ± 5–95 percentile. Technical replicates (A and B) $n = 4$, (D) $n = 6$, (E–G) $n = 18–27$; biological replicates (A and B) $n = 4$, (D) $n = 1$, (E–G) $n = 6–9$. p values were calculated using one-way ANOVA, (D) using two-way ANOVA. *$p < 0.05$; **$p < 0.01$; ***$p < 0.001$; ****$p < 0.0001$. See also Figure S4 and Table S1.

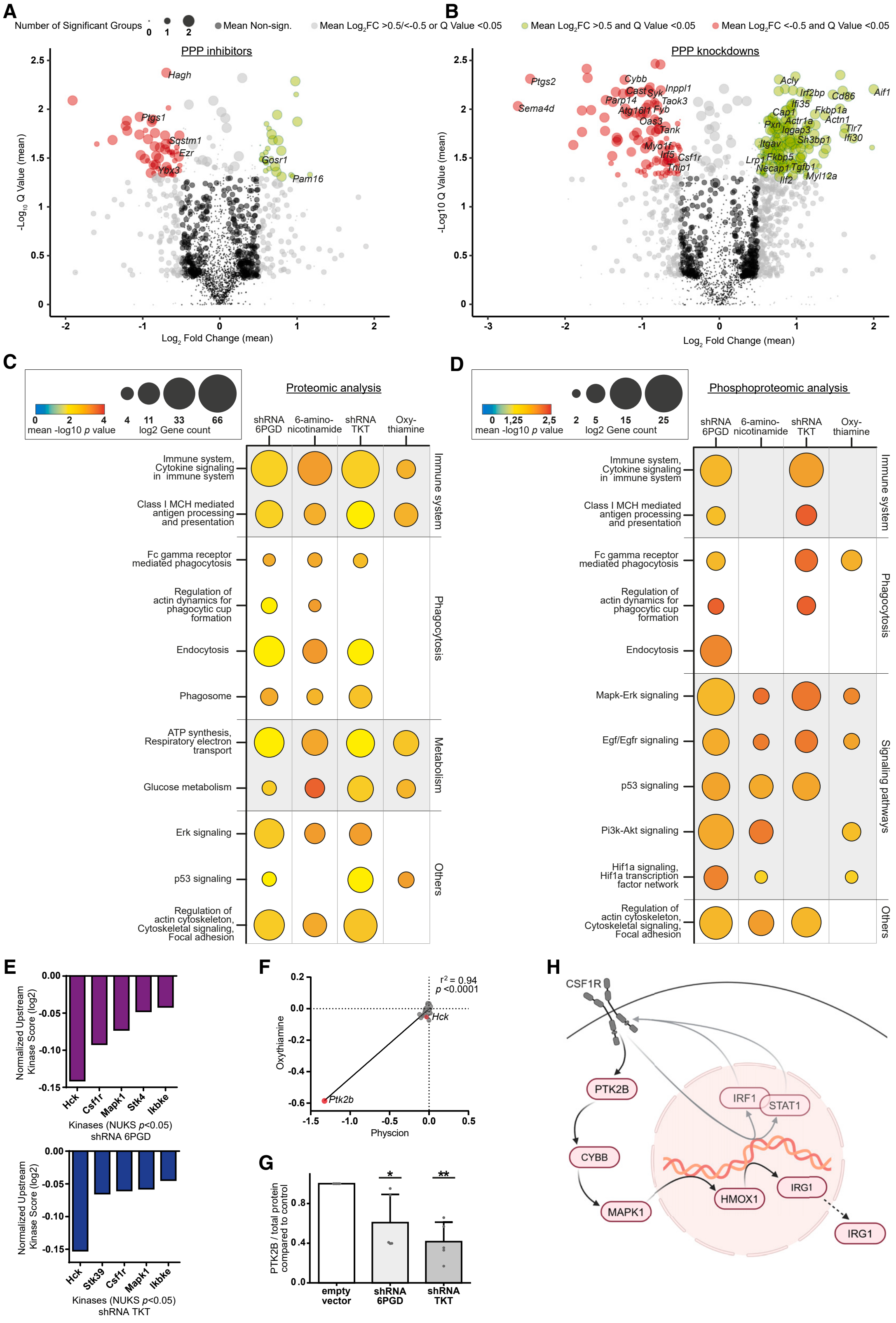

(legend on next page)

decrease of the Sdh educt succinate ($p < 0.001$) and a significant increase of the Sdh product malate ($p < 0.0001$) (Figure 5E). This indicates less suppression of Sdh due to decreased itaconate production by which mitochondrial oxidative activity is increased as observed in the metabolic flux analysis (Figures 3F and 3G). Besides the use of citrate for itaconate production, citrate is also an educt of the ATP citrate lyase (Acly) for acetyl-coenzyme A production. It has been shown that Acly activity is an inducer of macrophage activation and supports pro-inflammatory cytokine production—for example interleukin (IL)-6—in macrophages.[32] In proteomic analysis a significant increase of Acly expression under PPP inhibition was observed ($p < 0.01$, Figure 5E).

Itaconate is widely known as a regulatory immunosuppressive metabolite in macrophages, which promotes anti-inflammatory IL-10 secretion and inhibits pro-inflammatory IL-6 secretion.[27] Moreover, we observed significantly increased nuclear factor κB1 expression under PPP inhibition and knockdown (Table S2), an activator of IL-6 and inhibitor of IL-10 production. In line with these findings, we observed significantly increased IL-6 secretion ($p < 0.05$, Figure 5F) and significantly decreased IL-10 secretion ($p < 0.0001$, Figure 5G) by PPP inhibition.

To further prove the functional role of the Irg1-itaconate pathway, we evaluated the phagocytic activity of macrophages of $Irg1^{-/-}$ knockout mice. A significantly increased phagocytic activity of bone marrow-derived macrophages in ADCP assay *ex vivo* was observed in comparison to $Irg1^{+/+}$ wild-type mice (+34%, $p < 0.05$, Figure 5H).

In conclusion, we connected metabolic activity and immune regulation in macrophages via the UDPG-Stat1-Irg1-itaconate signaling axis provoked by PPP activity. Irg1 downregulation increases macrophage activation via itaconate reduction with subsequent metabolic activation and a pro-inflammatory shift in cytokine secretion. This also leads *in vivo* to an increased phagocytic capacity of macrophages.

PPP inhibition in primary human cells increases phagocytic capacity of macrophages and decreases their bystander function

To translate our findings into human context, we isolated primary human monocytes from healthy donors and differentiated them into macrophages by macrophage colony stimulating factor (M-CSF) under PPP inhibition. After testing cytotoxicity of the PPP inhibitors to primary human macrophages, ADCP assays with non-toxic concentrations of inhibitors were performed. Inhibition of both parts of the PPP significantly increased ADCP rates (physcion +64%; oxythiamine +92%, $p < 0.0001$, Figures 6A and 6B). The human macrophages showed a similar switch toward pro-inflammatory cytokine secretion with significantly increased IL-6 ($p < 0.05$) and significantly decreased IL-10 secretion ($p < 0.0001$) (Figures 6C and 6D).

To address effector function of macrophages in the context of primary human leukemia cells, primary chronic lymphocytic leukemia (CLL) cells of five individual patients were used for ADCP assays. A significant increase of phagocytosis was observed under PPP inhibition (+22%, $p < 0.001$, Figure 6E) and by using knockdown macrophages (Tkt +60%; 6Pgd +92%, $p < 0.0001$, Figure 6F) (Figures S6C and S6D).

To evaluate phagocytosis in a fully human setting, we performed ADCP assay with primary human monocyte-derived macrophages differentiated in the presence of PPP inhibitors and primary CLL patient cells (12 individual patients). A significantly increased phagocytic capacity was observed (+24%, $p < 0.0001$, Figures 6G and S6E). Thereby, we have demonstrated that primary indolent lymphoma and primary human macrophages are also affected by PPP modulation.

Beyond the inefficient phagocytic function, TAMs exert direct supportive effects on tumor cells. CLL cells depend on macrophages as “nurse-like” bystander cells to survive.[33] Macrophages in the microenvironment of CLL are polarized toward tumor-promoting TAMs and support CLL cells by chemokine secretion and immunosuppressive signaling. We therefore evaluated the effect of PPP-inhibited macrophages on primary CLL cells. Interestingly, PPP inhibition in mono-cultured primary CLL cells decreased their viability significantly ($p < 0.0001$, Figure 6H, left), as well as inhibition of the non-oxidative part of the PPP in co-culture ($p < 0.01$, Figure 6H, right).

As TAMs are also important mediators in chemotherapy resistance, we evaluated if the co-cultivation under PPP inhibition affects the susceptibility of primary CLL cells toward apoptosis by chemotherapy. We observed significantly increased bendamustine-induced apoptosis among primary CLL cells under inhibition of both parts of the PPP ($p < 0.001$, Figures 6I–6L) (for individual patient data see Figures S6F–S6J). This boost in apoptosis was achieved by

Figure 4. PPP inhibition changes the proteomic profile of macrophages towards pro-inflammatory activity
(A and B) Volcano plots showing mean change of proteomic transcription under (A) compound-mediated PPP inhibition by 6-aminonicotinamide and oxythiamine compared to untreated J774A.1 macrophages and (B) shRNA-mediated PPP knockdown of 6Pgd and Tkt compared to empty vector control J774A.1 macrophages. Circle size represents number of significantly changed conditions. Red circles: significantly downregulated abundance; green circles: significantly upregulated abundance. Proteins known to participate in immune system are annotated in significant groups.
(C and D) Pathway enrichment analysis of (C) proteomics and (D) phosphoproteomics of J774A.1 macrophages under compound-mediated PPP inhibition and shRNA-mediated PPP knockdown. Protein count in listed pathways represented in circle size, mean $-\log 10$ p value represented in heatmap analysis.
(E and F) Analysis of significantly negative changed protein activity in *normalized upstream kinase score* (*NUKS*). (E) Top five most downregulated enzymes in *NUKS* analysis under shRNA-mediated PPP knockdown of 6Pgd and Tkt and (F) integrative analysis of compound-mediated PPP inhibition by physcion and oxythiamine.
(G) Western blot analysis of Ptk2b expression in J774A.1 macrophages under shRNA-mediated PPP knockdown of 6Pgd and Tkt compared to empty vector control.
(H) Scheme of hypothesized mechanism leading to pro-inflammatory phenotype of macrophages. In (G) data are shown as mean ± SEM. Technical replicates (A–F) $n = 1$, (G) $n = 5$; biological replicates (A–F) $n = 3$, (G) $n = 5$. p values in (G) were calculated using one-way ANOVA. *$p < 0.05$; **$p < 0.01$; ***$p < 0.001$; ****$p < 0.0001$. See also Tables S2–S5.

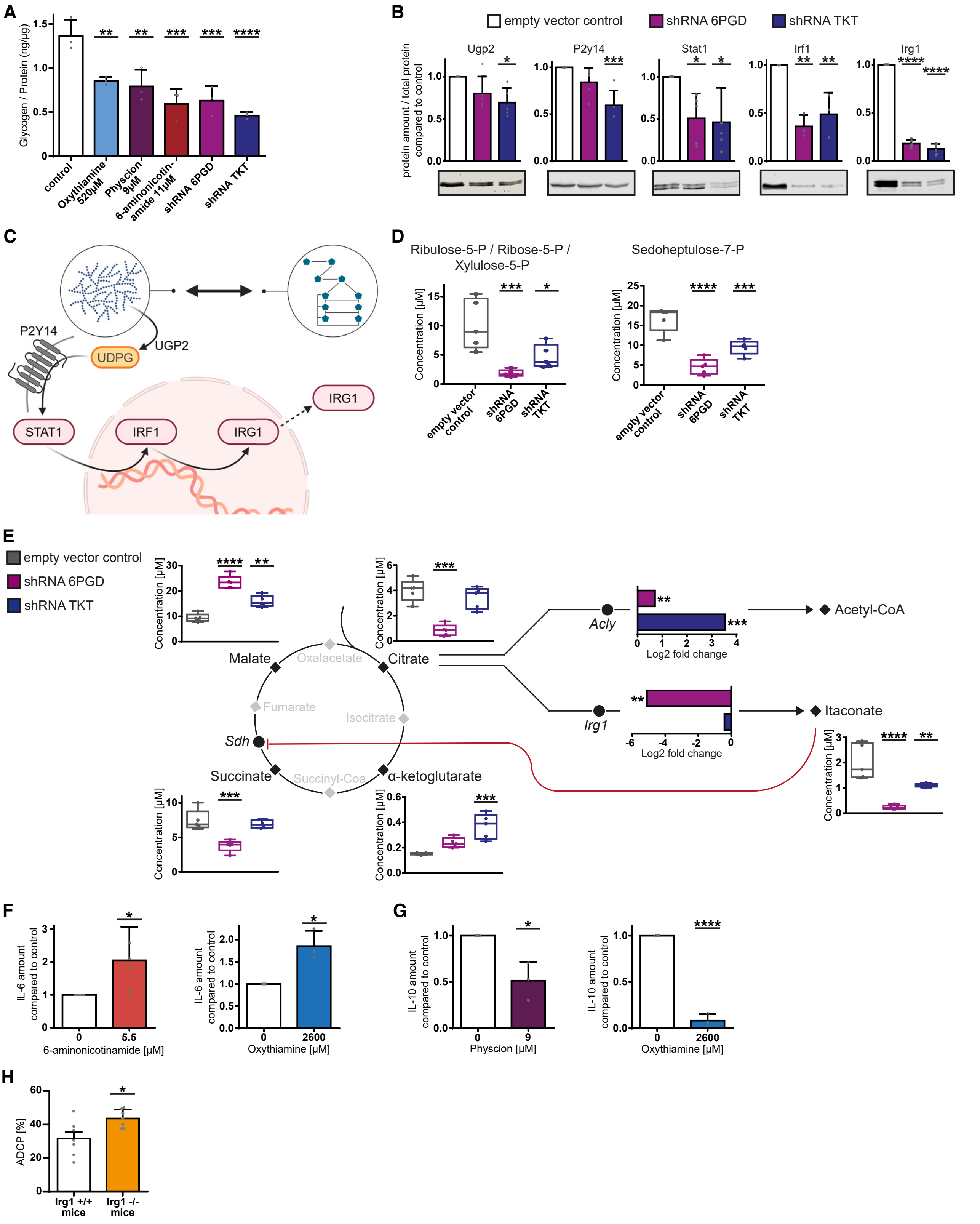

(legend on next page)

PPP inhibition in co-culture ($p < 0.001$, Figures 6I and 6K) and by macrophages pre-treatment before exposing them to primary CLL cells ($p < 0.01$, Figures 6J and 6L).

These observations underline the role of altered macrophage support under PPP inhibition in the TME such as direct leukemia cell support or resistance to chemotherapy.

PPP inhibition increases macrophages' maturation and pro-inflammatory polarization *in vivo*

To evaluate if PPP inhibition preserves its effect on macrophages *in vivo*, we treated C57BL/6J mice with the PPP inhibitor S3 (1-hydroxy-8-methoxy-anthraquinone). S3 is a more stable derivate of the 6Pgd inhibitor physcion.[13]

Investigating the myelopoiesis under PPP inhibition, a significant increase of cells in the LSK compartment (Lin^-, $Sca\text{-}1^+$, $c\text{-}Kit^+$) was seen ($p < 0.0001$, Figure 7A). PPP inhibition significantly increased the amount of hematopoietic stem cells (HSCs; $p < 0.05$, Figure 7B) and multipotent progenitor (MPP) cells (Figure 7B), including MPP pools known to fuel the myeloid compartment (MPP2 $p < 0.001$; MPP3 $p < 0.01$; MPP5 $p < 0.01$, Figure 7B).[34,35] This coincided with a significantly increased frequency of myeloid progenitor cells ($p < 0.01$, Figure 7C) and macrophages ($p < 0.001$, Figure 7C) in the bone marrow, indicating propelled myelopoiesis.

We further investigated the polarization of *in vivo* macrophages (Table S8) and detected a shift away from M2-like subtype with significantly decreased Arg1 expression ($p < 0.05$) and toward pro-phagocytic M1-like subtype with significantly increased CD38 expression ($p < 0.05$) in unstimulated peritoneal macrophages (Figure 7D) (for other compartments see Figure S7A). These findings are in line with our *in vitro* observations.

Moreover, *in vivo* PPP inhibition significantly increased phagocytic activity of bone marrow-derived macrophages in ADCP assay *ex vivo* (+74%, $p < 0.001$, Figure 7E).

Altogether, we have shown that PPP inhibition *in vivo* activates macrophages' inflammatory polarization and maturation as well as their phagocytic capacity, which increases their anti-tumor function *in vivo*.

PPP inhibition boosts anti-leukemic treatment and thereby prolongs survival in an aggressive lymphoma mouse model

To focus on the therapeutic effect of PPP inhibition *in vivo*, we evaluated treatment effects in an aggressive lymphoma mouse model.

We used the humanized double-hit lymphoma mouse model (hMB),[36] which is amenable for modeling human-specific antibody therapy. We treated the mice with the therapeutic antibody alemtuzumab and the PPP inhibitor S3. As the lymphoma reflects aggressive disease, untreated mice died rapidly after tumor cell injection (median overall survival [mOS] 22 days, Figure 7F). By treatment with S3 only, this rapid tumor progression persisted. As shown in our previous work, treatment with alemtuzumab increases survival significantly in this aggressive lymphoma mouse model[5] (mOS 25 days, $p = 1.4e{-}05$, Figures 7F and S7B). By adding the PPP inhibitor S3 to alemtuzumab, an additional significant prolongation of mouse survival was achieved in comparison to antibody treatment only (mOS 27 days, $p = 0.0059$, Figures 7F and S7B) with a stable increased number at risk up to day 25 (survival of 88%, Figure 7F). Immunohistochemical analysis of spleens showed a marked reduction of $CD19^+$ lymphoma cell infiltration with concomitant increase of $CD68^+$ macrophage infiltration after treatment with alemtuzumab and S3 in comparison to vehicle control (Figures 7G and S7C).

We finally demonstrated *in vivo* that PPP inhibition in the context of a highly aggressive lymphoma model increases the efficacy of antibody therapy to prolong overall survival significantly.

DISCUSSION

TAMs are key drivers in various cancers associated with poor outcome and diminished efficacy of immunotherapies.[1,3,5] The influence of glucose and mitochondrial metabolism on macrophages' polarization and activity has been established.[8,9,37] Activation of the PPP in macrophages has been implicated in immune tolerance and granuloma formation.[38,39] However, no

Figure 5. PPP inhibition modulates glycogen metabolism and the immune response signaling axis UDPG-Stat1-Irg1-itaconate of macrophages

(A) Total glycogen amount in J774A.1 macrophages under compound-mediated PPP inhibition and shRNA-mediated knockdown of 6Pgd and Tkt.

(B) Western blot analysis of protein expression of hypothesized connecting pathway in J774A.1 macrophages under shRNA-mediated knockdown of 6Pgd and Tkt compared to empty vector control. Mean expression displayed in bar graph analysis and one representative western blot example.

(C) Scheme of working hypothesis of PPP metabolism modulating immune response.

(D) Amount of 6pgd product ribulose-5-phosphate and Tkt product sedoheptulose-7-phosphate under shRNA-mediated inhibition of 6pgd and Tkt in J774A1 macrophages.

(E) Metabolomic analysis of tricarboxylic acid cycle and citrate metabolism with display of enzyme expression of key enzymes under shRNA-mediated PPP knockdown of 6Pgd and Tkt compared to empty vector control J774A.1 macrophages. Amount of metabolites displayed in box and whiskers. Change in enzyme expression displayed in bar graphs. Genes: succinate dehydrogenase (Sdh), ATP citrate lyase (Acly), immunoregulatory gene 1 (Irg1 = Acod1).

(F and G) Cytokine expression under 6Pgd inhibition by 6-aminonicotinamide or physcion and Tkt inhibition by oxythiamine in J774A.1 macrophages. (F) IL-6 expression compared to untreated control. (G) IL-10 expression compared to untreated control.

(H) ADCP assay of bone marrow-derived macrophages of $Irg1^{+/+}$ wild-type mice and $Irg1^{-/-}$ knockout mice. Macrophages differentiated out of femoral bone marrow with M-CSF. In (A), (B), and (F–H), bar plots are shown as mean ± SEM; in (D and E) metabolite amount is shown as minimum to maximum and protein expression is shown as calculated −Log2 fold change of control and knockdown macrophages. Technical replicates (A) $n = 15$, (B) $n = 3$–6, (D) $n = 3$, (E) $n = 3$, (F and G) $n = 9$–18, (H) $n = 35$; biological replicates (A) $n = 3$, (B) $n = 3$–6, (D) $n = 3$, (E) $n = 3$, (F and G) $n = 3$–6, (H) $n = 7$. p values were calculated in (A, B, D, and E) using one-way ANOVA, protein expression in (E) using student's t test, and in (F–H) using unpaired t test. *$p < 0.05$; **$p < 0.01$; ***$p < 0.001$; ****$p < 0.0001$. See also Figures S4 and S5 and Tables S6 and S7.

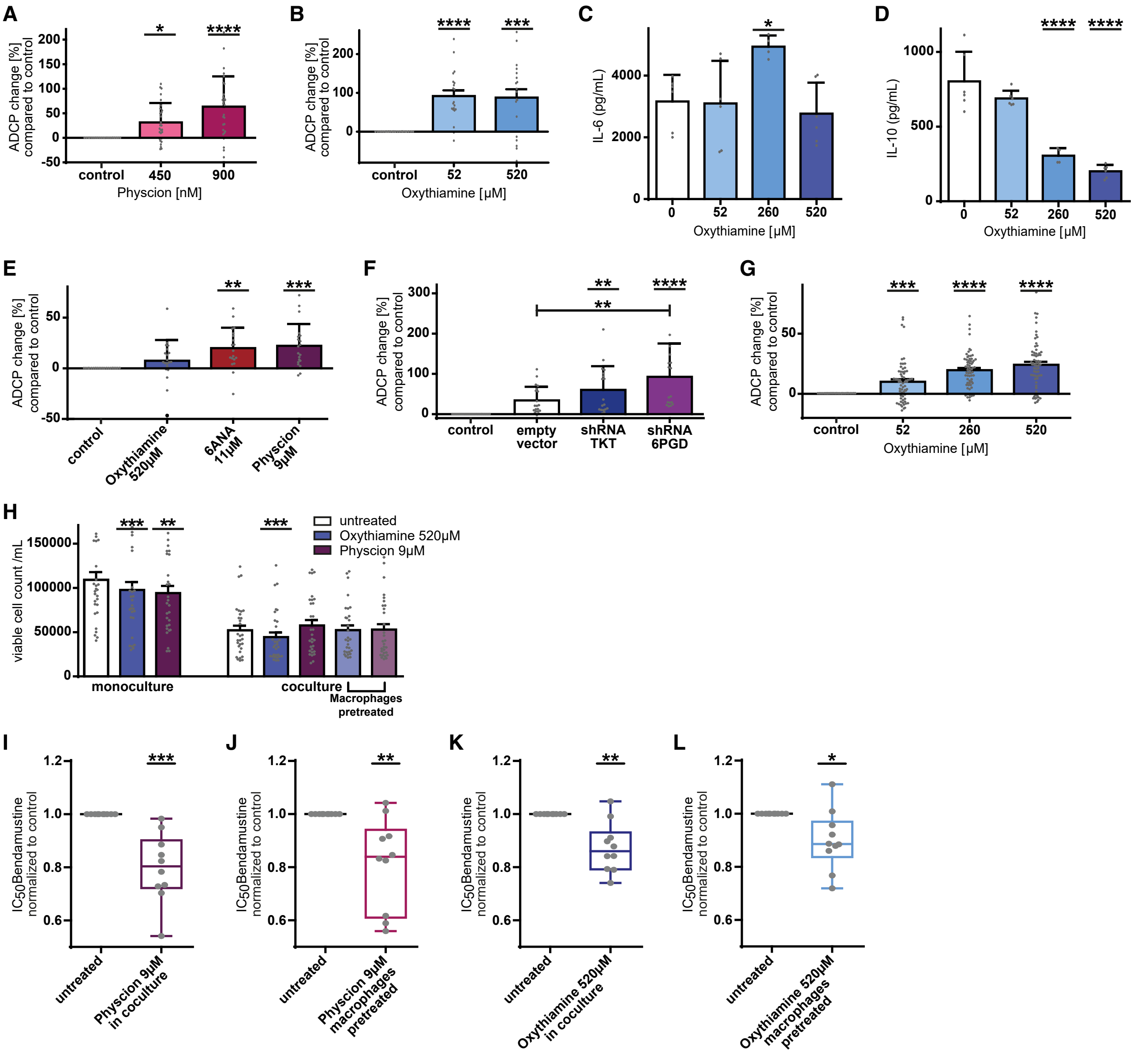

Figure 6. PPP inhibition in primary human cells increases phagocytic capacity of macrophages and decreases their tumor-supportive bystander function

(A and B) ADCP change compared to basal phagocytosis rate of human monocyte-derived macrophages. (A) ADCP change of monocyte-derived macrophages differentiated in the presence of physcion and M-CSF, (B) ADCP change of monocyte-derived macrophages differentiated in the presence of oxythiamine and M-CSF.

(C and D) Cytokine expression of monocyte-derived macrophages differentiated in the presence of oxythiamine and M-CSF. (C) IL-6 expression, (D) IL-10 expression.

(E and F) ADCP change of J774A.1 macrophages phagocyting primary CLL patient cells compared to basal phagocytosis rate. (E) ADCP change under compound-mediated PPP inhibition. (F) ADCP change under shRNA-mediated PPP knockdown.

(G) ADCP change of monocyte-derived macrophages differentiated in the presence of oxythiamine and M-CSF phagocyting primary CLL patient cells compared to basal phagocytosis rate.

(H) Viability of primary CLL patient cells after incubation with PPP inhibitors physcion or oxythiamine in mono-culture and in co-culture with J774A.1 macrophages. In co-culture setting, cells were treated in parallel or macrophages were pre-treated before onset of co-culture.

(I–L) Half maximal inhibitory concentration (IC_{50}) for individual primary CLL patient cell samples to bendamustine treatment compared to control. Cells were incubated with bendamustine after protective macrophage co-culture with untreated J774A.1 macrophages vs. PPP inhibition. (I and J) Inhibition of 6Pgd in oxidative part of PPP by physcion, (I) co-culture treatment, (J) macrophage pre-treatment. (K and L) Inhibition of Tkt in non-oxidative part of PPP by oxythiamine,

(legend continued on next page)

functional implications of the PPP in TAM regulation have been established nor the effects of PPP inhibition on immune regulation have been identified. Here, we show that modulation of the PPP in TAMs serves as a robust regulator of phagocytic function and macrophage activity and prolongs survival in aggressive B cell lymphoma therapy.

Metabolic inhibition screening in lymphoma-macrophage cocultures emphasized detrimental effects on macrophage function for the majority of investigated pathways. Only inhibition of the PPP showed a significant increase of phagocytosis with a synergistic effect on effector and target cells. This was true across various *in vitro* and *in vivo* macrophage model systems of both murine and human descent by compound and genetic targeting. Even though the time-dependent effects of PPP inhibition differ between chemical compounds and genetic targeting, we observed similar phenotype under compound-mediated as well as shRNA-mediated PPP inhibition. The increased phagocytosis rate appeared also under hypoxic conditions as an approximation of physiological status of therapy-refractory niches of lymphoma—the lymph nodes and bone marrow[40,41]—indicating therapeutic efficacy by metabolic inhibition in contrast to other therapy modalities in these niches.

Previous reports demonstrated reduced cancer and leukemia cell growth in mice upon PPP inhibition.[13,42] Especially in CLL, macrophages play a pivotal role as supportive bystander cells in the TME, without which CLL cells would undergo spontaneous apoptosis.[33] We have shown that PPP inhibition diminishes this pro-survival bystander function of macrophages and acts as a sensitizer to genotoxic regimens.

Alterations of the PPP enzymes 6PGD and TKT have been previously described in many cancer types.[43–47] Overexpression of TKT was closely associated with aggressive hepatocellular carcinoma features,[48] and 6PGD was shown to promote metastasis,[49] while suppression of 6Pgd attenuates cell proliferation and tumor growth[42] and overcomes cisplatin resistance[50] and Tkt inhibition sensitizes cancer cells to targeted therapy and reduces growth of metastatic lesions.[48] PPP inhibition by physcion, S3, or 6-aminonicotinamide has demonstrated anti-tumorigenic effects in several solid tumor types and chemotherapeutic-resistant acute myeloid leukemia cells,[47,51,52] without affecting non-malignant cells.[42] Moreover, 6Pgd inhibition in $CD8^+$ T cells led to an increased effector function with higher tumoricidal activity.[53]

We have previously shown that macrophage effector polarization is crucial in therapeutic antibody-based regimens of B cell lymphoma and can be modulated.[54–56] We now identified macrophage metabolism as an essential switch of macrophage effector function in lymphoma.

We demonstrated that increase of phagocytosis is driven by PPP enzyme inhibition, rather than metabolite accumulation. Non-exclusive PPP metabolites did not influence phagocytosis, possibly due to degradation via glycolysis (G3P, F6P) or nucleotide synthesis (R5P) before entering PPP flux. In contrast, exclusive PPP metabolites altered phagocytosis activity: supplementation of the G6pd product 6-phosphogluconate, the 6Pgd product ribulose-5-phosphate, and the Tkt product sedoheptulose-7-phosphate increased phagocytosis, while supplementation of the Tkt educt erythrose-4-phosphate decreased phagocytic activity in macrophages. This points to a feedback inhibition of the respective PPP enzymes and emphasizes the enzyme inhibition as driving force for increased phagocytosis.

PPP is a central linker between glucose metabolism, amino acid biosynthesis, fatty acid metabolism, and redox homeostasis.[43] A gain in metabolic activity was observed under PPP inhibition with increased glycolytic and mitochondrial capacity causing enhanced ATP production, fueling macrophages' activation. An increase of glycolysis is well described within the phenotypical switch to pro-inflammatory macrophages.[57] We observed profound alteration of morphology and macrophage polarity, demonstrated by a decrease of markers associated with M2-like macrophages and TAMs, which represent immunosuppressive and tumor-promoting macrophage subtypes,[3,58,59] while exclusive M1 marker, expressed on pro-inflammatory macrophages, was increased.[60–63]

In total, the restriction of one metabolic pathway—the PPP—gives rise to numerous paths of activation, which renders a profound alteration of phenotype and particular phagocytic activity in macrophages. Thereby the anti-tumor function could be improved from independent directions.

Our detailed multi-omics and functional analysis provides evidence that these effects are directly related to 6Pgd and Tkt enzyme activity loss, which polarizes macrophages to a pro-inflammatory phenotype through downregulation of Stat1 and Irg1. The functional switch between PPP enzyme activity and subsequent polarization program is Csf1r expression and activity of glycogen metabolism.

Csf1r activation induces Hmox-1 expression,[64,65] which induces Irg1 expression,[66] a central inhibitory regulator of macrophage activation.[27] We demonstrated significant downregulation of Csf1r pathway proteins. Via Csf1r signaling, macrophages are polarized toward an M2-like or TAM phenotype by directly activating Erk1/2 (Mapk1/2) and Hck signaling.[24,25,67–69] Both, Mapk1 and Hck activity was shown to be decreased under PPP inhibition in upstream kinase analysis.

Considering the relevant role of Csf1r in macrophage ontogeny, activation, and polarization, reduced Csf1r expression might be responsible for macrophage activation under PPP inhibition. Therefore, Csf1r blockade might be a promising strategy to increase macrophage activity in the context of tumor therapy. Several CSF1R inhibitors are currently under clinical investigation.[70] Nevertheless, as CSF1R is a macrophage-exclusive receptor, only a macrophage-exclusive effect could be achieved by using CSF1R inhibition, in contrast to the previously described multi-cellular effects of 6PGD and TKT inhibition.

PPP inhibition has been functionally linked to inhibition of glycogenolysis causing decreased UDPG production.[28] UDPG

(K) co-culture treatment, (L) macrophage pre-treatment. In (A–H) data are shown as mean ± SEM, in (I–L) as minimum to maximum. Technical replicates (A) $n = 28$, (B) $n = 20$, (C and D) $n = 18$, (E and F) $n = 20$, (G) $n = 65$, (H) $n = 30$, (I–L) $n = 30$; biological replicates (A) $n = 6$, (B) $n = 4$, (C and D) $n = 6$, (E and F) $n = 5$, (G) $n = 12$, (H) $n = 10$, (I–L) $n = 10$. p values were calculated in (A–G) using one-way ANOVA, in (H) using repeated measures (RM) one-way ANOVA, and in (I–L) using paired t test. *$p < 0.05$; **$p < 0.01$; ***$p < 0.001$; ****$p < 0.0001$. See also Figure S6.

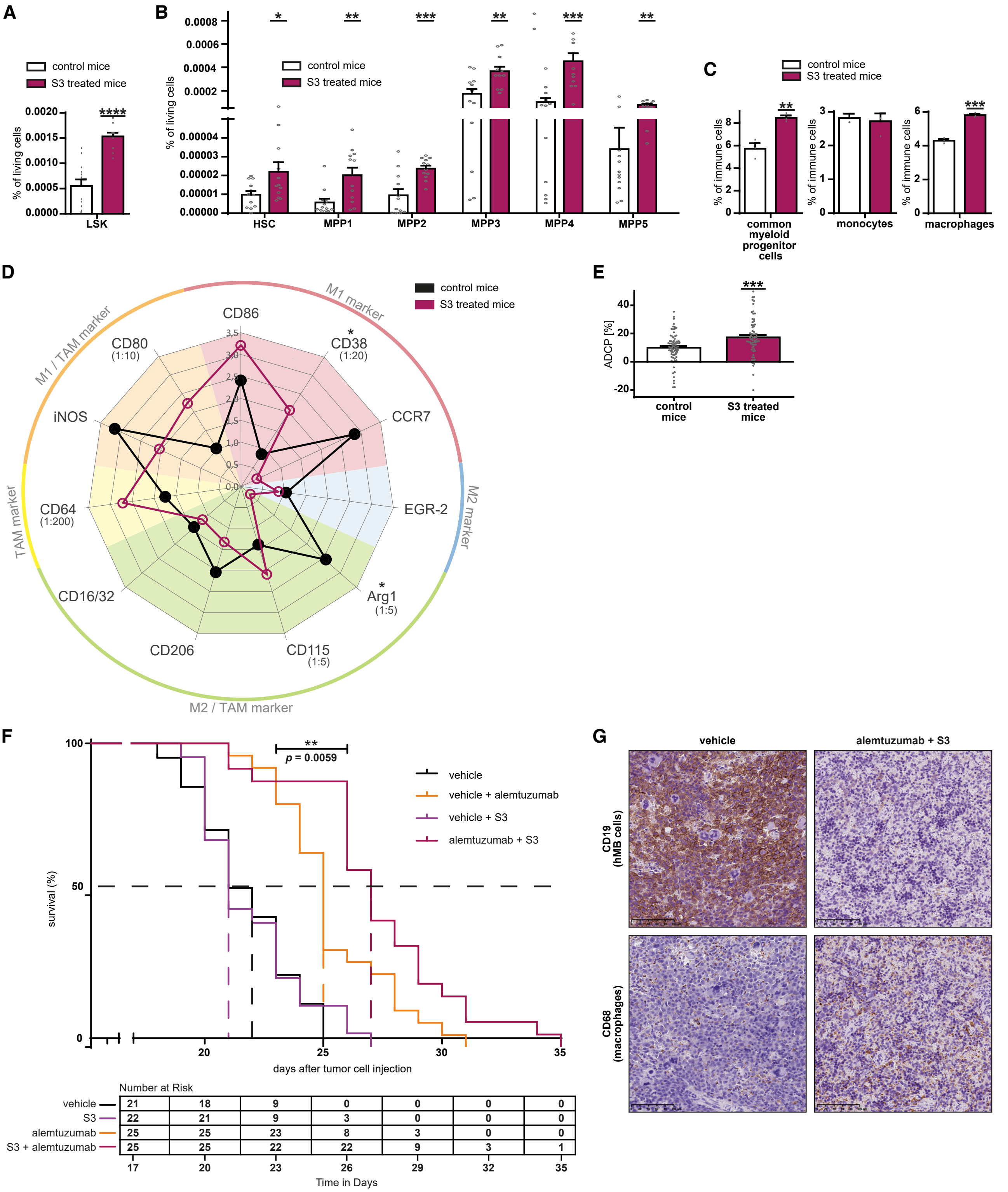

(legend on next page)

as a signaling molecule activates the P2y14 receptor (P2y14r). P2y14r activation in turn increases Stat1 expression and Mapk1 phosphorylation.[71] Considering that Stat1 is a major regulator of Irf1 expression,[29] which is the transcription factor of Irg1,[72] the signaling cascade induced by PPP inhibition in macrophages is causing an altered Irg1 expression. Irg1 is almost exclusively expressed in activated immune cells and a key driver of immune inhibition via itaconate production.[27] Itaconate inhibits glycolysis and mitochondrial activity by Sdh inhibition[73] and promotes anti-inflammatory macrophage phenotype[74] and tumor growth by increased reactive oxygen species secretion by TAMs.[75] In contrast to the publication of Ma et al.,[28] we observed a decreased amount of glycogen under PPP inhibition, but simultaneously increased glycolysis as a possible indicator for a shift of glucose processing causing decreased glycogen synthesis and glycogenolysis. Subsequently, we observed decreased expression of all UDPG-Stat1-Irg1-itaconate pathway proteins under PPP inhibition, particularly lower amounts of itaconate and an increased Sdh and Acly activity. Acly activity is known to induce macrophage activation and pro-inflammatory cytokine production.[32] These changes subsequently resulted in a pro-inflammatory cytokine switch, phenotypic shift toward M1-like macrophages, and diminished primary leukemia cell support. The metabolic alterations observed here support the hypothesis of the connection between PPP inhibition, itaconate abundance, and immune regulation.

Furthermore, the transcription factor Irf1 induces inducible nitric oxide synthase (iNos) expression,[72] consistent with the observed decreased iNos expression under PPP inhibition. High iNos expression and activity have been correlated with malignancy and poor survival in several solid tumors and leukemia.[76]

Despite the pro-inflammatory transcriptional function of Stat1 in LPS-stimulated macrophages, we show an alternative mechanism of macrophage activation by metabolic depression of the anti-inflammatory properties of Stat1 via itaconate regulation leading to a pro-phagocytic phenotype of macrophages. In TAMs, Stat1 has been shown to be the generator of the blended M1/M2 phenotype and supporter of the anti-inflammatory and pro-tumorigenic properties.[77,78]

As reduced expression of Irf1 also influences Stat1 and Csf1r expression,[79] Irf1 appears as the junction between all recapitulated pathways, which are leading to the changed macrophage activity and polarization.

Taken together, these signaling connections show a narrow network, which links PPP inhibition and immune regulation in macrophages and cancer cells.

We demonstrated a highly significant increase of phagocytosis in primary human cells, indicating efficacy for potential clinical use.

To model lymphoma in patients, we performed *in vivo* experiments with the PPP inhibitor S3, whose low toxicity and high effectiveness in treatment of other tumor entities were demonstrated before.[13,42] In the aggressive lymphoma mouse model,[36] PPP inhibition has an amplification effect on antibody therapy leading to significant prolonged overall survival in comparison to antibody treatment only with associated increased macrophage lymphoma infiltration. PPP inhibition *in vivo* increased myelopoiesis and gave rise to progenitor cell expansion, indicating increased provision of a variety of immune cells. Macrophages displayed pro-inflammatory polarization and significantly increased phagocytic capacity after PPP inhibition *in vivo*.

We have proven *in vivo* the efficacy of PPP inhibition leading to macrophage activation and improving therapy response by antibody-mediated phagocytic clearance of lymphoma with prolongation of overall survival.

In conclusion, PPP inhibition may serve as immune-modulatory therapy repolarizing macrophages. We demonstrated metabolic modulation as a key mechanism of macrophage regulation. PPP inhibition causes diminished Irg1 expression leading to reduced anti-inflammatory itaconate production. Our work indicates PPP inhibition as a dual-principle therapy targeting cancer cells and their immune-microenvironment simultaneously, with implications for cancer treatment, especially in the context of antibody-based regimens.

Limitations of the study

This study has several important limitations to consider. The screening process was conducted primarily from the perspective of ADCP and emphasized a therapeutic angle, which may

Figure 7. PPP inhibition increases myelopoiesis, macrophages' maturation, and pro-inflammatory polarization *in vivo* and boosts anti-leukemic treatment response in an aggressive humanized lymphoma mouse model

(A) Progenitor cell compartment LSK (Lin$^-$, Sca-1$^+$, c-Kit$^+$) in bone marrow of C57BL/6J mice treated with vehicle (control) or PPP inhibitor S3 i.p. for 7 days.

(B) Multipotent progenitor (MPP) subsets in bone marrow of C57BL/6J mice treated with vehicle (control) or PPP inhibitor S3 intraperitoneally (i.p.) for 7 days. HSC (CD34$^-$, CD48$^-$, CD150$^+$, CD135$^-$), MPP1 (CD34$^+$, CD48$^-$, CD150$^+$, CD135$^-$), MPP2 (CD34$^+$, CD48$^+$, CD150$^+$, CD135$^-$), MPP3 (CD34$^+$, CD48$^+$, CD150$^-$, CD135$^-$), MPP4 (CD34$^+$, CD48$^+$, CD150$^-$, CD135$^+$), MPP5 (CD34$^+$, CD48$^-$, CD150$^-$, CD135$^-$).

(C) Percentage of myeloid lineage cells of whole cell amount in bone marrow of NSG mice transfected with hMB cells, treated with vehicle or PPP inhibitor S3 i.p. for 12 days, and euthanized on day 15. Common myeloid progenitor cells (CD41$^+$, CD34$^+$), monocytes (Ly6c$^+$, CX3CR1$^+$), macrophages (F4/80$^+$, CD64$^+$).

(D) Expression of characteristic surface marker for different macrophage subtypes on peritoneal macrophages measured by immunofluorescent staining. Mean fluorescence intensity (MFI) is depicted. To improve readability, high MFI has been downscaled (factor named in brackets next to marker). C57BL/6J mice treated with vehicle (control) or PPP inhibitor S3 i.p. for 7 days.

(E) ADCP assay of bone marrow-derived macrophages. C57BL/6J mice treated with vehicle (control) or PPP inhibitor S3 i.p. for 7 days, macrophages differentiated out of femoral bone marrow with M-CSF.

(F) Survival curve of aggressive lymphoma (hMB) bearing mice treated with PPP inhibitor S3 +/− therapeutic antibody alemtuzumab.

(G) One representative example of immunohistochemical staining of hMB cells (CD19$^+$) and macrophages (CD68$^+$) in spleen of aggressive lymphoma (hMB) bearing mice treated with vehicle or alemtuzumab + S3. In (A–C and E) data are shown as mean ± SEM, in (D) data are shown as mean of ten replicates. Technical replicates (A and B) n = 12, (C) n = 3, (D) n = 9–10, (E) n = 70–75, (F) n = 21–25, (G) n = 4; biological replicates (A and B) n = 12, (C) n = 3, (D) n = 9–10, (E) n = 14–15, (F) n = 21–25, (G) n = 4. p values were calculated in (A–E) using unpaired t test and in (F) using Benjamini-Hochberg test. *$p < 0.05$; **$p < 0.01$; ***$p < 0.001$; ****$p < 0.0001$. See also Figure S7 and Table S8.

have limited the scope of the findings. While the PPP was a focus, its role in modulating lymphoma progression requires further investigation to fully understand its impact on the TME. The study concentrated on macrophages as targets for PPP inhibition. The effects of this inhibition on other immunotherapeutic approaches, such as bispecific antibodies and CAR-T cells, remain to be demonstrated. Crucially, the role of PPP inhibition in human patients has yet to be established through clinical trials. These limitations highlight areas for future research to build upon this study's results and provide a more comprehensive understanding of PPP inhibition in lymphoma treatment.

RESOURCE AVAILABILITY

Lead contact

Further requests for information should be directed to and will be fulfilled by the lead contact, Christian P. Pallasch (christian.pallasch@uk-koeln.de).

Materials availability

This study did not generate new unique reagents.

Data and code availability

- Proteomic and phosphoproteomic data have been deposited at PRIDE:PXD042428. Metabolomic data have been deposited at UCSD Metabolomics Workbench: ST003516.
- The data are publicly available as of the date of publication. Accession numbers are listed in the key resources table.
- This paper does not report original code.
- Any additional information required to reanalyze the data reported in this paper is available from the lead contact upon request.

ACKNOWLEDGMENTS

We are indebted to our patients who contributed tissue and blood samples to this study. This work was funded by the Deutsche Forschungsgemeinschaft (DFG, German Research Foundation) KFO286 and SFB1530 (SFB-Geschaeftszeichen – 455784452, Project B02). C.P.P. was supported by the "Foerderprogramm Nachwuchsforschungsgruppen NRW 2015–2021," CAP Program of the Center for Molecular Medicine Cologne, the "Deutsche Jose-Carreras Leukaemiestiftung e.V." (DJCLS 07R/2021), and a research grant by Gilead Sciences. A.C.B. was supported by Studentische Forschungsfoerderung/Begabtenfoerderung" of Koeln Fortune program of the medical faculty of University of Cologne. We would like to thank Thomas Wunderlich for advice and critical reading. We are grateful for technical assistance from the CECAD imaging and animal facilities. Graphics have been created in BioRender: graphical abstract BioRender.com/y35w412, Figure 1A BioRender.com/n22i592, Figure 4H BioRender.com/c70j052, and Figure 5C BioRender.com/b93t764.

AUTHOR CONTRIBUTIONS

Study design, A.C.B., N.N., and C.P.P.; data analysis and acquisition, A.C.B., S.B., M.K., M.M., S.C., R. Brinker, H.-H.B., D.V., L.A., J.L.N., R.L., C.R.C.P., J.S., F.P., A.F., and A.V.; bioinformatic analysis, A.C.B., E.I., S.B., J.L.N., A.V., and M.K.; analytical tools, R. Büttner, H.W., M.K., A.V., and C.B.; clinical samples and annotation, C.P.P. and M.H.; study supervision and funding, C.P.P.; manuscript preparation, A.C.B., S.B., and C.P.P.

DECLARATION OF INTERESTS

The authors declare no competing interests.

STAR★METHODS

Detailed methods are provided in the online version of this paper and include the following:

- KEY RESOURCES TABLE
- EXPERIMENTAL MODEL AND STUDY PARTICIPATION DETAILS
  - Mouse strains
  - Cell lines
  - Primary cells
  - Microbe strains
- METHOD DETAILS
  - Antibody-dependent cellular phagocytosis assay (ADCP)
  - Antibody-independent cellular phagocytosis assay (AICP)
  - Bone marrow derived macrophage generation
  - CLL patient cell co-culture
  - CLL patient cell chemotoxicity stain
  - ELISA
  - Immunofluorescent microscopy
  - Immune phenotyping
  - Immunohistochemical staining of murine spleen
  - *In vivo* experiments
  - Knockdown cell production
  - Metabolomics
  - Maturation staining of primary murine macrophages
  - Phosphoproteomics
  - Primary human macrophages
  - Proteomics
  - SeaHorse analysis
  - Viability stain
  - Western Blot analysis
- QUANTIFICATION AND STATISTICAL ANALYSIS
  - Statistical analysis
  - Proteomic and phosphoproteomic analysis

SUPPLEMENTAL INFORMATION

Supplemental information can be found online at https://doi.org/10.1016/j.xcrm.2024.101830.

Received: February 29, 2024
Revised: August 27, 2024
Accepted: October 25, 2024
Published: November 26, 2024

REFERENCES

1. Hanahan, D., and Weinberg, R.A. (2011). Hallmarks of cancer: the next generation. Cell *144*, 646–674. https://doi.org/10.1016/j.cell.2011.02.013.
2. Beielstein, A.C., and Pallasch, C.P. (2019). Tumor Metabolism as a Regulator of Tumor-Host Interactions in the B-Cell Lymphoma Microenvironment-Fueling Progression and Novel Brakes for Therapy. Int. J. Mol. Sci. *20*, 4158.
3. Cassetta, L., and Pollard, J.W. (2018). Targeting macrophages: Therapeutic approaches in cancer. Nat. Rev. Drug Discov. *17*, 887–904.
4. Yang, L., and Zhang, Y. (2017). Tumor-associated macrophages: from basic research to clinical application. J. Hematol. Oncol. *10*, 58.
5. Pallasch, C.P., Leskov, I., Braun, C.J., Vorholt, D., Drake, A., Soto-Feliciano, Y.M., Bent, E.H., Schwamb, J., Iliopoulou, B., Kutsch, N., et al. (2014). Sensitizing protective tumor microenvironments to antibody-mediated therapy. Cell *156*, 590–602. https://doi.org/10.1016/j.cell.2013.12.041.
6. Biswas, S.K., Gangi, L., Paul, S., Schioppa, T., Saccani, A., Sironi, M., Bottazzi, B., Doni, A., Vincenzo, B., Pasqualini, F., et al. (2006). A distinct and unique transcriptional program expressed by tumor-associated

CellPress
OPEN ACCESS

macrophages (defective NF-κB and enhanced IRF-3/STAT1 activation). Blood *107*, 2112–2122.

7. Gallina, G., Dolcetti, L., Serafini, P., De Santo, C., Marigo, I., Colombo, M.P., Basso, G., Brombacher, F., Borrello, I., Zanovello, P., et al. (2006). Tumors induce a subset of inflammatory monocytes with immunosuppressive activity on CD8+ T cells. J. Clin. Invest. *116*, 2777–2790.
8. Jha, A.K., Huang, S.C.C., Sergushichev, A., Lampropoulou, V., Ivanova, Y., Loginicheva, E., Chmielewski, K., Stewart, K.M., Ashall, J., Everts, B., et al. (2015). Network integration of parallel metabolic and transcriptional data reveals metabolic modules that regulate macrophage polarization. Immunity *42*, 419–430.
9. Diskin, C., and Pålsson-McDermott, E.M. (2018). Metabolic modulation in macrophage effector function. Front. Immunol. *9*, 270. https://doi.org/10.3389/fimmu.2018.00270.
10. de-Brito N, M., Duncan-Moretti, J., da-Costa H, C., Saldanha-Gama, R., Paula-Neto, H.A., Dorighello, G.G., Simões, R.L., and Barja-Fidalgo, C. (2020). Aerobic glycolysis is a metabolic requirement to maintain the M2-like polarization of tumor-associated macrophages. Biochim. Biophys. Acta. Mol. Cell Res. *1867*, 118604.
11. Hörhold, F., Eisel, D., Oswald, M., Kolte, A., Röll, D., Osen, W., Eichmüller, S.B., and König, R. (2020). Reprogramming of macrophages employing gene regulatory and metabolic network models. PLoS Comput. Biol. *16*, 683–696.
12. Simões, R.L., De-Brito, N.M., Cunha-Costa, H., Morandi, V., Fierro, I.M., Roitt, I.M., and Barja-Fidalgo, C. (2017). Lipoxin A4 selectively programs the profile of M2 tumor-associated macrophages which favour control of tumor progression. Int. J. Cancer *140*, 346–357.
13. Elf, S., Lin, R., Xia, S., Pan, Y., Shan, C., Wu, S., Lonial, S., Gaddh, M., Arellano, M.L., Khoury, H.J., et al. (2017). Targeting 6-phosphogluconate dehydrogenase in the oxidative PPP sensitizes leukemia cells to antimalarial agent dihydroartemisinin. Oncogene *36*, 254–262.
14. Solovjeva, O.N., and Kochetov, G.A. (1999). Inhibition of transketolase by p-hydroxyphenylpyruvate. FEBS Lett. *462*, 246–248.
15. Weigert, A., Strack, E., Snodgrass, R.G., and Brüne, B. (2018). mPGES-1 and ALOX5/-15 in tumor-associated macrophages. Cancer Metastasis Rev. *37*, 317–334.
16. Mylka, V., Deckers, J., Ratman, D., De Cauwer, L., Thommis, J., De Rycke, R., Impens, F., Libert, C., Tavernier, J., Vanden Berghe, W., et al. (2018). The autophagy receptor SQSTM1/p62 mediates anti-inflammatory actions of the selective NR3C1/glucocorticoid receptor modulator compound A (CpdA) in macrophages. Autophagy *14*, 2049–2064.
17. Coles, L.S., Diamond, P., Occhiodoro, F., Vadas, M.A., and Shannon, M.F. (1996). Cold shock domain proteins repress transcription from the GM-CSF promoter. Nucleic Acids Res. *24*, 2311–2317.
18. Bartish, M., Tong, D., Pan, Y., Wallerius, M., Liu, H., Ristau, J., de Souza Ferreira, S., Wallmann, T., van Hoef, V., Masvidal, L., et al. (2020). MNK2 governs the macrophage antiinflammatory phenotype. Proc. Natl. Acad. Sci. USA *117*, 27556–27565.
19. Chang, Y.-T., Peng, H.-Y., Hu, C.-M., Huang, S.-C., Tien, S.-C., and Jeng, Y.-M. (2020). Pancreatic cancer-derived small extracellular vesical Ezrin regulates macrophage polarization and promotes metastasis. Am. J. Cancer Res. *10*, 12–37.
20. Iwata, H., Goettsch, C., Sharma, A., Ricchiuto, P., Goh, W.W.B., Halu, A., Yamada, I., Yoshida, H., Hara, T., Wei, M., et al. (2016). PARP9 and PARP14 cross-regulate macrophage activation via STAT1 ADP-ribosylation. Nat. Commun. *7*, 12849.
21. Zhang, Y., Li, X., Wang, C., Zhang, M., Yang, H., and Lv, K. (2020). lncRNA AK085865 Promotes Macrophage M2 Polarization in CVB3-Induced VM by Regulating ILF2-ILF3 Complex-Mediated miRNA-192 Biogenesis. Mol. Ther. Nucleic Acids *21*, 441–451.
22. Beekhof, R., Alphen, C., Henneman, A.A., Knol, J.C., Pham, T.V., Rolfs, F., Labots, M., Henneberry, E., Ys Le Large, T., de Haas, R.R., et al. (2019). INKA , an integrative data analysis pipeline for phosphoproteomic inference of active kinases. Mol. Syst. Biol. *15*, e8250. https://doi.org/10.15252/msb.20188250.
23. Poh, A.R., O'Donoghue, R.J.J., and Ernst, M. (2015). Hematopoietic cell kinase (HCK) as a therapeutic target in immune and cancer cells. Oncotarget *6*, 15752–15771.
24. Dwyer, A.R., Mouchemore, K.A., Steer, J.H., Sunderland, A.J., Sampaio, N.G., Greenland, E.L., Joyce, D.A., and Pixley, F.J. (2016). Src family kinase expression and subcellular localization in macrophages: implications for their role in CSF-1-induced macrophage migration. J. Leukoc. Biol. *100*, 163–175.
25. Foucher, E.D., Blanchard, S., Preisser, L., Garo, E., Ifrah, N., Guardiola, P., Delneste, Y., and Jeannin, P. (2013). IL-34 Induces the Differentiation of Human Monocytes into Immunosuppressive Macrophages. Antagonistic Effects of GM-CSF and IFNγ. PLoS One *8*, e56045. https://doi.org/10.1371/journal.pone.0056045.
26. Basile, J.R., Afkhami, T., and Gutkind, J.S. (2005). Semaphorin 4D/Plexin-B1 Induces Endothelial Cell Migration through the Activation of PYK2, Src, and the Phosphatidylinositol 3-Kinase-Akt Pathway. Mol. Cell Biol. *25*, 6889–6898.
27. O'Neill, L.A.J., and Artyomov, M.N. (2019). Itaconate: the poster child of metabolic reprogramming in macrophage function. Nat. Rev. Immunol. *19*, 273–281.
28. Ma, J., Wei, K., Liu, J., Tang, K., Zhang, H., Zhu, L., Chen, J., Li, F., Xu, P., Chen, J., et al. (2020). Glycogen metabolism regulates macrophage-mediated acute inflammatory responses. Nat. Commun. *11*, 1769. https://doi.org/10.1038/s41467-020-15636-8.
29. Taniguchi, T., Ogasawara, K., Takaoka, A., and Tanaka, N. (2001). IRF family of transcription factors as regulators of host defense. Annu. Rev. Immunol. *19*, 623–655.
30. Tallam, A., Perumal, T.M., Antony, P.M., Jäger, C., Fritz, J.V., Vallar, L., Balling, R., Del Sol, A., and Michelucci, A. (2016). Gene Regulatory Network Inference of Immunoresponsive Gene 1 (IRG1) Identifies Interferon Regulatory Factor 1 (IRF1) as Its Transcriptional Regulator in Mammalian Macrophages. PLoS One *11*, e0149050. https://doi.org/10.1371/journal.pone.0149050.
31. Fernández-García, M., Mesquita, I., Ferreira, C., Araújo, M., Saha, B., Rey-Stolle, M.F., García, A., Silvestre, R., and Barbas, C. (2023). Leishmania donovani Induces Multiple Dynamic Responses in the Metabolome Associated with Amastigote Differentiation and Maturation Inside the Human Macrophage. J. Proteome Res. *22*, 2256–2270.
32. Lauterbach, M.A., Hanke, J.E., Serefidou, M., Mangan, M.S.J., Kolbe, C.C., Hess, T., Rothe, M., Kaiser, R., Hoss, F., Gehlen, J., et al. (2019). Toll-like Receptor Signaling Rewires Macrophage Metabolism and Promotes Histone Acetylation via ATP-Citrate Lyase. Immunity *51*, 997–1011.e7.
33. Burger, J.A., Tsukada, N., Burger, M., Zvaifler, N.J., Dell'Aquila, M., and Kipps, T.J. (2000). Blood-derived nurse-like cells protect chronic lymphocytic leukemia B cells from spontaneous apoptosis through stromal cell-derived factor-1. Blood *96*, 2655–2663.
34. Pietras, E.M., Reynaud, D., Kang, Y.A., Carlin, D., Calero-Nieto, F.J., Leavitt, A.D., Stuart, J.M., Göttgens, B., and Passegué, E. (2015). Functionally Distinct Subsets of Lineage-Biased Multipotent Progenitors Control Blood Production in Normal and Regenerative Conditions. Cell Stem Cell *17*, 35–46.
35. Sommerkamp, P., Romero-Mulero, M.C., Narr, A., Ladel, L., Hustin, L., Schönberger, K., Renders, S., Altamura, S., Zeisberger, P., Jäcklein, K., et al. (2021). Mouse multipotent progenitor 5 cells are located at the interphase between hematopoietic stem and progenitor cells. Blood *137*, 3218–3224.
36. Leskov, I., Pallasch, C.P., Drake, A., Iliopoulou, B.P., Souza, A., Shen, C.H., Schweighofer, C.D., Abruzzo, L., Frenzel, L.P., Wendtner, C.M., et al. (2013). Rapid generation of human B-cell lymphomas via combined expression of Myc and Bcl2 and their use as a preclinical model for biological therapies. Oncogene *32*, 1066–1072.

37. Izquierdo, E., Cuevas, V.D., Fernández-Arroyo, S., Riera-Borrull, M., Orta-Zavalza, E., Joven, J., Rial, E., Corbi, A.L., and Escribese, M.M. (2015). Reshaping of Human Macrophage Polarization through Modulation of Glucose Catabolic Pathways. J. Immunol. *195*, 2442–2451.

38. He, D., Mao, Q., Jia, J., Wang, Z., Liu, Y., Liu, T., Luo, B., and Zhang, Z. (2021). Pentose Phosphate Pathway Regulates Tolerogenic Apoptotic Cell Clearance and Immune Tolerance. Front. Immunol. *12*, 797091. https://doi.org/10.3389/FIMMU.2021.797091.

39. Nakamizo, S., Sugiura, Y., Ishida, Y., Ueki, Y., Yonekura, S., Tanizaki, H., Date, H., Yoshizawa, A., Murata, T., Minatoya, K., et al. (2023). Activation of the pentose phosphate pathway in macrophages is crucial for granuloma formation in sarcoidosis. J. Clin. Invest. *133*, e171088. https://doi.org/10.1172/JCI171088.

40. Caldwell, C.C., Kojima, H., Lukashev, D., Armstrong, J., Farber, M., Apasov, S.G., and Sitkovsky, M.V. (2001). Differential Effects of Physiologically Relevant Hypoxic Conditions on T Lymphocyte Development and Effector Functions. J. Immunol. *167*, 6140–6149.

41. Spencer, J.A., Ferraro, F., Roussakis, E., Klein, A., Wu, J., Runnels, J.M., Zaher, W., Mortensen, L.J., Alt, C., Turcotte, R., et al. (2014). Direct measurement of local oxygen concentration in the bone marrow of live animals. Nature *508*, 269–273.

42. Lin, R., Elf, S., Shan, C., Kang, H.B., Ji, Q., Zhou, L., Hitosugi, T., Zhang, L., Zhang, S., Seo, J.H., et al. (2015). 6-Phosphogluconate dehydrogenase links oxidative PPP, lipogenesis and tumour growth by inhibiting LKB1–AMPK signalling. Nat. Cell Biol. *17*, 1484–1496.

43. Stincone, A., Prigione, A., Cramer, T., Wamelink, M.M.C., Campbell, K., Cheung, E., Olin-Sandoval, V., Grüning, N.M., Krüger, A., Tauqeer Alam, M., et al. (2015). The return of metabolism: Biochemistry and physiology of the pentose phosphate pathway. Biol. Rev. *90*, 927–963.

44. Chen, H., Wu, D., Bao, L., Yin, T., Lei, D., Yu, J., and Tong, X. (2019). 6PGD inhibition sensitizes hepatocellular carcinoma to chemotherapy via AMPK activation and metabolic reprogramming. Biomed. Pharmacother. *111*, 1353–1358.

45. Liu, H., Huang, D., McArthur, D.L., Boros, L.G., Nissen, N., and Heaney, A.P. (2010). Fructose induces transketolase flux to promote pancreatic cancer growth. Cancer Res. *70*, 6368–6376.

46. Zheng, W., Feng, Q., Liu, J., Guo, Y., Gao, L., Li, R., Xu, M., Yan, G., Yin, Z., Zhang, S., et al. (2017). Inhibition of 6-phosphogluconate dehydrogenase reverses cisplatin resistance in ovarian and lung cancer. Front. Pharmacol. *8*, 421. https://doi.org/10.3389/fphar.2017.00421.

47. Bhanot, H., Weisberg, E.L., Reddy, M.M., Nonami, A., Neuberg, D., Stone, R.M., Podar, K., Salgia, R., Griffin, J.D., and Sattler, M. (2017). Acute myeloid leukemia cells require 6-phosphogluconate dehydrogenase for cell growth and NADPH-dependent metabolic reprogramming. Oncotarget *8*, 67639–67650.

48. Xu, I.M.J., Lai, R.K.H., Lin, S.H., Tse, A.P.W., Chiu, D.K.C., Koh, H.Y., Law, C.T., Wong, C.M., Cai, Z., Wong, C.C.L., and Ng, I.O.L. (2016). Transketolase counteracts oxidative stress to drive cancer development. Proc. Natl. Acad. Sci. USA *113*, E725–E734.

49. Chan, B., VanderLaan, P.A., and Sukhatme, V.P. (2013). 6-Phosphogluconate dehydrogenase regulates tumor cell migration in vitro by regulating receptor tyrosine kinase c-Met. Biochem. Biophys. Res. Commun. *439*, 247–251.

50. Zhang, H., Zhang, H., Wang, S., Ni, Z., and Wang, T. (2019). 1-Hydroxy-8-methoxy-anthraquinon reverses cisplatin resistance by inhibiting 6PGD in cancer cells. Open Life Sci. *14*, 454–461.

51. Gao, F., Liu, W., Guo, Q., Bai, Y., Yang, H., and Chen, H. (2017). Physcion blocks cell cycle and induces apoptosis in human B cell precursor acute lymphoblastic leukemia cells by downregulating HOXA5. Biomed. Pharmacother. *94*, 850–857.

52. Budihardjo, I.I., Walker, D.L., Svingen, P.A., Buckwalter, C.A., Desnoyers, S., Eckdahl, S., Shah, G.M., Poirier, G.G., Reid, J.M., Ames, M.M., and Kaufmann, S.H. (1998). 6-Aminonicotinamide sensitizes human tumor cell lines to cisplatin. Clin. Cancer Res. *4*, 117–130.

53. Daneshmandi, S., Cassel, T., Lin, P., Higashi, R.M., Wulf, G.M., Boussiotis, V.A., Fan, T.W.M., and Seth, P. (2021). Blockade of 6-phosphogluconate dehydrogenase generates CD8+ effector T cells with enhanced anti-tumor function. Cell Rep. *34*, 108831. https://doi.org/10.1016/j.celrep.2021.108831.

54. Lossos, C., Liu, Y., Kolb, K.E., Christie, A.L., Van Scoyk, A., Prakadan, S.M., Shigemori, K., Stevenson, K.E., Morrow, S., Plana, O.D., et al. (2019). Mechanisms of lymphoma clearance induced by high-dose alkylating agents. Cancer Discov. *9*, 944–961. https://doi.org/10.1158/2159-8290.CD-18-1393.

55. Barbarino, V., Henschke, S., Blakemore, S.J., Izquierdo, E., Michalik, M., Nickel, N., Möllenkotte, I., Vorholt, D., Müller, L., Brinker, R., et al. (2020). Macrophage-Mediated Antibody Dependent Effector Function in Aggressive B-Cell Lymphoma Treatment is Enhanced by Ibrutinib via Inhibition of JAK2. Cancers *12*, 2303.

56. Izquierdo, E., Vorholt, D., Blakemore, S., Sackey, B., Nolte, J.L., Barbarino, V., Schmitz, J., Nickel, N., Bachurski, D., Lobastova, L., et al. (2022). Extracellular vesicles and PD-L1 suppress macrophages, inducing therapy resistance in TP53-deficient B-cell malignancies. Blood *139*, 3617–3629.

57. Rodríguez-Prados, J.-C., Través, P.G., Cuenca, J., Rico, D., Aragonés, J., Martín-Sanz, P., Cascante, M., and Boscá, L. (2010). Substrate Fate in Activated Macrophages: A Comparison between Innate, Classic, and Alternative Activation. J. Immunol. *185*, 605–614.

58. Bronte, V., Brandau, S., Chen, S.H., Colombo, M.P., Frey, A.B., Greten, T.F., Mandruzzato, S., Murray, P.J., Ochoa, A., Ostrand-Rosenberg, S., et al. (2016). Recommendations for myeloid-derived suppressor cell nomenclature and characterization standards. Nat. Commun. *7*, 12150.

59. Solinas, G., Schiarea, S., Liguori, M., Fabbri, M., Pesce, S., Zammataro, L., Pasqualini, F., Nebuloni, M., Chiabrando, C., Mantovani, A., and Allavena, P. (2010). Tumor-Conditioned Macrophages Secrete Migration-Stimulating Factor: A New Marker for M2-Polarization, Influencing Tumor Cell Motility. J. Immunol. *185*, 642–652.

60. Hesketh, M., Sahin, K.B., West, Z.E., and Murray, R.Z. (2017). Macrophage Phenotypes Regulate Scar Formation and Chronic Wound Healing. Int. J. Mol. Sci. *18*, 1545.

61. Woo, M.-S., Yang, J., Beltran, C., and Cho, S. (2016). Cell Surface CD36 Protein in Monocyte/Macrophage Contributes to Phagocytosis during the Resolution Phase of Ischemic Stroke in Mice. J. Biol. Chem. *291*, 23654–23661.

62. Kwiecień, I., Polubiec-Kownacka, M., Dziedzic, D., Wołosz, D., Rzepecki, P., and Domagała-Kulawik, J. (2019). CD163 and CCR7 as markers for macrophage polarization in lung cancer microenvironment. Cent. Eur. J. Immunol. *44*, 395–402.

63. Jablonski, K.A., Amici, S.A., Webb, L.M., Ruiz-Rosado, J.d.D., Popovich, P.G., Partida-Sanchez, S., and Guerau-de-Arellano, M. (2015). Novel markers to delineate murine M1 and M2 macrophages. PLoS One *10*, e0145342. https://doi.org/10.1371/journal.pone.0145342.

64. Otero, K., Turnbull, I.R., Poliani, P.L., Vermi, W., Cerutti, E., Aoshi, T., Tassi, I., Takai, T., Stanley, S.L., Miller, M., et al. (2009). Macrophage colony-stimulating factor induces the proliferation and survival of macrophages via a pathway involving DAP12 and β-catenin. Nat. Immunol. *10*, 734–743.

65. Lin, C.C., Hsiao, L.D., Cho, R.L., and Yang, C.M. (2019). Carbon monoxide releasing molecule-2-upregulated ROS-dependent heme oxygenase-1 axis suppresses lipopolysaccharide-induced airway inflammation. Int. J. Mol. Sci. *20*, 3157. https://doi.org/10.3390/ijms20133157.

66. Jamal Uddin, M., Joe, Y., Kim, S.K., Oh Jeong, S., Ryter, S.W., Pae, H.O., and Chung, H.T. (2016). IRG1 induced by heme oxygenase-1/carbon monoxide inhibits LPS-mediated sepsis and pro-inflammatory cytokine production. Cell. Mol. Immunol. *13*, 170–179.

67. Poh, A.R., Love, C.G., Masson, F., Preaudet, A., Tsui, C., Whitehead, L., Monard, S., Khakham, Y., Burstroem, L., Lessene, G., et al. (2017). Inhibition of Hematopoietic Cell Kinase Activity Suppresses Myeloid Cell-Mediated Colon Cancer Progression. Cancer Cell *31*, 563–575.e5.

68. Fleetwood, A.J., Lawrence, T., Hamilton, J.A., and Cook, A.D. (2007). Granulocyte-Macrophage Colony-Stimulating Factor (CSF) and Macrophage CSF-Dependent Macrophage Phenotypes Display Differences in Cytokine Profiles and Transcription Factor Activities: Implications for CSF Blockade in Inflammation. J. Immunol. *178*, 5245–5252.

69. Stanley, E.R., and Chitu, V. (2014). CSF-1 receptor signaling in myeloid cells. Cold Spring Harb. Perspect. Biol. *6*, a021857. https://doi.org/10.1101/cshperspect.a021857.

70. Cannarile, M.A., Weisser, M., Jacob, W., Jegg, A.M., Ries, C.H., and Rüttinger, D. (2017). Colony-stimulating factor 1 receptor (CSF1R) inhibitors in cancer therapy. J. Immunother. Cancer *5*, 53. https://doi.org/10.1186/s40425-017-0257-y.

71. Lazarowski, E.R., and Harden, T.K. (2015). UDP-sugars as extracellular signaling molecules: Cellular and physiologic consequences of P2Y14 receptor activation. Mol. Pharmacol. *88*, 151–160.

72. Tallam, A., Perumal, T.M., Antony, P.M., Jäger, C., Fritz, J.V., Vallar, L., Balling, R., Del Sol, A., and Michelucci, A. (2016). Gene Regulatory Network Inference of Immunoresponsive Gene 1 (IRG1) Identifies Interferon Regulatory Factor 1 (IRF1) as Its Transcriptional Regulator in Mammalian Macrophages. PLoS One *11*, e0149050. https://doi.org/10.1371/journal.pone.0149050.

73. Lampropoulou, V., Sergushichev, A., Bambouskova, M., Nair, S., Vincent, E.E., Loginicheva, E., Cervantes-Barragan, L., Ma, X., Huang, S.C.C., Griss, T., et al. (2016). Itaconate Links Inhibition of Succinate Dehydrogenase with Macrophage Metabolic Remodeling and Regulation of Inflammation. Cell Metab. *24*, 158–166.

74. Mills, E.L., Ryan, D.G., Prag, H.A., Dikovskaya, D., Menon, D., Zaslona, Z., Jedrychowski, M.P., Costa, A.S.H., Higgins, M., Hams, E., et al. (2018). Itaconate is an anti-inflammatory metabolite that activates Nrf2 via alkylation of KEAP1. Nature *556*, 113–117.

75. Weiss, J.M., Davies, L.C., Karwan, M., Ileva, L., Ozaki, M.K., Cheng, R.Y., Ridnour, L.A., Annunziata, C.M., Wink, D.A., and McVicar, D.W. (2018). Itaconic acid mediates crosstalk between macrophage metabolism and peritoneal tumors. J. Clin. Invest. *128*, 3794–3805.

76. Nath, N., and Kashfi, K. (2020). Tumor associated macrophages and 'NO. Biochem. Pharmacol. *176*, 113899. https://doi.org/10.1016/j.bcp.2020.113899.

77. Biswas, S.K., Gangi, L., Paul, S., Schioppa, T., Saccani, A., Sironi, M., Bottazzi, B., Doni, A., Vincenzo, B., Pasqualini, F., et al. (2006). A distinct and unique transcriptional program expressed by tumor-associated macrophages (defective NF-κB and enhanced IRF-3/STAT1 activation). Blood *107*, 2112–2122.

78. Van Ginderachter, J.A., Movahedi, K., Hassanzadeh Ghassabeh, G., Meerschaut, S., Beschin, A., Raes, G., and De Baetselier, P. (2006). Classical and alternative activation of mononuclear phagocytes: Picking the best of both worlds for tumor promotion. Immunobiology *211*, 487–501.

79. Zenke, K., Muroi, M., and Tanamoto, K.I. (2018). IRF1 supports DNA binding of STAT1 by promoting its phosphorylation. Immunol. Cell Biol. *96*, 1095–1103.

STAR★METHODS

KEY RESOURCES TABLE

REAGENT or RESOURCE	SOURCE	IDENTIFIER
Antibodies		
Rabbit polyclonal anti-β-actin	BioLegend	Cat#622101; RRID:AB_315945
Sheep polyclonal anti-Arg1	RnD Systems	Cat#IC5868P
Mouse monoclonal anti-CCR7 (CD197)	BioLegend	Cat#353213; RRID:AB_10915474
Rat monoclonal anti-CD11b	BioLegend	Cat#101226; RRID:AB_830642
Rat monoclonal anti-CD115 (CSF-1R)	BioLegend	Cat#135523; RRID:AB_2566459
Rat monoclonal anti-CD16/32	BioLegend	Cat#156607; RRID:AB_2800705
Rat monoclonal anti-CD19	Thermo Fisher Scientific	Cat#14-0194-80; RRID:AB_2637170
Mouse monoclonal anti-CD200R	BioLegend	Cat#329305; RRID:AB_2074201
Mouse monoclonal anti-CD206	BD Biosciences	Cat#551135; RRID:AB_394065
Rat monoclonal anti-CD38	BioLegend	Cat#102717; RRID:AB_2072892
Mouse monoclonal anti- CD64	BioLegend	Cat#305025; RRID:AB_2561587
Rabbit polyclonal anti-CD68	Abcam	Cat#ab125212; RRID:AB_10975465
Rat monoclonal anti-CD68	BioLegend	Cat#137017; RRID:AB_2562949
Mouse monoclonal anti-CD80	BD Biosciences	Cat#557227; RRID:AB_396606
Rat monoclonal anti-CD86	Miltenyi Biotec	Cat#130-123-724,;RRID:AB_2889634
Mouse monoclonal anti-CX3CR1	BioLegend	Cat#149007; RRID:AB_2564491
Rat monoclonal anti-EGR-2	Thermo Fisher Scientific	Cat#17-6691-82; RRID:AB_11151502
Rat monoclonal anti-F4/80	BioLegend	Cat#123110; RRID:AB_893486
Rat monoclonal anti-F4/80	BioLegend	Cat#123124; RRID:AB_893475
Human cell line monoclonal anti-F4/80	Miltenyi Biotec	Cat #130-102-327; RRID:AB_2651701
Mouse monoclonal anti-human HLA-DR (MHC II)	BioLegend	Cat#307604; RRID:AB_314682
Mouse Histofine Simple Stain Mouse MAX-PO	Nichirei Biosciences	Cat#414341F; RRID:AB_2819094
Rat monoclonal anti-IL-10	BioLegend	Cat#505025; RRID:AB_11149682
Mouse monoclonal anti-iNOS	Novus Biologicals	Cat#NBP2-22119; RRID:AB_2905500
Rabbit monoclonal anti-IRF1 (D5E4)	Cell Signaling Technology	Cat#8478; RRID:AB_10949108
Rabbit polyclonal anti-IRG1	Cell Signaling Technology	Cat#17805; RRID:AB_3064865
Rat monoclonal anti-Ly6c	BioLegend	Cat#128011; RRID:AB_1659242
Rabbit polyclonal anti-P2RY14	LSBio	Cat#LS-C409714
Rabbit monoclonal anti-PD-1 (D7D5W)	Cell Signaling Technology	Cat#84651; RRID:AB_2800041
Rabbit polyclonal anti-PD-L1	Thermo Fisher Scientific	Cat#PA5-20343; RRID:AB_11153819
Mouse monoclonal anti-phosphogluconate dehydrogenase (G-2) (6PGD)	Santa Cruz Biotechnology, INC.	Cat#sc-398977; RRID:AB_2827766
Rabbit monoclonal anti- Protein-tyrosine kinase 2-beta (PYK2)	Abcam	Cat#ab32571; RRID:AB_777566
Rabbit polyclonal anti-SIRP1a	SIGMA-ALDRICH	Cat#SAB2102154; RRID:AB_10605073
Rabbit monoclonal anti-STAT1	Cell Signaling Technology	Cat#80916; RRID:AB_2799965
Mouse monoclonal anti-TGF-beta1	BioLegend	Cat#349706; RRID:AB_10680787
Rabbit polyclonal anti-transketolase (TKT)	Biorbyt Ltd.	Cat#orb247362
Rabbit polyclonal anti-UGP2	Thermo Fisher Scientific	Cat#PA5-27760; RRID:AB_2545236
Bacterial and virus strains		
5-alpha Competent E. coli	New England Biolabs Inc.	Cat#C2987H

(*Continued on next page*)

Continued

REAGENT or RESOURCE	SOURCE	IDENTIFIER
Chemicals, peptides, and recombinant proteins		
1-hydroxy-8-methoxy-anthraquinone (S3)	SIGMA-ALDRICH	Cat#R164046
1-hydroxy-8-methoxy-anthraquinone (S3)	SAGECHEM LIMITED	Cat#S474625
1,4-Dithiothreit	CARL ROTH	Cat#6908.2
2-deoxy-D-glucose	SIGMA-ALDRICH	Cat#D8357
6-aminonicotinamide	SIGMA-ALDRICH	Cat#A0630; CAS: 329-89-5
6-phosphogluconolactone	SIGMA-ALDRICH	Cat#P7877
Acetonitril	SIGMA-ALDRICH	Cat#34851
Alemtuzumab	MabCampath	NDC code 58468-0357-3
Alpha-ketoglutaric acid	SIGMA-ALDRICH	Cat#61234
Adenosine-5′-diphosphate	SIGMA-ALDRICH	Cat#01905
Adenosine-5′-triphosphate	SIGMA-ALDRICH	Cat#A2383
β-nicotinamide adenine dinucleotide (NAD)	SIGMA-ALDRICH	Cat#N1511
Bendamustine hydrochloride hydrate	SIGMA-ALDRICH	Cat#B5437
BML-275 hydrochloride	SIGMA-ALDRICH	Cat#ADVH7F38323F
Citric acid	SIGMA-ALDRICH	Cat#94676
DAPI (4′,6-Diamidino-2-phenylindoldihydrochlorid)	SIGMA-ALDRICH	Cat#D9542
Daratumumab	Janssen-Cilag International N.V.	EMEA/H/C/004077
D-erythrose-4-phosphate sodium	SIGMA-ALDRICH	Cat#E0377; CAS: 103302-15-4
D-fructose 1,6-biphosphate	SIGMA-ALDRICH	Cat#F6803
D-fructose-6-phosphate disodium salt hydrate	SIGMA-ALDRICH	Cat#F3627; CAS: 26177-86-6
D-glucose-6-phosphate sodium salt	SIGMA-ALDRICH	Cat#G7879
DL-glyceraldehyde-3-phosphate	SIGMA-ALDRICH	Cat#G5251
dNTP mix	Thermo Fisher Scientific	Cat#R0192
D-ribose-5-phosphate disodium salt hydrate	SIGMA-ALDRICH	Cat#R7750; CAS: 18265-46-8
D-ribulose-5-phosphate sodium salt	SIGMA-ALDRICH	Cat#R9875
D-sedoheptulose-7-phosphate lithium salt	SIGMA-ALDRICH	Cat#78832
D-xylulose-5-phosphate lithium salt	SIGMA-ALDRICH	Cat#78963
Dihydronicotinamide adenine dinucleotide (NADH)	SIGMA-ALDRICH	Cat#47861
DL-glyceraldehyde-3-phosphate	SIGMA-ALDRICH	Cat#G5251; CAS: 591-59-3
Glacial acetic acid	VWR International	Cat#1005706
Glutathione (reduced)	SIGMA-ALDRICH	Cat#PHR1359
Glutathione–glycine-$^{13}C_2$,^{15}N trifluor	SIGMA	Cat#683620
Itaconic acid	SIGMA-ALDRICH	Cat#93598
L-malic acid	SIGMA-ALDRICH	Cat#09172
L-succinic acid	SIGMA-ALDRICH	Cat#46937
Lactic acid	SIGMA-ALDRICH	Cat#46937
LentiX GoStix Plus	Takara Bio Inc.	Cat#631280
Lymphoprep	STEMCELL Technologies	Cat#07801
M-CSF, recombinant human	Thermo Fisher Scientific	Cat#PHC9501
M-CSF, recombinant mouse	Thermo Fisher Scientific	Cat#PMC2044
Microbeads CD14 human	Miltenyi Biotec	Cat#130-050-201; RRID:AB_2665482
Nicotinamid-adenin-dinucleotid-phosphate (NADP)	SIGMA-ALDRICH	Cat#481972

(*Continued on next page*)

Continued		
REAGENT or RESOURCE	SOURCE	IDENTIFIER
Obinutuzumab	Roche Registration Limited	EMEA/H/C/002799
Oligomycin	SIGMA-ALDRICH	Cat#O4876
Oxythiamine chloride hydrochloride	SIGMA-ALDRICH	Cat#O4000
PageRuler prestained NIR Protein ladder	Thermo Fisher Scientific	Cat#26635
P-hydroxyphenylpyruvate 98%	SIGMA-ALDRICH	Cat#114286; CAS: 156-39-8
Physcion	SIGMA-ALDRICH	Cat#17797; CAS: 521-61-9
Pyruvic acid	SIGMA-ALDRICH	Cat#19215
Restriction Endonuclease EcoR1	New England Biolabs Inc.	Cat#R0101L
Restriction Endonuclease Xho1	New England Biolabs Inc.	Cat#R0146L
Succinic acid D6	SIGMA-ALDRICH	Cat#488356
Vent polymerase	New England Biolabs Inc.	Cat#M0245S
Critical commercial assays		
7AAD viability staining eBioscience	Thermo Fisher Scientific	Cat#A1310
BCA Protein Assay Kit	Thermo Fisher Scientific	Cat#23227
CellTiter-Glo Luminescent Cell Viability Assay Kit	Promega	Cat#G7570
Fix & Perm Cell Permeabilization Kit	Thermo Fisher Scientific	Cat#GAS003
Human IL-6 ELISA MAX Standard Set	Thermo Fisher Scientific	Cat#430501
Human IL-10 ELISA MAX Standard Set	Thermo Fisher Scientific	Cat#430601
I-Blue Midi Plasmid Kit	IBI SCIENTIFIC	Cat#IB47180
Mouse IL-6 ELISA MAX Standard Set	BioLegend	Cat#431301; RRID:AB_2883997
Mouse IL-10 ELISA MAX Standard Set	BioLegend	Cat#431411
Odyssey Blocking Buffer	LI-COR	Cat#927-40000
Phosphopeptide Enrichment Kit	Thermo Fisher Scientific	Cat#A32993
QIA quick PCR Purification Kit	QIAGEN	Cat#28104
REVERT Total protein stain	LI-COR Biotech.	Cat#926-11011
SeaHorse XF Base Medium	Agilent Technologies, Inc.	Cat#103334-100
SeaHorse XFe96 FluxPak	Agilent Technologies, Inc.	Cat#102416-100
Zombie NIR Fixable Viability Kit	BioLegend	Cat#423105
Deposited data		
Affinity-based mass spectrometry performed with 5680 proteins (Proteomic analysis)	This paper	Accession number PXD042428, https://www.ebi.ac.uk/pride/
Affinity-based mass spectrometry performed with 19383 protein-sites (Phospho-proteomic analysis)	This paper	Accession number PXD042428, https://www.ebi.ac.uk/pride/
LC MS/MS analysis (Metabolomic analysis)	This paper	Accession number ST003516, https://www.metabolomicsworkbench.org/
Murine database for phosphopeptides	PhosphoSitePlus	https://www.phosphosite.org/staticDownloads
Experimental models: Cell lines		
Human: HEK293T-CAF40-null	DSMZ	Cat#ACC-872; RRID:CVCL_A5EE
Human: THP-1	DSMZ	Cat#ACC-16; RRID:CVCL_0006
Humanized mouse cells: hMB, strain 102		Leskov et al.[36]
Mouse: J774A.1	ATCC	Cat#TIB-67; RRID:CVCL_0358
Human: L-929	DSMZ	Cat#ACC-2; RRID:CVCL_0462

(*Continued on next page*)

Continued

REAGENT or RESOURCE	SOURCE	IDENTIFIER
Experimental models: Organisms/strains		
Mouse: C57BL/6J	Jackson laboratory	Cat#000664; RRID:IMSR_JAX:000664
Mouse: Wild-type NOD.Cg-Prkdc^{scid} Il2rg^{tm1Wjl}/SzJ (NSG)	Jackson laboratory	Cat#005557/NSG; RRID:IMSR_JAX:005557
Mouse: C57BL/6NJ-Acod1$^{em1(IMPC)J}$/J	Jackson laboratory	Cat#029340; RRID: IMSR_JAX:02 9340
Oligonucleotides		
6PGD_1	SIGMA-ALDRICH	Oligo#8810932277-000060
6PGD_2	SIGMA-ALDRICH	Oligo#8810932277-000070
TKT_1	SIGMA-ALDRICH	Oligo#8810932277-000040
TKT_2	SIGMA-ALDRICH	Oligo#8810932277-000050
Software and algorithms		
Enhanced Volcanoplot software (Figure 4)	Bioconductor	https://bioconductor.org/packages/release/bioc/html/EnhancedVolcano.html
FlowJo 10.7.1	FlowJo	https://www.flowjo.com/solutions/flowjo
GeneAnalytics	LifeMap Sciences	https://geneanalytics.genecards.org/
GraphPad Prism6	GraphPad Software	https://www.graphpad.com/
Image Studio Lite	LI-COR Biotechnology	https://www.licor.com
ImageJ	U.S. Department of Health & Human Services	https://imagej.nih.gov/ij/download.html
INKA (original and own mouse modification)	Molecular System Biology; this paper	Beekhof et al.[22]
MACSQuantify	Miltenyi Biotec	https://www.miltenyibiotec.com
MaxQuant	Max-Planck-Institute of Biochemistry	https://maxquant.net/maxquant/
NDP.view2 Plus Image viewing software U12388-02	Hamamatsu Photonics Deutschland GmbH	https://www.hamamatsu.com/eu/en/product/life-science-and-medical-systems/digital-slide-scanner/U12388-01.html
Perseus	Max-Planck-Institute of Biochemistry	https://maxquant.net/perseus/
PRIDE database	EMBL-EBI	https://www.ebi.ac.uk/pride/
StringAnalysis	STRING Consortium 2021	https://string-db.org

EXPERIMENTAL MODEL AND STUDY PARTICIPATION DETAILS

Mouse strains

Wild type NOD.Cg-Prkdcscid Il2rgtm1Wjl/SzJ (NSG) and C57BL/6 mice were from Jackson laboratory (ME, USA), Acod1eml(IMPC) J/1J mice were kindly provided by Paul Diefenhardt from AG Braehler (Uniklinik Köln, CECAD Research Center, AG Braehler, Joseph-Stelzmann-Str. 26, 50931 Cologne, Germany). NSG is an immune-deficient strain with compromised generation of lymphocytes, natural killer cells, macrophages, and immunoglobulins caused by lacking expression of PRKDC and the γ-chain of interleukin-2 receptor. Acod1eml(IMPC)J/1J is a mouse strain with global guide RNA mediated knockout of Acod1 (=Irg1) on background of C57BL/6 mice. C57BL/6 is a commonly used immune competent inbreeding strain. To generate the humanized double-hit lymphoma mouse model, 8–18 week old NSG mice got intravenous injection of 1×10^6 hMB cells in 100μL PBS.

Animals (female and male; 0–40 weeks) were maintained under specific pathogen free conditions in line with European Union regulations. Experiments were approved by local ethical review (LANUV (Landesamt für Natur, Umwelt und Verbraucherschutz Nordrhein-Westfalen)) and were carried out under the authority of Michael Michalik M. Sc., (Uniklinik Köln, Translational Research for Infectious Diseases and Oncology (TRIO), Robert-Koch-Straße 21, 50931 Cologne, Germany) project license.

Mice were kept under cage conditions at 20°C–22°C. Littermates of the same sex were randomly assigned to experimental groups. Adult mouse weight is around 23,4 g.

Cell lines

HEK293T-CAF40-null cells were from DSMZ, hMB cells were generated by Leskov et al.,[36] J774A.1 macrophages were from ATCC, THP1 monocytes were from DSMZ.

hMB cells represent a humanized mouse model of “double-hit” lymphoma by overexpression of c-MYC and BCL2 in human HSC-derived B-lineage cells. hMB cells express GFP and can be targeted by antibodies used in clinics. Like most double-hit lymphoma patient cells, hMB cells have a low expression of CD20.

All cell lines were cultured on 6 well plates or 10cm dishes from Corning and incubated with 5% CO_2 at 37,0°C. J774A.1 cells, hMB cells, and HEK293T cells were cultured in DMEM containing 10% fetal bovine serum and 1% penicillin/streptomycin. THP1 cells were cultured in RPMI 1640 medium containing 10% FBS and 1% penicillin/streptomycin.

Primary cells

Primary CLL patient cells and primary monocytes from healthy donors were collected from buffy coats donated by blood bank of University Hospital of Cologne. Cells were cultured on 6 well plates or 10cm dishes from Corning and incubated with 5% CO_2 at 37,0°C. The cells were cultured in RPMI 1640 medium containing 10% FBS and 1% penicillin/streptomycin.

Monocytes of buffy coats were separated by CD14 anti-human magnetically labeled MicroBeads from Miltenyi Biotec.

The study was approved by the ethical commission of the medical faculty of the University of Cologne (reference no. 13–091) and performed under the authority of Prof. Michael Hallek (Department I of Internal Medicine, Center for Integrated Oncology (CIO) Aachen-Bonn-Cologne-Duesseldorf, University of Cologne, Kerpener Str. 62, 50937 Cologne, Germany) project license.

Microbe strains

5-alpha competent E. coli from New England Biolabs Inc. were used for plasmid generation.

METHOD DETAILS

Antibody-dependent cellular phagocytosis assay (ADCP)

1×10^4 J774A.1 cells were plated out in 100µL media per well in a 96 well plate. After 24hrs of attachment, hMB cells were added in a macrophage:hMB ratio 1:15. Compounds were added in increasing concentration up to maximal non-toxic concentration. Alemtuzumab was added to every second well in a concentration of 10µg/mL. Wells were filled up with macrophage medium up to volume of 250µL. Each ADCP was performed with 5 technical replicates. After 18hrs of incubation, remaining hMB cells per well (GFP positive) were measured using Miltenyi MacsQuant VYB flow cytometer. Out of the absolute GFP positive cell count the antibody-dependent cellular phagocytosis rate was calculated with the formula

100-(100*(total GFP^+ antibody-treated well/total GFP^+ antibody-untreated well)).

The calculated ADCP rate was compared to basal ADCP rate of untreated control cells (set as 100%) by division of the calculated ADCP rates (= ADCP change). For ADCP assays performed with THP1 cells the amounts were subtracted to avoid bias as basal phagocytosis rate of THP1 cells is low (= ADCP difference).

For pre-treatment assays, macrophages respectively hMB cells were treated with increasing concentration of the compounds up to maximal non-toxic concentration. After 24hrs of incubation, the cells were washed three times and the assay was performed as described above without addition of the compounds to co-culture.

Performing the assay with THP1 cells, the antibody obinutuzumab was used in a concentration of 1 µg/mL.

Compounds and antibodies were diluted in media of used macrophage type respectively in hMB medium in case of hMB pre-treatment ADCP assays.

For ADCPs in hypoxia, cells were incubated under hypoxic conditions with 1.5% O_2 and 5% CO_2.

For ADCPs with CLL patient cells, CLL patient cells were used instead of hMB cells.

For ADCPs with primary human macrophages, 2×10^4 primary human macrophages and 3×10^5 hMB cells were used per well. As antibody daratumumab in a concentration of 10µg/mL was used.

For ADCPs with primary murine macrophages, 5×10^4 primary murine macrophages and $1{,}5 \times 10^5$ hMB cells were used per well. As antibody daratumumab in a concentration of 10µg/mL was used.

Antibody-independent cellular phagocytosis assay (AICP)

Experiment was performed like ADCP without the addition of antibody. Remaining hMB cells were compared to hMB mono-culture cell count under inhibitor treatment. After 18hrs of incubation, remaining hMB cells per well were measured using Miltenyi MacsQuant VYB flow cytometer. Out of the absolute GFP positive cell count the antibody-independent cellular phagocytosis rate was calculated with the formula

100-(100(total GFP^+ macrophage co-culture well/total GFP^+ hMB mono-culture well))*.

Bone marrow derived macrophage generation

Femur were flushed with DMEM media. Erythrocytes were lysed by addition of 2mL ACK lysis buffer to cells. Reaction was stopped by addition of 50mL cold PBS. Cells were re-suspended in media and plated out on 10cm cell culture plates for 24hrs. Non adherent cells were collected and plated out in a concentration of 6×10^5 cells/mL on 10cm cell culture plates in media and 15% feeder media 1 and 2. Feeder media was collected from L-929 cells after incubation with RPMI media for one week (feeder media 1) and three weeks (feeder media 2). On day three additional 4mL media with 15% feeder media 1 and 2 and 50ng murine recombinant M-CSF were added. On day seven media was replaced by 10mL media. On day eight the plates were washed two times and adherent macrophages were detached by scraping. Macrophages were plated out for further experiments and were incubated for 24hrs for recovering before further experiments were performed.

CLL patient cell co-culture

5×10^4 J774A.1 cells were plated out in 1mL media on a 24 well plate and were incubated for 5hrs. Three samples per condition were treated with the PPP inhibitors for macrophages pre-treatment. Cells were incubated for 24hrs. Pre-treated cells were washed three times. Viability of CLL patient cells was measured by 7AAD plus AnnexinV staining. Therefor, 2μL 7AAD stain, 2μL AnnexinV stain and 46μL 1% ABB were added to washed cells, cells were incubated for 20 min at 4°C and additionally 50μL 1% ABB was added. Readout was performed immediately using Miltenyi MacsQuant X flow cytometer. $7{,}5 \times 10^5$ viable CLL patient cells in 1mL media were added to the macrophages and CLL mono-culture wells were plated out under addition of 1mL DMEM. Three samples per condition were treated with PPP inhibitors for co-culture treatment. The co-culture was incubated for three days. CLL cells were re-suspended by pipetting and supernatant was transferred into eppies. On U-bottom plates 7AAD plus AnnexinV staining (see above) was performed and cell count was measured using Miltenyi MacsQuant X flow cytometer.

CLL patient cell chemotoxicity stain

CLL patient cells after CLL co-culture performance were transferred into 96 U-bottom plates. 25μL of each bendamustine concentration was added to one well of each condition. Cells were incubated for 48hrs. Cells were washed and 7AAD plus AnnexinV staining was performed (see above).

Chemotoxicity assay was also performed by adding the cells to 1×10^4 J774A.1 cells per well plated out the day before. After co-incubation for 48hrs, wells were mixed up and supernatant was transferred to a 96 U-bottom plate to perform 7AAD plus AnnexinV staining of the CLL patient cells (see above).

ELISA

7×10^5 J774A.1 cells in 2mL media per well were plated out on a 12 well plate and incubated for 24hrs. Each inhibitor was added to two wells and cells were incubated for 24hrs. 100ng/mL LPS was added to one well of each condition. After incubation of 16hrs, supernatant was transferred into Eppendorf tubes. The supernatant was centrifuged at 300g for 5min and transferred to new Eppendorf tubes. BioLegend ELISA kit for IL-10, and IL-6 was used and protocol performed as from BioLegend mentioned. Readout was performed by fluorescence intensity measurement using FLUOStar OPTIMA.

Immunofluorescent microscopy

Microscopy coverslips were washed three times in ethanol and autoclaved. Two microscopy coverslips per well were placed on a 6 well plate. 1×10^6 J774A.1 cells in 1mL media per well were plated out, treated with the PPP inhibitors and incubated for 24hrs. Cells were washed with 5mL DPBS for 5min. 2mL PFA was put on the cells and incubated for 10min. Cells were washed three times with 5mL DPBS for 5min. Cells were incubated with 2mL of DPBS with 0.25% Triton X- for 3min. Cells were incubated with 2mL of DPBS with 0.125% Triton X- plus 5% BSA for 1h. Coverslips were taken off the 12 well plate and dried on a paper towel. Mitochondrial antibody TOM20 F-10 was diluted 1:500 in DPBS plus 0.125% Triton X- plus 2.5% BSA, 100μL was pipetted on the coverslips and they were incubated for 2hrs. Coverslips were washed three times with 100μL DPBS with 0.125% Triton X-. 2nd mitochondrial antibody (Alexa Fluor 647 α mouse) was diluted 1:1000 in DPBS plus 0.125% Triton X- plus 2.5% BSA, 100μL was pipetted on the coverslips and they were incubated for 1h. Coverslips were washed three times with 100μL DPBS with 0.125% Triton X-. Actin antibody (Phalloidin Alexa Fluor 568) was diluted 1:1000 in DPBS with 0.125% Triton X- plus 2.5% BSA, 100μL was pipetted on the coverslips and they were incubated for 1h. Coverslips were washed nine times with 100μL DPBS with 0.125% Triton X-. Nuclear antibody (DAPI) was diluted 1:10000 in DPBS and 100μL was pipetted on the coverslips. Coverslips were washed one time with 100μL H_2O. Coverslips were dried on a paper towel, one drop of mounting media was put on and the coverslips were placed on object plates. The probes were dried overnight at room temperature and then stored at 4°C until microscopy. Pictures were recorded using SP8 confocal microscope (Leica) and analyzed with ImageJ.

Immune phenotyping

1×10^6 J774A.1 cells in 1mL media were plated out on a 6 well plate and treated with PPP inhibitors. After 24hrs of incubation, cells were scraped off. Cells were washed with 1mL DPBS. Cells were re-suspended in 90μL DPBS and 10μL murine FcR-blocking agent. Cells were incubated for 10min at 4°C. 900μL DPBS were added and probes were split into 100μL portions. Master mixes of different stains were prepared and added to the cells. Cells were incubated for 20min at 4°C and washed with 1mL DPBS afterward. For washing, cells were incubated with DPBS for 5min and centrifuged at 300g for 5min afterward. Cells were re-suspended in 200μL DPBS and measured using Miltenyi MacsQuant X flow cytometer immediately after an initial multi-color compensation procedure.

For intracellular marker, Fix & Perm Cell Permeabilization kit was used following manufacturés protocol.

Primary murine macrophages were processed equally.

Immunohistochemical staining of murine spleen

After preparation of survival cohort of hMB transfected NSG mice after treatment with alemtuzumab and/or S3, a 2 × 2mm piece of spleen was fixated in formaldehyde. Histological sections were produced and immunohistochemical staining of CD19 and CD68 was performed. Whole slide scans were saved and analyzed with NDP.view2 Plus Image viewing software.

In vivo experiments

Survival analysis of hMB transfected NSG mice under treatment with alemtuzumab and/or S3

8-18 week old NSG mice got intravenous injection of 1 × 10^6 hMB cells in 100μL PBS.

Four cohorts were built:

(1) vehicle + vehicle
(2) vehicle + alemtuzumab
(3) vehicle + S3
(4) alemtuzumab + S3

Three days after tumor cell injection, cohort 3 and 4 were treated for ten days with 20mg/kg S3 in 200μL 30% PEG 400/0.5% Tween 80/5% propylene glycol (vehicle 200μL 30% PEG 400/0.5% Tween 80/5% propylene glycol) intraperitoneally. On day 8 after tumor cell injection alemtuzumab was applicated for three days intraperitoneally. On day 8 the mice were injected with alemtuzumab 1mg/kg, on day 9 and 10 with 5mg/kg, in a total volume of 50μL PBS (vehicle 50μL PBS). Mice were scored daily with a score sheet developed for hMB-transfected NSG mice.

Macrophage function analysis of primary murine macrophages of C57BL/6 after treatment with S3

8-18 week old C75BL/6 mice were treated for seven days with 20mg/kg S3 in 200μL 30% PEG 400/0.5% Tween 80/5% propylene glycol (vehicle 200μL 30% PEG 400/0.5% Tween 80/5% propylene glycol) intraperitoneally. Mice were scored daily and were sacrificed on day eight. Peritoneal macrophages were collected by peritoneal lavage with DMEM medium. Spleen and femurs were dissected. Spleen were mashed through a 30μM filter with DMEM medium and cell suspension was used for further analysis. Femurs got flushed with DMEM medium to collect bone marrow.

Knockdown cell production

Plasmid production

Target sequence oligonucleotides were produced by SIGMA-ALDRICH. For oligonucleotide cloning, 1μL of 1μM oligonucleotide, 2,5μL thermopol polymerase buffer, 2,5μL of 5μM primer EcoR1, 2,5μL of 5μM primer Xho1, 0,5μL dNTPs mix, 0,5μL vent polymerase and 14,5μL H_2O were mixed and polymerase chain reaction was performed. Oligonucleotides were selected by electrophoretic separation using FAE gel with 1.5% agarose and 5μL GELRed Nucleic Acid Gel stain 10000x. After cutting out oligonucleotide bands under UV-light, oligonucleotides were purified with QIAquick PCR purification kit (kit protocol followed). For oligonucleotide digest, 30μL oligonucleotide solution, 1μL primer EcoR1, 1μL primer Xho1 and 8μL NEBuffer 2 were incubated for 2hrs at 37°C. Oligonucleotides were purified again as described above, oligonucleotide concentration was determined with Quick-load 100bp DNA ladder on gel and oligonucleotides were solved in 30μL elution buffer. For ligation 2.92ng oligonucleotide and 97,08ng vector [MLP plasmid, 7893bp] with 1μL 10x T4 DNA Ligase Reaction Buffer and 1μL T4 DNA ligase were used and filled up with H_2O to 10μL. Probes were incubated for 1h at room temperature.

Transformation into competent bacteria

Competent E. coli were thawed on ice. 5μL plasmid per 50μL competent cells was added and incubated for 30min on ice. Heat shock for 110s at 42°C and recovery for 2min on ice was done. Cells were centrifuged at 6000rpm for 1min, supernatant was removed and 450μL fresh LB media (1% tryptone, 0.5% yeast extract, 1% NaCl, filled up with water) was added. Cells were recovered in shaker for 30min at 37°C and 100μL were plated out on LB media layer (1% tryptone, 0.5% yeast extract, 1% NaCl, 1.5% agar filled up with water) with 100μg/mL ampicillin in a 10cm dish.

Plasmid enrichment and purification

When colonies on bacteria plate were visible by eye, 4–5 colonies per plasmid were picked and expanded overnight in 50mL LB media with 100μg/mL ampicillin in shaker at 37°C. For later use, a probe of each colony was saved on a new bacteria plate. Plasmids were purified by using I-Blue Midi Plasmid Kit (following kit protocol).

For sequence verification, 10μL of 100ng plasmid with 4μL MSV-5 primer was sent to LGC Genomics GmbH.

Plasmids with correct sequence were picked from back up bacteria plate and expanded and purified again as described above.

Transfection with phoenix retroviral producer line

8 × 10^6 HEK293T cells were plated out on a 10cm dish and incubated overnight. Cells were washed and 10mL DMEM media without any supplements was put on the cells. 25μM cholorquine was added and cells were incubated for 10min at 37°C. 18,5μg plasmid, 3μg pMD2.G, 5μg psPax2 and 99μL 2M $CaCl_2$ were mixed and filled up with H_2O to 790μL. Under mixing with bubble formation, 790μL 2x HEPES buffered saline (280mM NaCl, 50mM HEPES, 1.5mM Na_2HPO_4, adjusted pH to 7,05) was added and mixture was added immediately dropwise to the HEK293T cells. Cells were incubated for 4hrs, then media was soaked off, cells were washed with 10mL DPBS and 10mL HEK293T cell media was added. 100μM sodium butyrate was added and cells were incubated. After 24hrs and 48hrs supernatant was collected and centrifuged at 1500rpm for 10min. Supernatant was filtered through a 45μm filter and flow through was used for further steps. New media and sodium butyrate was put on the HEK293T cells.

Virus production was verified with fluorescent microscopy and Lenti-X GoStix Plus.

Infection of J774A.1 macrophages

1 × 10^5 J774A.1 cells in 1mL media per well were plated out on a 12 well plate and incubated for 24hrs. 1mL of viral media per well was added and cells were spin at 800CF for 2hrs at 32°C. Cells were incubated for 48hrs. Cell media was changed and cell selection with 2μg/mL puromycin was started. Selection efficacy was verified using Miltenyi MacsQuant X flow cytometer. ≥ 95% GFP-positive macrophages were accepted as pure shRNA-transfected cells.

Metabolomics

Sample preparation

1 × 10^7 J774A.1 (wild-type, empty vector control, shRNA 6PGD knockdown or shRNA TKT knockdown) in 10mL media were plated out on 10cm dishes and incubated for 24hrs. Dishes were treated with PPP inhibitors (oxythiamine 520μM, physcion 9μM or 6-aminonicotinamide 11μM) and incubated for 24hrs. Cells were scraped, washed with DPBS and re-suspended in 1mL DPBS. The cells were counted using CASY cell counter and analyzer. Cells were centrifuged at 300g for 5min and supernatant was discarded. Cell pellet was crash frozen using liquid nitrogen.

The cell pellets were sent to the Center of Metabolomics and Bioanalysis (CEMBIO) at San Pablo CEU University in Madrid (Spain). The analysis of metabolites was performed in the laboratory of Dr. Coral Barbas following previous work.[31]

Macrophage pellet was resuspended in 50μL of cold MeOH, vortex-mixed for 2min and incubated on ice for 5min in order to precipitate the protein content. Samples were sonicated for 4min to break the cell membranes, and 50μL of water was added to extract and solubilize metabolites. Samples were vortex-mixed for 2min and centrifuged for 20min at 4°C at 12.000g. 60μL of the supernatant was transferred to a liquid chromatography (LC) vial for the analysis. 20μL of the remaining supernatant of each sample were taken to make a pool solution (named quality control, QC). The QC sample was used to estimate the concentration of the metabolites and these concentrations were selected as the intermediate point of external calibration curve (100%). The selected range for the external calibration curve was from 25 to 800%. The external calibration curve was prepared using equal volumes 1:1 (*v/v*) of the QC sample and level each of the standard levels leading to the final levels of 25%, 50%, 100%, 200%, 400%, and 800% (calibration curve levels L1-L6, respectively).

LC MS/MS analysis

Sample analysis was performed in an Agilent 1290 Infinity high-pressure liquid chromatography system (HPLC), consisting of a degasser and an autosampler, and using an Infinity Binary Pump (1200bar) and a 400bar 1260 Infinity Quaternary Pump (both from Agilent Technologies, Waldbronn, Germany). The HPLC system was coupled to an Agilent 6460 triple quadrupole mass spectrometer using an electrospray (ESI) source working in dynamic multiple reaction monitoring (dMRM) mode (Agilent Technologies, 465 Waldbronn, Germany). 6μL of sample supernatant was injected into a reverse-phase column (Zorbax Extend-C18 (1.8μm, 2.1 mm × 15 cm, Agilent Technologies, CA, USA) with a guard column (Zorbax Extend-C18 guard (1.8μm, 2.1 mm × 5 mm, Agilent Technologies, CA, USA), maintained at 50°C. LC-QqQ/MS mobile phases consisted of mobile phase A prepared by mixing 97% Milli-Q water (v/v) with 3% (v/v) MeOH, 10mM tributylamine (≥99.5%), and 15mM glacial acetic acid and mobile phase B, prepared by adding 10mM tributylamine and 15mM glacial acetic acid to MeOH. The LC-QqQ/MS mobile phase for the quaternary bump consisted of mobile phase C, identical to mobile phase A, and mobile phase D made of ACN. The chromatographic separation was based on gradient elution using a binary pump at a flow rate of 0.25mL/min with a composition of 0% B from time 0−2.5min, and then % B was progressively increased until 20% B at 7.5min, up to 45% B at 13.0min, and up to 99% B at 20.0min, which was held until 24.0min. Then, the equilibration step started and the flow of the binary pump was stopped at 24.05min while allowing a subsequent quaternary pump washing step. It started with 99% C at 24.0min with a flow rate of 0.2mL/min until 27.0min, and then the flow rate was gradually increased until 0.3mL/min at 27.5min and until 43.5min. Subsequently, % C was progressively decreased up to 0% C while returning the flow rate to 0.2mL/min at 52.25min. These conditions were held until 59.0min and returned to 99% C at 59.9min. Finally, the binary pump was activated at 0% B at 59.0min, with a flow rate of 0.2mL/min, and increased up to 0.25mL/min at 60.0min. The total method run time was 60min.

Metabolites were ionized in an ESI source operating in negative ionization mode. The drying gas flow rate was 13L/min at 225°C, and the nebulizer was set to 60psi. The sheath gas flow rate was set to 12L/min at 250°C; capillary and nozzle voltages were set to 3500V and 2000V, respectively. Data were acquired in dynamic MRM mode, using a cycle time of 1000ms. Transitions showing the highest signal-to-noise ratios were used for the quantification of the metabolites in samples. For those metabolites with two transitions with a good signal-to-noise ratio, the less intense transitions were used for identification and confirmation of the metabolite (see Table S6).

Sample quantitation and statistical analysis

Samples were analyzed in a randomized order, injecting the calibration curve from the lower to the higher concentrated ones in regular intervals. Output raw data files were reprocessed with Agilent MassHunter Workstation Software Quantitative Analysis for QQQ, from which a metabolite matrix containing the integrated area and retention time (RT) for specific transitions was obtained. The concentration of the metabolites in the samples were calculated by interpolation in calibration curves and normalized by the number of cells. Statistical analysis and graph representation was carried out using GraphPad Prism (v.9.5.0) software.

Maturation staining of primary murine macrophages

For LSK compartment analysis, ACK lysis buffer was added to bone marrow cells for 2min to lysate erythrocytes. Bone marrow cells were processed as described in section Immune phenotyping and stained with maturation antibody panel. Common myeloid progenitor cells: CD41, CD34. Monocytes: CD11b, CX3CR1, Ly6C. Macrophages: CD11b, F4/80, CD64. LSK compartment analysis was performed by Felix Picard (AG Holger Winkels, University of Cologne) with 5×10^6 cells per mice.

Phosphoproteomics

Sample preparation and lysis

Three dense 25cm dishes with J774A.1 cells were treated with PPP inhibitors. After 24hrs incubation, plates were washed with 20mL 4°C DPBS per plate. Cell dishes were placed on ice, 1mL RIPA buffer with 1% phosphatase and 1% protease inhibitor was added, cells were scraped off and transferred into 2mL Eppendorf tubes on ice. Cells were centrifuged at full speed for 30min at 4°C. DNA was sheared by sonication with Bioruptor for 10min at 4°C. Samples were centrifuged at full speed for 10min at 4°C and supernatant was transferred into new Eppendorf tubes. Protein concentration was determined by BCA assay. 3mg protein was used for further steps.

Acetone precipitation

Four times volume of cold 100% acetone was added and samples were incubated overnight at −20°C. Samples were centrifuged at 15000g for 10min at 4°C. Supernatant was discarded and pellet washed twice with 250μL 80–90% acetone under centrifugation at 15000*g* for 10min at 4°C. Uncapped tubes were left at room temperature for 5-10min to let remaining acetone evaporate without overdrying. Pellet was dissolved in 300μL 6M Urea/2M Thiourea.

In solution digest

1mM 1,4-Dithiothreit (DTT) was added and samples were incubated for 1h at room temperature. 5.5mM iodoacetic acid (IAA) was added and samples were incubated for 20-45min at room temperature in the dark. 60μL 0.5μg/μL endoprotease Lys-C was added and samples were incubated for 3hrs at room temperature. 900μL 50mM ammonium bicarbonate and 60μL 0.5μg/μL trypsin were added and samples were incubated overnight at room temperature. Samples were acidified with 1% trifluoroacetic acid and centrifuged for 10min at full speed. Supernatant was transferred to a new Eppendorf tube for further steps.

Sample purification by stage tips

C18 columns (200mg Sep Pak of capacity up to 10mg protein) were prepared. Columns were activated with 1mL 100% acetonitrile and washed twice with 1mL 0.1% trifluoroacetic acid. Flow through was discarded and samples were loaded on column. Flow through was discarded and column was washed twice with 1mL 0.1% trifluoroacetic acid. Peptides were eluted into Eppendorf tubes by adding two times 0.2mL elution buffer (60% acetonitrile, 0.1% formic acid).

Sample enrichment

Phosphopeptide samples were enriched using High select TiO_2 Phosphopeptide Enrichment Kit by following kit protocol.

Sample measurement

Label-free quantification of peptides was performed on mass spectrometer (Q Exactive Plus Hybrid Quadrupole-Orbitatrap Mass Spectrometer + EASY-nLC 1200 System) by cooperating CECAD proteomics facility, University of Cologne.

Analysis

Raw data acquired from CECAD proteomics facility were filtered and processed on MaxQuant software (v.1.5.3.8) and Perseus software (v.1.5.5.3).

Primary human macrophages

Isolation of $CD14^+$ cells

Peripheral blood mononuclear cells (PBMCs) were isolated from buffy coats of healthy donors provided by blood bank of University Hospital of Cologne. 15mL Lymphoprep was added to a 50mL SepMateTM tube, buffy coats were diluted 1:1 in sterile room temperature DPBS and 20-25mL of it was layered on the top of the Lymphoprep through pipetting it to the walls of the SepMateTM tube. Tubes were centrifuged at 1200g for 15min at room temperature. PBMCs ring was harvested by pouring entire top layer in new 50mL falcon tube. Cells were washed three times with 50mL DPBS and centrifuged at 1300rpm for 8min. Cell pellet was re-suspended in 12mL 4°C MACS buffer (2mM EDTA +5% Bovine serum albumin (BSA) (PAA Laboratories)), transferred to 15mL tube, centrifuged at 1300rpm for 8min at 4°C and discarded. 200μL of CD14 anti-human magnetically labeled MicroBeads and 800μL of 4°C MACS buffer was added. Magnetic separation was performed by Miltenyi Biotec CD14 human microbead isolation protocol using Miltenyi MacsQuant X flow cytometer. $CD14^+$ cells were then re-suspended in RPMI 1640 media. Differentiation was started immediately.

Differentiation of human monocytes under PPP inhibition

1×10^6 $CD14^+$ cells in 2mL RPMI 1640 media per well were plated out on a 12 well plate. 10ng/mL M-CSF and oxythiamine were added at day 1, 3 and 5 without changing the medium. On day 7 the cells were scraped off and used for further experiments.

Proteomics

Sample preparation and lysis

1×10^7 J774A.1 cells ins 10mL media per 10cm dish were plated out and after 24hrs treated with PPP inhibitors. After another 24hrs, plates were washed with 10mL 4°C DPBS per plate. Cell dishes were placed on ice, 500μL RIPA buffer with 1% phosphatase and 1%

protease inhibitor was added, cells were scraped off and transferred into 2mL Eppendorf tubes on ice. Cells were centrifuged at full speed for 30min at 4°C. DNA was sheared by sonication with Bioruptor for 10min at 4°C. Samples were centrifuged at full speed for 10min at 4°C and supernatant was transferred into new Eppendorf tubes. Protein concentration was determined by BCA assay. 30μg protein was used for further steps.

Acetone precipitation

Four times volume of cold 100% acetone was added and samples were incubated overnight at −20°C. Samples were centrifuged at 15000g for 10min at 4°C. Supernatant was discarded and pellet washed twice with 250μL 80–90% acetone under centrifugation at 15000*g* for 10min at 4°C. Uncapped tubes were left at room temperature for 5-10min to let remaining acetone evaporate without overdrying. Pellet was dissolved in 60μL 6M urea/2M thiourea.

In solution digest

3μL 1M DTT was added and samples were incubated for 1h at room temperature. 3μL 550mM IAA was added and samples were incubated for 20-45min at room temperature in the dark. 0,6μL 0.5μg/μL endoprotease Lys-C was added and samples were incubated for 3hrs at room temperature. 180μL 50mM ammonium bicarbonate and 0,6μL 0.5μg/μL trypsin were added and samples were incubated overnight at room temperature. Samples were acidified with 1% trifluoroacetic acid and centrifuged for 10min at full speed. Supernatant was transferred to a new Eppendorf tube for further steps.

Sample purification by stage tips

Stage tips were prepared by stacking 2 layers of SDB-RPS material in a 200μL pipette tip. Stage tips were equilibrated with 20μL 100% methanol and centrifuged at 2600rpm for 2min. 20μL elution buffer (80% acetonitrile +0.1% trifluoroacetic acid) was added and tips were centrifuged at 2600rpm for 2min. 20μL washing buffer (0.1% trifluoroacetic acid) was added and tips were centrifuged at 2600rpm for 1min. 100μL of the sample was added and tips were centrifuged at 2600 rpm for 5min. Tips were washed with 100μL washing buffer and centrifuged at 2600rpm for 3min once and two times with elution buffer. Stage tips were dried with a syringe and stored at −4°C.

Sample measurement

Peptides were eluted with 30μL 1% ammonia in 60% acetonitrile into 96 well plate and dried using SpeedVac concentrator. Label-free quantification of peptides was performed on a mass spectrometer (Q Exactive Plus Hybrid Quadrupole-Orbitatrap Mass Spectrometer + EASY-nLC 1200 System) by cooperating CECAD proteomics facility, University of Cologne.

Analysis

Raw data acquired from CECAD proteomics facility were filtered and processed on MaxQuant software (v.1.5.3.8) and Perseus software (v.1.5.5.3).

SeaHorse analysis

1 × 10^5 J774A.1 cells in 100μL media per well were plated out in the XFe96 cell culture microplate. PPP inhibitors were added and cells were incubated for 24hrs. Agilent Seahorse XFe96 Sensor Cartridge was prepared (following Agilent user guide) and cell culture microplate and sensor cartridge preparation for measurement were done (following Agilent protocol). Sensor cartridge injection ports were filled with 20μL of 1μM oligomycin (port A), 22μL of 0.5μM Carbonyl cyanide-*4*-(trifluoromethoxy)phenylhydrazone (FCCP) (port B), and 25μL of 1μM antimycin A plus 100μM rotenone (port C). Four measurement cycles of basal activity of the cells and three cycles of measurement after each injection were performed by Agilent Seahorse XF Analyzer.

Viability stain

7AAD staining was performed for hMB cells and Zombie-NIR staining was performed for J774A.1 cells.

For 7AAD staining, 1,5 × 10^5 hMB cells were plated out in 100μL media per well on a 96 well plate. At least duplicates were treated with one concentration of the tested inhibitor. As positive control 10% dimethylsulphoxide (DMSO) was used. After 24hrs of incubation, cells were transferred into a 96 well U-bottom plate, washed and 7AAD staining was performed. Therefore, 2μL 7AAD stain and 48μL 1x ABB were added per well, cells were incubated for 20min at 4°C and additionally 50μL 1% ABB was added. Readout was performed immediately using Miltenyi MacsQuant X flow cytometer. Inhibitor concentrations with an amount of viable cells ≥90% compared to untreated control were accepted as non-toxic concentration.

For Zombie-NIR staining, 1,2 × 10^6 J774A.1 cells were plated out in 1mL media on a 12 well plate. Duplicates were treated with one concentration of the tested inhibitor. As positive control 10% DMSO was used. After 24hrs of incubation, cells were scraped off, transferred into FACS tubes and Zombie-NIR staining was performed. A dilution of Zombie-NIR staining solution 1:100 in DPBS was used. Readout was performed using Miltenyi MacsQuant X flow cytometer. Inhibitor concentrations with an amount of viable cells ≥90% compared to untreated control were accepted as non-toxic concentration.

Additionally, Cell titer glo assay was used to measure viability of J774A.1, THP1 and hMB cells. 1 × 10^4 J774A.1 cells or THP1 cells respectively 1,5 × 10^5 hMB cells were plated out in 100μL media per well on a 96 well plate. Triplets were treated with one concentration of the tested inhibitor. As positive control 10% DMSO was used. After 18hrs, cells were washed two times, were transferred to white 96 well plate and Cell titer glo staining was performed. Readout was performed by fluorescence intensity measurement with FLUOStar OPTIMA. Inhibitor concentrations with an ATP amount of the cells ≥90% compared to untreated control were accepted as non-toxic concentration.

Western Blot analysis

3 × 10^6 J774A.1 respectively 4,5 × 10^6 hMB cells were plated out on a six well plate. The cells were treated and incubated for 18hrs. The cells were washed with 1mL DPBS, discarded and stored on ice. 30µL of RIPA buffer (50mM Tris-HCl pH8, 150mM NaCl, 0.1% SDS, 0.5% DOC, 1% NP-40, filled up with ddH2O) with 1x Phosphatase Inhibitor Cocktail 2 and 1x Protease Inhibitor Cocktail were added and probes centrifuged for 30min at full speed at 4°C. Supernatant was used for the experiments. BCA-Assay was performed to evaluate protein concentration, measured with FLUOStar OPTIMA. 60µg protein per condition was used, volume filled up with RIPA to 5µL and 5µL Urea added. Probes were incubated at 37°C for 10min. A 10% separating gel (1.85mL Buffer (1.5mM Tris HCL pH8.8, 0.4% SDS, ddH_2O), 1.66mL 30% Rotiphorese, 1.5mL ddH_2O, 40.6µL APS and 4.06µL Temed) and a 5% stacking gel (0.31mL Buffer (0.5M Tris HCl pH6.8, 0.4% SDS, ddH_2O), 0.42mL 30% Rotiphorese, 1.75mL ddH_2O, 12.5µL APS and 1.25µL Temed) were produced. 3.5µL Page Ruler Prestained NIR Protein Ladder was used. Western Blot run was performed in Running Buffer (25mM Tris, 192mM glycine, 3.5mM SDS in ddH_2O) at constant 80V until stacking gel was passed and at constant 150V in separating gel. Gel was blotted on nitrocellulose membrane Hybind-C at constant 400mA for 1h in transfer Buffer (25mM Tris, 192mM glycine, ddH_2O). Total Protein stain was performed with REVERT Total protein stain and membrane was blocked with 10mL TBS-T (10mM Tris, 250mM NaCl, HCl pH7.6, 0.05% Tween 20, ddH_2O) + 5% BSA for 1h. First antibody was diluted in 5mL TBS-T + 5% BSA and membrane was incubated overnight at 4°C in the dark. Membrane was washed to times with 2mL TBS-T for 10min and one time with 2mL TBS (10mM Tris, 250mM NaCl, HCl pH7.6, ddH_2O) for 10min. Second antibody was diluted in 2.5mL TBS + 2.5mL Odyssey Blocking Buffer. Membrane was incubated with second antibody for 1h at room temperature. Membrane was washed three times with TBS for 10min. Membrane fluorescence was measured with ODYSSEY CLx. Fluorescence intensity was calculated with Image Studio Lite Vers. 5.2.

QUANTIFICATION AND STATISTICAL ANALYSIS

Statistical analysis

Statistical analysis was performed using GraphPad Prism software. Significance was calculated using unpaired t-test (Figures 1B–1D and 5F–5H), multiple comparison one-way ANOVA (Figures 2, 3A, 3B, 3E–3G, 4, 5A, 5B, 5D–5E, and 6A–6G), two-way ANOVA (Figure 3D), RM one-way ANOVA (Figure 6H), paired t-test (Figures 6I–6L), student's t-test (Figure 5E), unpaired t-test (Figures 7A–7E), Benjamini-Hochberg test (Figure 7F).

In Figures 1, 2, 4G, 5A, 5B, 5F–5H, 6A–6H, 7A–7C, and 7E data are shown as mean ± SEM. In Figure 3 surface marker stain is shown as mean of four replicates, SeaHorse analysis over time is shown as one representative example mean ± SD, calculated parameters of SeaHorse analysis are shown as mean ± 5–95 percentile. In Figures 5D–5E metabolite amount is shown as minimum to maximum and protein expression is shown as calculated –Log2 fold change of control and knockdown macrophages. In Figures 6I–6L data are shown as minimum to maximum. In Figure 7D surface marker stain is shown as mean of ten replicates.

Statistical values, including technical and biological number of replicates (*n*), are named in the figure legends. *$p < 0.05$; **$p < 0.01$; ***$p < 0.001$; ****$p < 0.0001$.

Proteomic and phosphoproteomic analysis

Raw data acquired from CECAD proteomics facility were filtered and processed on MaxQuant software (v.1.5.3.8) and Perseus software (v.1.5.5.3). Data are generated out of one experiment with three replicates per condition.

Volcano plots of proteomics were generated with software of Bioconductor. The mean of the different inhibitor treatments respectively PPP enzyme knockdowns was calculated and compared to the untreated control. Significance was defined as mean Log_2 fold change >0.5 or < −0.5 and q value <0.05. Circle size represents the number of significant occurrence in the different treatment conditions.

For circle plot analysis, significant genes were extracted from proteomic- and phosphoproteomic analysis. Significance was defined as mean Log_2 fold change >0.5 or < −0.5 and q value <0.05 for proteomic analysis and as mean Log_2 fold change >0.5 or < −0.5 and p value >1.3 for phosphoproteomic analysis. Significant genes were clustered with String analysis. The ten biggest clusters were used for further analysis. Clusters were named by using GeneAnalytics and similar clusters were merged in one heading. Mean of -$\log_{10} p$ value of the clusters was calculated and is represented in heat color, count of genes per cluster is represented in circle size.

Normalized upstream kinase score out of phosphoproteomic analysis was calculated by adapted code of INKA analysis. On basis of the work of Beekhof et al.,[22] we calculated a simplified upstream kinase score analysis, using the murine data available from the PhosphoSitePlus (PSP) database:

$$Upstream\ Kinase\ Score = \sqrt{\sum\nolimits_{Kin} \times \sum\nolimits_{PSP}}$$

With $\sum_{Kin}$ representing the sum of all phosphopeptides observed in the experiment per kinase found in the murine PSP database, whilst $\sum_{PSP}$ representing the sum of all substrate phosphopeptides observed in the experiment associated with each kinase found in the murine PSP database. These scores were calculated for each replicate per condition, with the normalized upstream kinase score (NUKS) representing the mean difference in upstream kinase scores between untreated control and treatment with PPP inhibitors and macrophage wild-type and PPP knockdown macrophages respectively.

Cell Reports Medicine, Volume *5*

Supplemental information

Macrophages are activated toward phagocytic

lymphoma cell clearance

by pentose phosphate pathway inhibition

Anna C. Beielstein, Elena Izquierdo, Stuart Blakemore, Nadine Nickel, Michael Michalik, Samruddhi Chawan, Reinhild Brinker, Hans-Henrik Bartel, Daniela Vorholt, Lukas Albert, Janica L. Nolte, Rebecca Linke, Carolina Raíssa Costa Picossi, Jorge Sáiz, Felix Picard, Alexandra Florin, Jörn Meinel, Reinhard Büttner, Paul Diefenhardt, Sebastian Brähler, Alma Villaseñor, Holger Winkels, Michael Hallek, Marcus Krüger, Coral Barbas, and Christian P. Pallasch

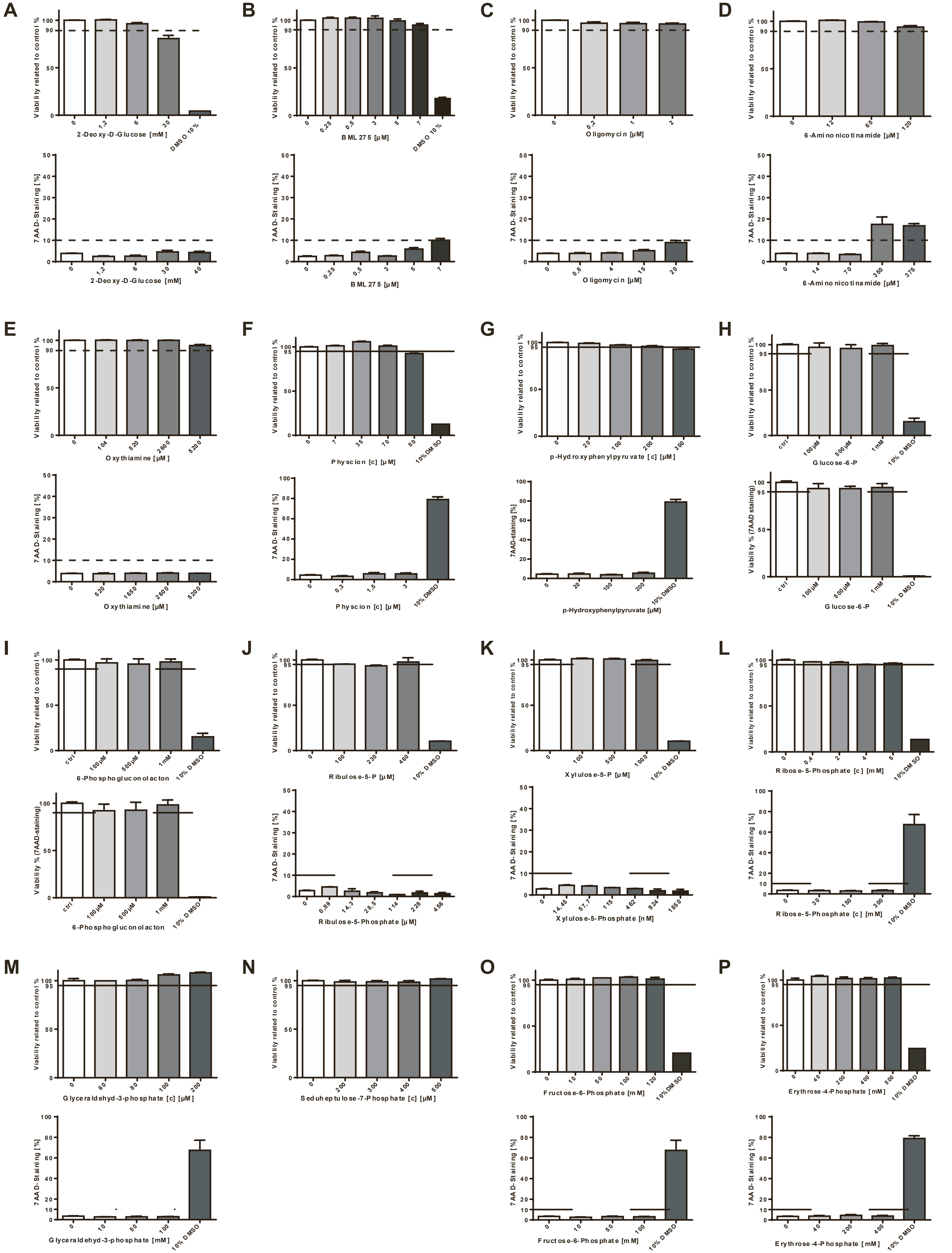
A
B
C
D
Viability related to control %
7AAD-Staining [%]
2-Deoxy-D-Glucose [mM]
DMSO 10%
BML 275 [µM]
Oligomycin [µM]
6-Aminonicotinamide [µM]
E
F
G
H
Oxythiamine [µM]
Physcion [c] [µM]
10% DMSO
p-Hydroxyphenylpyruvate [c] [µM]
p-Hydroxyphenylpyruvate [µM]
7AAD-staining [%]
Glucose-6-P
Viability % (7AAD staining)
ctrl
100µM
500µM
1mM
I
J
K
L
6-Phosphogluconolacton
Viability % (7AAD-staining)
Ribulose-5-P [µM]
Ribulose-5-Phosphate [µM]
Xylulose-5-P [µM]
Xylulose-5-Phosphate [nM]
Ribose-5-Phosphate [c] [mM]
M
N
O
P
Glyceraldehyd-3-phosphate [c] [µM]
Glyceraldehyd-3-phosphate [mM]
Seduheptulose-7-Phosphate [c] [µM]
Fructose-6-Phosphate [mM]
Erythrose-4-Phosphate [mM]

Figure S1. Evaluation of cytotoxicity of used compounds in J774A.1 macrophages and hMB cells. Related to Figures 1-2.

(A-P) Measurement of viable cells under treatment with different inhibitors. Treatment with 10% DMSO used as positive control. Viability of J774A.1 cells (upper plots) was determined by Zombie staining, viability of hMB cells was determined by 7AAD staining. Viability under inhibition was compared to viability of untreated control cells. Used Inhibitors **A** 2-deoxy-D-glucose, **B** BML275, **C** oligomycin, **D** 6-aminonicotinamide, **E** oxythiamine, **F** physcion, **G** p-hydroxyphenylpyruvate, **H** glucose-6-phosphate, **I** 6-phosphogluconolactone, **J** ribulose-6-phosphate, **K** xylulose-5-phosphate, **L** ribose-5-phosphate, **M** glyceraldehyde-3-phosphate, **N** sedoheptulose-7-phosphate, **O** fructose-6-phosphate, **P** erythrose-4-phosphate.

Technical replicates n=4-12; biological replicates n=2-6. Data are shown as mean ± SEM. *P* values were calculated using one-way ANOVA. *$p < 0.05$; **$p < 0.01$; ***$p < 0.001$; ****$p < 0.0001$.

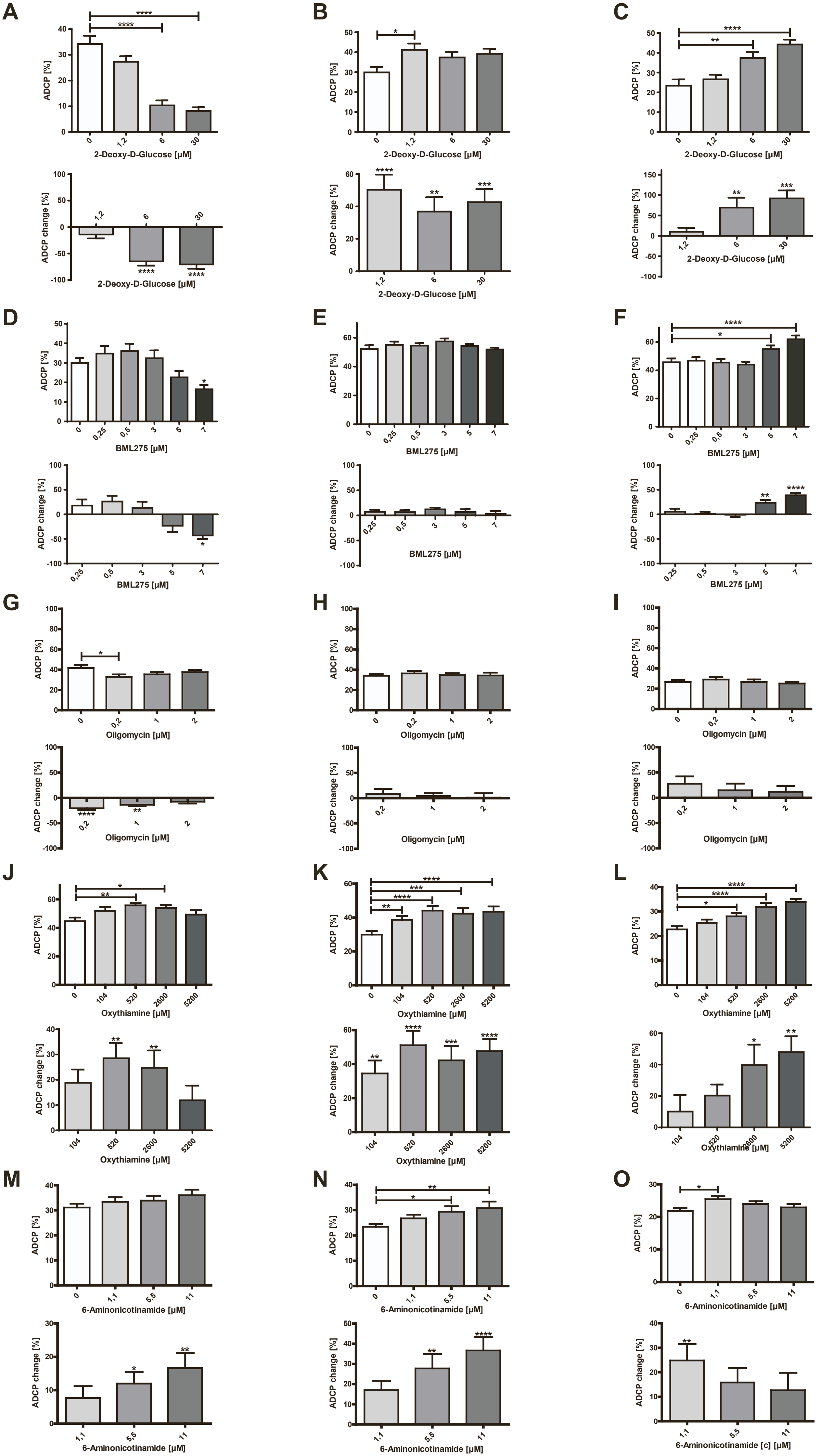
A
ADCP [%]
2-Deoxy-D-Glucose [μM]
ADCP change [%]
2-Deoxy-D-Glucose [μM]
B
ADCP [%]
2-Deoxy-D-Glucose [μM]
ADCP change [%]
2-Deoxy-D-Glucose [μM]
C
ADCP [%]
2-Deoxy-D-Glucose [μM]
ADCP change [%]
2-Deoxy-D-Glucose [μM]
D
ADCP [%]
BML275 [μM]
ADCP change [%]
BML275 [μM]
E
ADCP [%]
BML275 [μM]
ADCP change [%]
BML275 [μM]
F
ADCP [%]
BML275 [μM]
ADCP change [%]
BML275 [μM]
G
ADCP [%]
Oligomycin [μM]
ADCP change [%]
Oligomycin [μM]
H
ADCP [%]
Oligomycin [μM]
ADCP change [%]
Oligomycin [μM]
I
ADCP [%]
Oligomycin [μM]
ADCP change [%]
Oligomycin [μM]
J
ADCP [%]
Oxythiamine [μM]
ADCP change [%]
Oxythiamine [μM]
K
ADCP [%]
Oxythiamine [μM]
ADCP change [%]
Oxythiamine [μM]
L
ADCP [%]
Oxythiamine [μM]
ADCP change [%]
Oxythiamine [μM]
M
ADCP [%]
6-Aminonicotinamide [μM]
ADCP change [%]
6-Aminonicotinamide [μM]
N
ADCP [%]
6-Aminonicotinamide [μM]
ADCP change [%]
6-Aminonicotinamide [μM]
O
ADCP [%]
6-Aminonicotinamide [μM]
ADCP change [%]
6-Aminonicotinamide [c] [μM]

Figure S2. Metabolic modulation changes antibody-dependent cellular phagocytosis (ADCP) of hMB cells by macrophages.

Related to Figure 1.

(A-O) ADCP rate and ADCP rate compared to basal phagocytosis rate (=ADCP change) under treatment with metabolic inhibitors in a co-culture of J774A.1 macrophages and hMB cells under antibody treatment with alemtuzumab. **A, D, G, J, M** J774A.1 macrophages pre-treated with metabolic inhibitor, **B, E, H, K, N** inhibitor treatment of the co-culture, **C, F, I, L, O** hMB cells pre-treated with metabolic inhibitor. Used inhibitors **A-C** 2-deoxy-D-glucose, **D-F** BML275, **G-I** oligomycin, **J-L** oxythiamine, **M-O** 6-aminonicotinamide.

Technical replicates n=15-58, biological replicates n=3-12. Data are shown as mean ± SEM. *P* values were calculated using one-way ANOVA. *$p < 0.05$; **$p < 0.01$; ***$p < 0.001$; ****$p < 0.0001$.

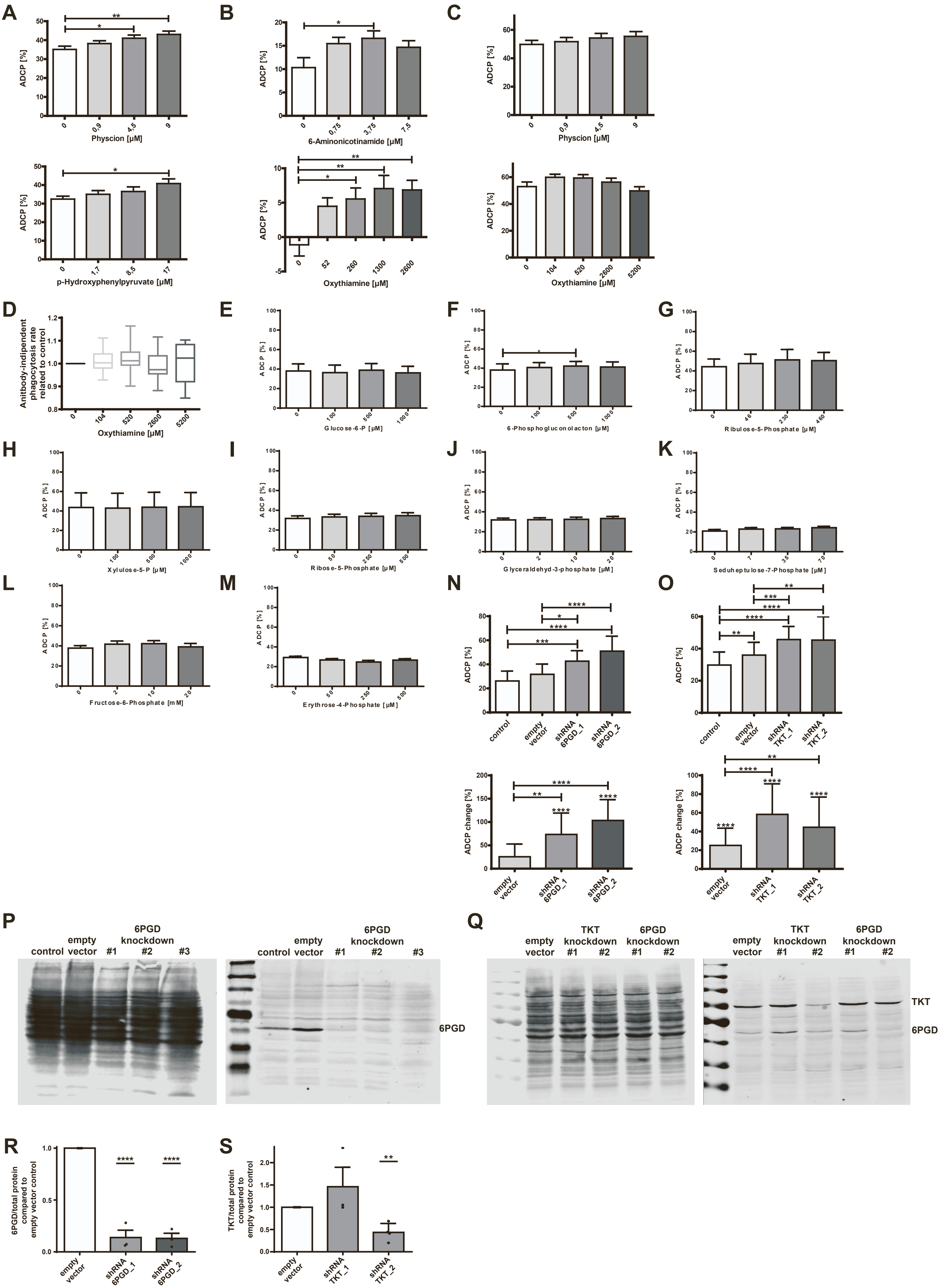
A
B
C
D
E
F
G
H
I
J
K
L
M
N
O
P
Q
R
S
ADCP [%]
Physcion [µM]
6-Aminonicotinamide [µM]
p-Hydroxyphenylpyruvate [µM]
Oxythiamine [µM]
Anitbody-indipendent phagocytosis rate related to control
Glucose-6-P [µM]
6-Phosphogluconolacton [µM]
Ribulose-5-Phosphate [µM]
Xylulose-5-P [µM]
Ribose-5-Phosphate [µM]
Glyceraldehyd-3-phosphate [µM]
Seduheptulose-7-Phosphate [µM]
Fructose-6-Phosphate [mM]
Erythrose-4-Phosphate [µM]
ADCP change [%]
control
empty vector
shRNA 6PGD_1
shRNA 6PGD_2
shRNA TKT_1
shRNA TKT_2
6PGD knockdown
TKT knockdown
#1
#2
#3
TKT
6PGD
6PGD/total protein compared to empty vector control
TKT/total protein compared to empty vector control

Figure S3. PPP modulation changes ADCP of hMB cells by macrophages.

Related to Figure 2.

(A, C) ADCP rate and ADCP rate compared to basal phagocytosis rate (=ADCP change) under treatment with metabolic inhibitors in a co-culture of J774A.1 macrophages and hMB cells under antibody treatment with alemtuzumab. **A** ADCP performed under PPP inhibition with physcion or p-hydroxyphenylpyruvate, **B** ADCP performed with THP1 monocytes and hMB cells under antibody treatment with obinutuzumab and PPP inhibition with 6-aminonicotinamide or oxythiamine. **C** ADCP assay performed in hypoxia under PPP inhibition with physcion or oxythiamine. **D** antibody-independent cellular phagocytosis (AiCP) rate of hMB cells by J744A.1 macrophages under treatment with oxythiamine. **(E-M)** ADCP rate under supplementation of PPP intermediates. **E** glucose-6-phosphate, **F** 6-phosphogluconolactone, **G** ribulose-5-phosphate, **H** xylulose-5-phosphate, **I** ribose-5-phosphate, **J** glyceraldehyde-3-phosphate, **K** sedoheptulose-7-phosphate, **L** fructose-6-phosphate, **M** erythrose-4-phosphate. **(N-O)** ADCP rate and ADCP rate compared to basal phagocytosis rate (=ADCP change) of hMB cells by shRNA mediates PPP knockdown macrophages**. N** shRNA mediated knockdown of 6-phosphogluconate dehydrogenase, **O** shRNA mediated knockdown of transketolase. **(P)** One representative example of western blot analysis of J744A.1 macrophages transfected with empty vector control and shRNA targeting 6-phosphogluconate dehydrogenase. Total protein stain and staining of 6-phosphogluconate dehydrogenase. **(Q)** One representative example of western blot analysis of J744A.1 macrophages transfected with empty vector control and shRNA targeting transketolase. Total protein stain and staining of transketolase. **(R)** Western blot analysis of 6pgd expression in J774A.1 macrophages under shRNA mediated PPP knockdown of 6Pgd compared to empty vector control. **(S)** Western blot analysis of Tkt expression in J774A.1 macrophages under shRNA mediated PPP knockdown of 6Pgd compared to empty vector control.

Technical replicates n=13-30; biological replicates n=3-6. In **A-O, R-S** data are shown as mean ± SEM. *P* values were calculated in **A-O** using one-way ANOVA, in **R-S** using Unpaired t-test. *$p < 0.05$; **$p < 0.01$; ***$p < 0.001$; ****$p < 0.0001$.

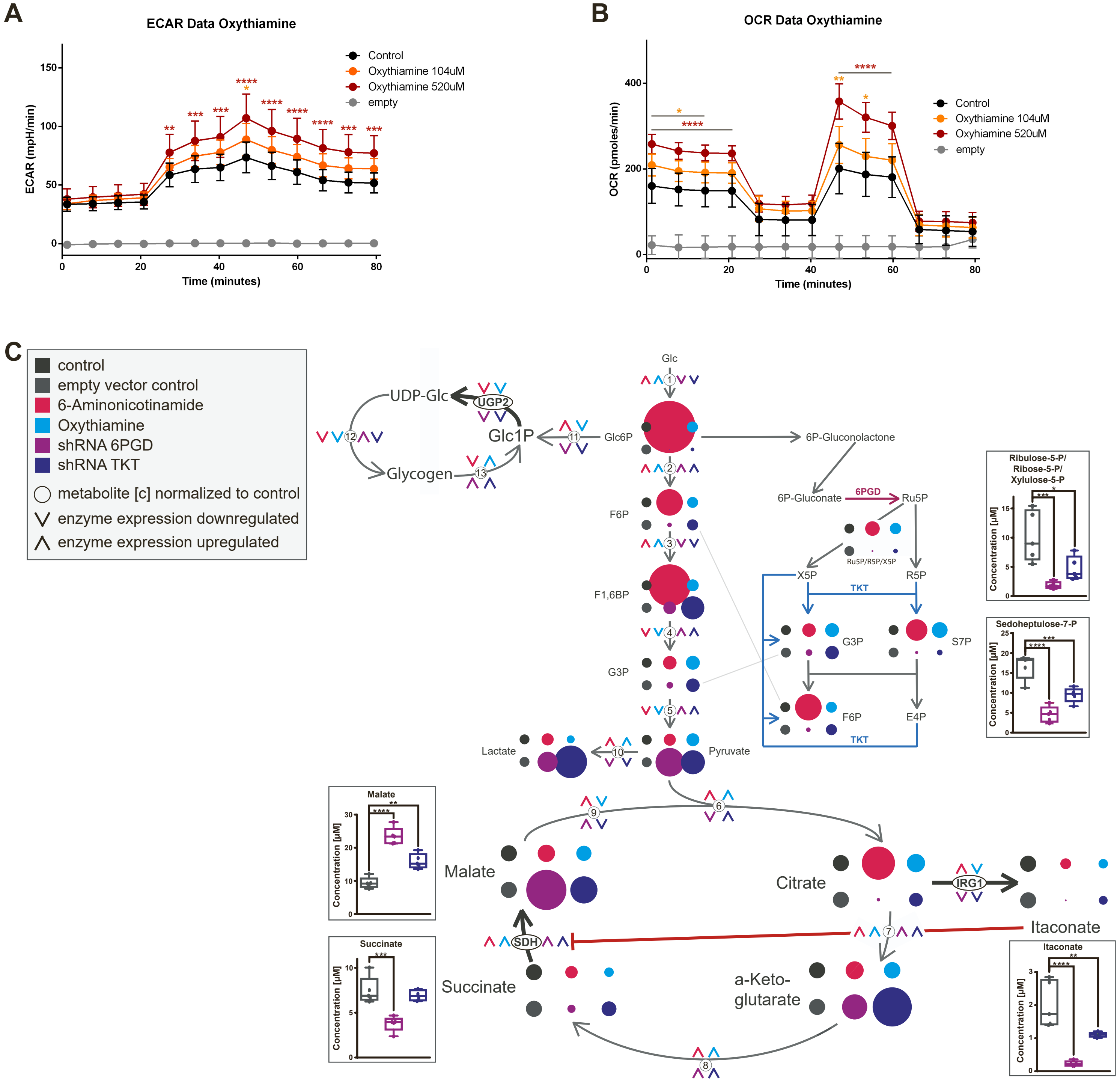

A
ECAR Data Oxythiamine
ECAR (mpH/min)
Time (minutes)
Control
Oxythiamine 104uM
Oxythiamine 520uM
empty
B
OCR Data Oxythiamine
OCR (pmoles/min)
Time (minutes)
Control
Oxythiamine 104uM
Oxyhiamine 520uM
empty
C
control
empty vector control
6-Aminonicotinamide
Oxythiamine
shRNA 6PGD
shRNA TKT
metabolite [c] normalized to control
enzyme expression downregulated
enzyme expression upregulated
Glc
Glc6P
Glc1P
UDP-Glc
UGP2
Glycogen
6P-Gluconolactone
6P-Gluconate
6PGD
Ru5P
Ru5P/R5P/X5P
X5P
R5P
TKT
G3P
S7P
F6P
E4P
F1,6BP
Lactate
Pyruvate
Malate
Citrate
IRG1
Itaconate
SDH
Succinate
a-Keto-
glutarate
Ribulose-5-P/
Ribose-5-P/
Xylulose-5-P
Sedoheptulose-7-P
Malate
Succinate
Itaconate
Concentration [µM]

Figure S4. PPP modulation changes metabolic activity in macrophages.

Related to Figures 3 and 5.

(A-B) Measurement of metabolic activity of J774A.1 macrophages under drug mediated PPP inhibition with oxythiamine by SeaHorse analysis. **A** one representative example of MitoStress test measurement of ECAR, **B** one representative example of MitoStress test measurement of OCR. **C** Metabolomic analysis of central metabolic pathways with overlay of proteomics data under compound mediated PPP inhibition compared to untreated J774A.1 macrophages and shRNA mediated PPP knockdown of 6Pgd and Tkt compared to empty vector control J774A.1 macrophages. Relative metabolite abundance compared to respective control represented in circle size. Absolute amount of metabolites of interest displayed in bar graphs. Change in enzyme expression assessed by proteomics displayed in arrow direction. Arrow upwards: increased enzyme expression compared to respective control; arrow downwards: decreased enzyme expression compared to respective control. Inhibited enzyme reactions by compounds or shRNA mediated knockdown coloured in violet (6Pgd) and blue (Tkt). *Metabolites: E4P* erythrose-4-phosphate, *F1,6BP* fructose-1,6-bisphosphate, *F6P* fructose-6-phosphate, *G3P* glyceraldehyde-3-phosphate, *Glc* glucose, *Glc1P g*lucose-1-phosphate, *Glc6P* glucose-6-phosphate, *R5P* ribose-5-phosphate, *Ru5P* ribulose-5-phosphate, *S7P* sedoheptulose-7-phosphate, *UDP-Glc* UDP-glucose, *X5P* xylulose-5-phosphate. *Enzymes: Irg1* immune-regulatory gene 1, *Sdh* succinate dehydrogenase, *Ugp2* UDP-glucose pyrophosphorylase 2, *1)* hexokinase, *2)* glucose-6-phosphate isomerase, *3)* phosphofructokinase, *4)* aldolase, *5)* sum up of glycerinaldehyde-3-phosphate dehydrogenase, phosphoglycerate kinase, enolase, pyruvate kinase, *6)* citrate synthase, *7)* sum up of aconitase, isocitrate dehydrogenase, *8)* sum up of a-ketoglutarate dehydrogenase, succinyl-CoA synthetase, *9)* malate dehydrogenase, *10)* lactate dehydrogenase, *11)* phosphoglucomutase 1, *12)* UTP-glucose-1-phosphate uridyltransferase, *13)* glycogen phosphorylase.

Technical replicates **A**-**B** n=6-27, **C** n=3; biological replicates **A**-**B** n=1-9, **C** n=3. In **A-B** data are shown as mean of six replicates in one experiment ± SD, n=6, in **C** data are shown as Min. to Max, n=3. *P* values were calculated using one-way ANOVA. *$p < 0.05$; **$p < 0.01$; ***$p < 0.001$; ****$p < 0.0001$.

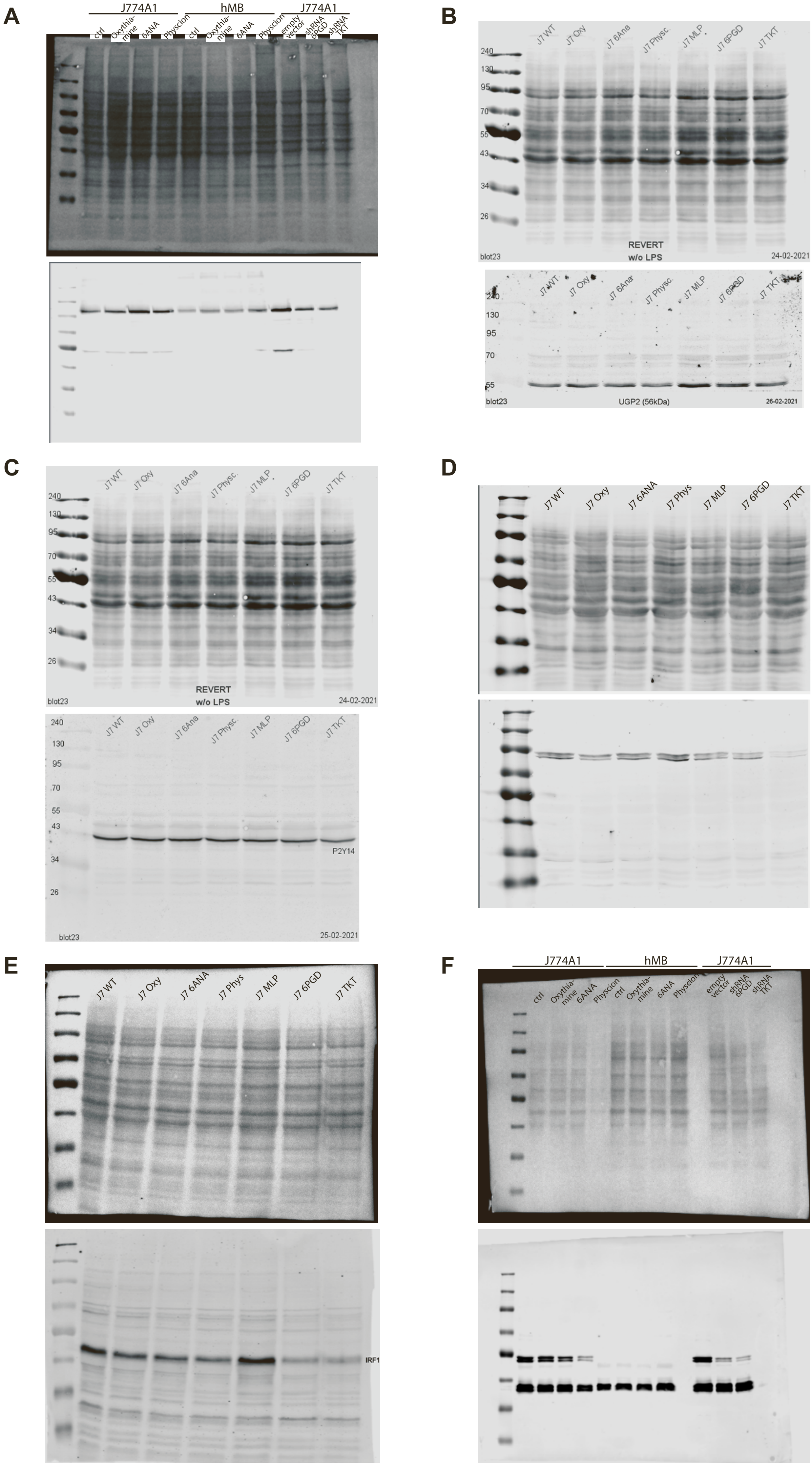

A
J774A1
hMB
J774A1
ctrl
Oxythia-mine
6ANA
Physcion
empty vector
shRNA 6PGD
shRNA TKT
B
J7 WT
J7 Oxy
J7 6Ana
J7 Physc.
J7 MLP
J7 6PGD
J7 TKT
240
130
95
70
55
43
34
26
REVERT
w/o LPS
blot23
24-02-2021
UGP2 (56kDa)
26-02-2021
C
P2Y14
25-02-2021
D
J7 6ANA
J7 Phys
E
IRF1
F

Figure S5. PPP inhibition changes the protein expression of hypothesized metabolic-immune response axis in macrophages.

Related to Figures 4 and 5.

(A-F) One representative example of western blot analysis of J744A.1 macrophages after drug mediated inhibition of the PPP or shRNA mediated knockdown of the PPP. Total protein stain and staining of protein of interest are shown. In **A** and **F** also hMB cells under PPP inhibition has been tested. **A** PYK2 staining, **B** UGP2 staining, **C** P2Y14 staining, **D** STAT1 staining, **E** IRF1 staining, **F** IRG1 staining.

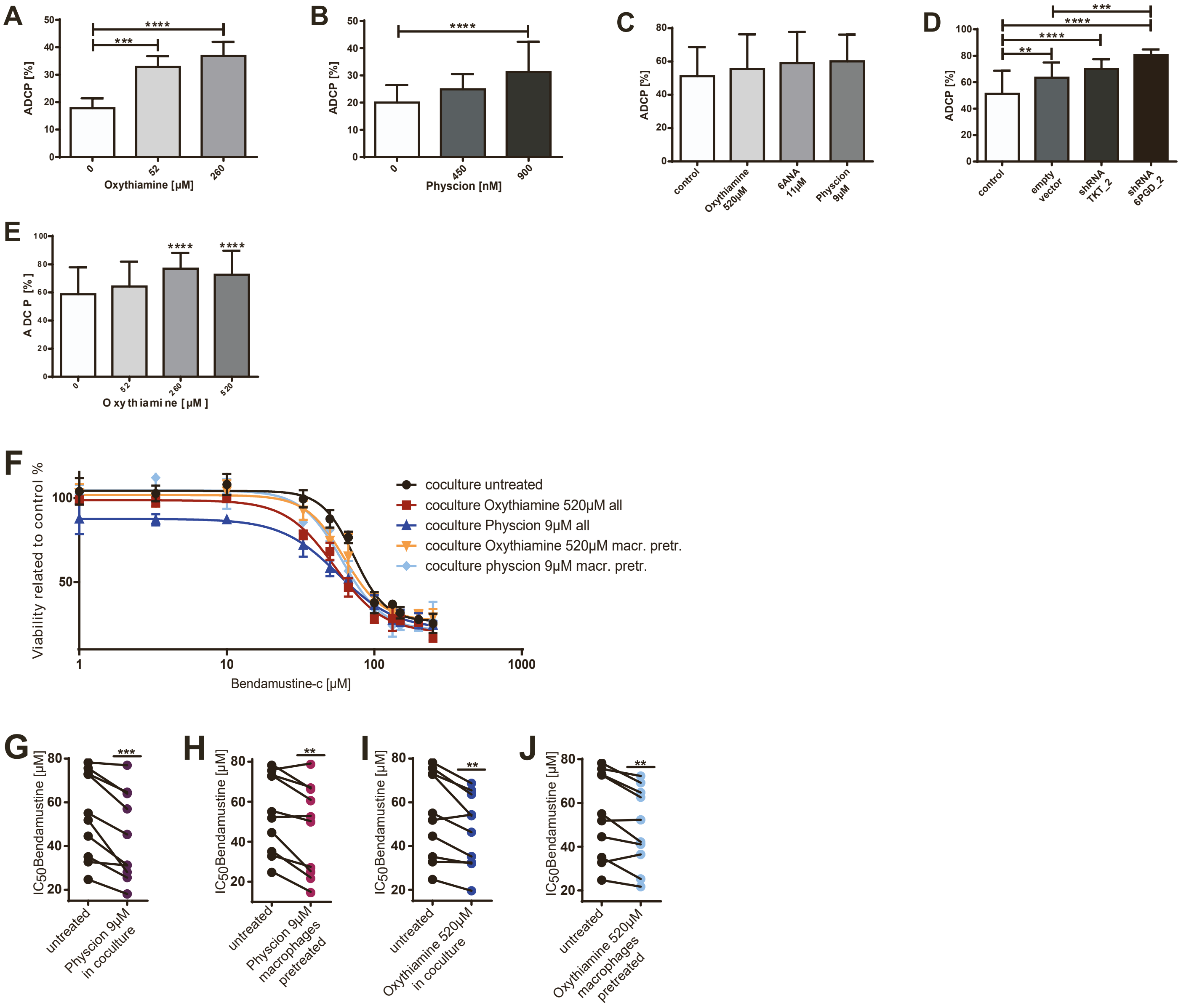
A
ADCP [%]
Oxythiamine [µM]
B
Physcion [nM]
C
control
Oxythiamine 520µM
6ANA 11µM
Physcion 9µM
D
control
empty vector
shRNA TKT_2
shRNA 6PGD_2
E
Oxythiamine [µM]
F
Viability related to control %
Bendamustine-c [µM]
coculture untreated
coculture Oxythiamine 520µM all
coculture Physcion 9µM all
coculture Oxythiamine 520µM macr. pretr.
coculture physcion 9µM macr. pretr.
G
IC50Bendamustine [µM]
untreated
Physcion 9µM in coculture
H
Physcion 9µM macrophages pretreated
I
Oxythiamine 520µM in coculture
J
Oxythiamine 520µM macrophages pretreated

Figure S6. PPP inhibition in primary human environment increases phagocytic capacity of macrophages and favours primary CLL cells chemotherapy sensitivity.

Related to Figure 6.

(A-B) ADCP rate of primary human monocyte derived macrophages differentiated in the presence of PPP inhibitors. **A** ADCP change by primary monocyte derived macrophages differentiated in the presence of physcion and M-CSF **B** ADCP change by primary monocyte derived macrophages differentiated in the presence of oxythiamine and M-CSF. **(C-D)** ADCP rate of primary CLL patient cells by J774A.1 macrophages. **C** ADCP rate under drug mediated PPP inhibition, **D** ADCP rate under shRNA mediated PPP knockdown. **(E)** ADCP rate of primary CLL patient cells by primary human monocyte derived macrophages differentiated in the presence of oxythiamine and M-CSF. **(F)** One representative example of dose response curve of individual orimary CLL patient cell samples towards bendamustine treatment. Cells were incubated with bendamustine after protective macrophage co-culture with untreated J774A.1 macrophages vs. PPP inhibition. **(G-J)** Dose-response curve (IC_{50}) for individual primary CLL patient cell samples to bendamustine treatment. Cells were incubated with bendamustine after protective macrophage co-culture with untreated J774A.1 macrophages vs. PPP inhibition. **G-H** Inhibition of 6Pgd in oxidative part of PPP by physcion, **G** co-culture treatment, **H** macrophage pre-treatment. **I-J** Inhibition of Tkt in non-oxidative part of PPP by oxythiamine, **I** co-culture treatment, **J** macrophage pre-treatment.

Technical replicates **A** n=28, **B** n=20, **C-D** n=20, **E** n=65, **F** n=30, **G-J** n=30; biological replicates **A** n=6, **B** n=4, **C-D** n=5, **E** n=12, **F** n=10, **G-J** n=10. Data are shown as mean ± SEM. *P* values were calculated in **A-E** by using one-way ANOVA, in **G-J** using paired t-test. *$p < 0.05$; **$p < 0.01$; ***$p < 0.001$; ****$p < 0.0001$.

A

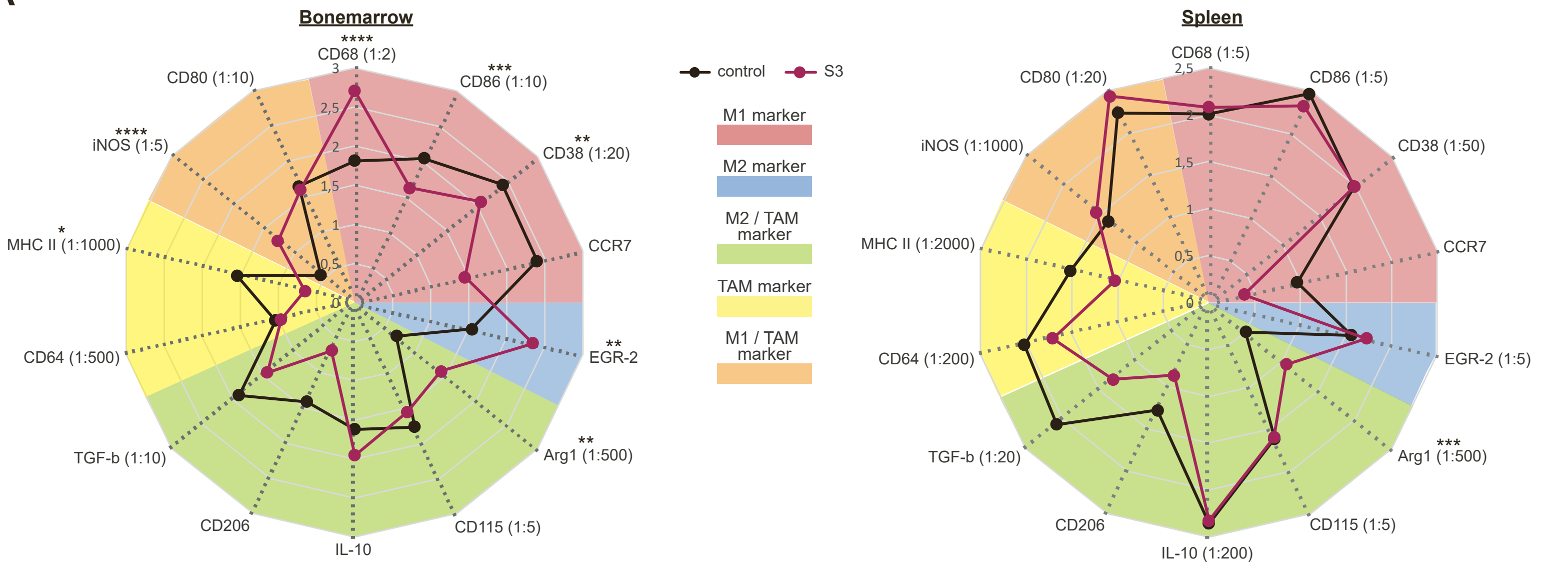

B

Benjamini-Hochberg-analysis

	vehicle	alemtuzumab	S3 + alemtuzumab
vehicle	**--**	**1.4e-05**	**--**
S3	**0.6159**	**5.5e-05**	**1.0e-07**
S3 + alemtuzumab	**3.4e-09**	**0.0059**	**--**

C

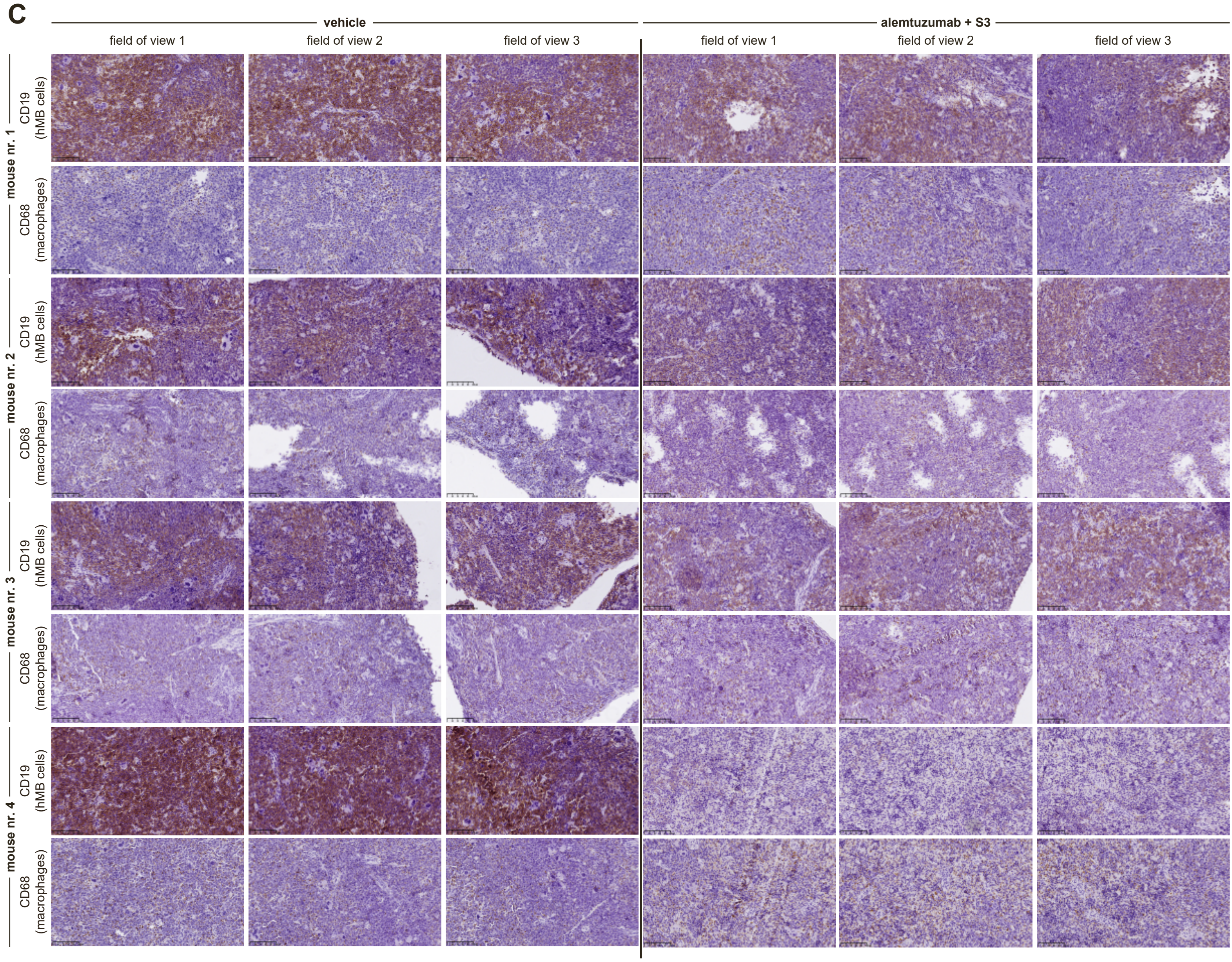

Figure S7. PPP inhibition increases myelopoiesis and macrophages´ activity *in vivo* and improves treatment response in an aggressive humanized lymphoma mouse model.

Related to Figure 7.

(A) Expression of characteristic surface marker for different macrophage subtypes on macrophages in bone marrow and spleen. C57BL/6 mice treated with vehicle (control) or S3 i.p. for 7 days. **(B)** Significance testing by using Benjamini-Hochberg-analysis of survival curves of NSG mice transfected with hMB and treated after three days of engraftment with vehicle, alemtuzumab and/or PPP inhibitor S3 for 12 days. **(C)** Immunohistochemical staining of hMB cells ($CD19^+$) and macrophages ($CD68^+$) in spleen of NSG mice transfected with hMB and treated after three days of engraftment with vehicle or alemtuzumab + S3 for 12 days.

Technical replicates **A** n=9-10, **B** n=21-25, **C** n=4; biological replicates **A** n=9-10, **B** n=21-25, **C** n=4. In **A** data are shown as mean of ten replicates. In **A** *P* values were calculated by using one-way ANOVA. $*p < 0.05$; $**p < 0.01$; $***p < 0.001$; $****p < 0.0001$.

Table S6. Qualifier and quantifier transition of metabolites measured by targeted LC-QqQ/MS analysis. Related to Figure 5.

Compound name	Transition (m/z)	Transition type	Fragmentor (V)	Collision Energy (eV)	Cell accelerator (V)	RT (min)
Succinic acid D6 (ISTD)	121 → 77	quantifier	77	10	4	11.9
Glutathione - glycine-$^{13}C_2$,^{15}N trifluor (ISTD)	309 → 146	quantifier	119	14	4	12.3
	309 → 128	qualifier	119	14	4	12.3
Adenosine 5-diphosphate	426 → 328	quantifier	56	16	4	17.9
	426 → 159	qualifier	56	28	4	17.9
Adenosine 5-triphosphate	506 → 408	quantifier	122	22	4	20.5
	506 → 159	qualifier	122	38	4	20.5
Lactic acid	89 → 45	qualifier	48	9	4	3.0
	89 → 43	quantifier	48	10	4	3.0
Pyruvic Acid	87 → 43	quantifier	48	4	4	2.2
alpha - ketoglutaric acid	145 → 101	quantifier	70	5	4	10.6
	145 → 57	qualifier	70	9	4	10.6
NAD	662 → 540	quantifier	70	12	4	14.4
	662 → 328	qualifier	70	36	4	14.4
D-Fructose 6-phosphate	259 → 97	quantifier	102	14	4	16.3
	259 → 79	qualifier	102	48	4	16.3
D-Glucose-6-phosphate	259 → 79	quantifier	102	48	4	18.5
	259 → 97	qualifier	102	14	4	18.5
Ribose 5-phosphate / Ribulose 5-phosphate	229 → 97	qualifier	96	10	4	14.3
	229 →79	quantifier	96	48	4	14.3
D-Xylulose 5-phosphate / Ribose 5-phosphate / Ribulose 5-phosphate	229 → 139	qualifier	86	8	4	15.3
	229 → 79	quantifier	96	48	4	15.3
D-Sedoheptulose 7-phosphate	289 → 97	quantifier	104	18	4	17.0
	289 → 79	qualifier	104	48	4	17.0
Glutathione Reduced	306 → 143	quantifier	109	14	4	12.3
	306 → 128	qualifier	109	14	4	12.3
NADP	742 → 620	quantifier	79	10	4	24.9
	742 → 408	qualifier	79	38	4	24.9
Succinic acid	117 → 99	qualifier	62	8	4	11.9
	117 → 73	quantifier	62	10		11.9
Itaconic acid	129 → 85	quantifier	68	6	4	8.4
	129 → 41	qualifier	68	12	4	8.4
L-Malic acid	133 → 115	quantifier	76	8	4	12.8
	133 → 71.1	qualifier	76	14	4	12.8
Citric acid	191 → 111	quantifier	78	10	4	17.5
	191 → 87	qualifier	78	16	4	17.5

NADH	664 → 408	qualifier	190	29	4	11.7
	664 → 79	quantifier	190	49	4	11.7
DL-Glyceraldehyde 3-phosphate	169 → 97	quantifier	130	4	4	14.7
	169 → 79	qualifier	130	28	4	14.7
D-Fructose 1,6-biphosphate	339 → 241	qualifier	90	12	4	25.9
	339 → 97	quantifier	90	22	4	25.9
6-phosphogluconic acid	275 → 79	quantifier	109	49	4	21.7
D-erytrose 4-phosphate	199 → 97	quantifier	151	6	4	13.9
	199 → 79	qualifier	151	30	4	13.9
